# A round-robin approach provides a detailed assessment of biomolecular small-angle scattering data reproducibility and yields consensus curves for benchmarking

**DOI:** 10.1107/S2059798322009184

**Published:** 2022-10-20

**Authors:** Jill Trewhella, Patrice Vachette, Jan Bierma, Clement Blanchet, Emre Brookes, Srinivas Chakravarthy, Leonie Chatzimagas, Thomas E. Cleveland, Nathan Cowieson, Ben Crossett, Anthony P. Duff, Daniel Franke, Frank Gabel, Richard E. Gillilan, Melissa Graewert, Alexander Grishaev, J. Mitchell Guss, Michal Hammel, Jesse Hopkins, Qingqui Huang, Jochen S. Hub, Greg L. Hura, Thomas C. Irving, Cy Michael Jeffries, Cheol Jeong, Nigel Kirby, Susan Krueger, Anne Martel, Tsutomu Matsui, Na Li, Javier Pérez, Lionel Porcar, Thierry Prangé, Ivan Rajkovic, Mattia Rocco, Daniel J. Rosenberg, Timothy M. Ryan, Soenke Seifert, Hiroshi Sekiguchi, Dmitri Svergun, Susana Teixeira, Aurelien Thureau, Thomas M. Weiss, Andrew E. Whitten, Kathleen Wood, Xiaobing Zuo

**Affiliations:** a School of Life and Environmental Sciences, The University of Sydney, Sydney, NSW 2006, Australia; b Université Paris-Saclay, CEA, CNRS, Institute for Integrative Biology of the Cell (I2BC), Paris, 91198 Gif-sur-Yvette, France; c Molecular Biophysics and Integrated Bioimaging Division, Lawrence Berkeley National Laboratory, Berkeley, California, USA; d European Molecular Biology Laboratory (EMBL) Hamburg Unit, Notkestrasse 85, c/o Deutsches Elektronen-Synchrotron, 22607 Hamburg, Germany; e Chemistry and Biochemistry, University of Montana, 32 Campus Drive, Missoula, MT 59812, USA; f BioCAT, Department of Biological Sciences, Illinois Institute of Technology, Chicago, IL 60616, USA; g Theoretical Physics and Center for Biophysics, Saarland University, Campus E2.6, 66123 Saarbrücken, Germany; h Institute for Bioscience and Biotechnology Research, 9600 Gudelsky Drive, Rockville, MD 20850, USA; i National Institute of Standards and Technology, 100 Bureau Drive, Gaithersburg, MD 20899, USA; j Diamond Light Source, Harwell Science and Innovation Campus, Didcot OX11 0DE, United Kingdom; k Sydney Mass Spectrometry, The University of Sydney, Sydney, NSW 2006, Australia; l Australian Nuclear Science and Technology Organisation, New Illawara Road, Lucas Heights, NSW 2234, Australia; m Institut de Biologie Structurale, CEA, CNRS, Université Grenoblé Alpes, 41 Rue Jules Horowitz, 38027 Grenoble, France; n Cornell High-Energy Synchrotron Source, 161 Synchrotron Drive, Ithaca, NY 14853, USA; o Department of Physics, Wesleyan University, Middletown, CT 06459, USA; p Australian Synchrotron, ANSTO, 800 Blackburn Road, Clayton, VIC 3158, Australia; q Institut Laue–Langevin, 71 Avenue des Martyrs, 38042 Grenoble CEDEX 9, France; r Stanford Synchrotron Radiation Lightsource, Stanford University, 2575 Sand Hill Road, Menlo Park, CA 94025, USA; s National Facility for Protein Science in Shanghai, Zhangjiang Laboratory, Shanghai Advanced Research Institute, Chinese Academy of Sciences, Road No. 333, Haike Road, Shanghai 201210, People’s Republic of China; t Synchrotron SOLEIL, L’Orme des Merisiers, Saint-Aubin BP 48, 91192 Gif-sur-Yvette, France; u CITCoM (UMR 8038 CNRS), Faculté de Pharmacie, 4 Avenue de l’Observatoire, 75006 Paris, France; v Proteomica e Spettrometria di Massa, IRCCS Ospedale Policlinico San Martino, Largo R. Benzi 10, 16132 Genova, Italy; w X-ray Science Division, Advanced Photon Source, Argonne National Laboratory, Lemont, IL 60439, USA; x SPring-8, Japan Synchrotron Radiation Research Institute, 1-1-1 Kouto, Sayo-cho, Sayo-gun, Hyōgo 679-5198, Japan; y Department of Chemical and Biomolecular Engineering, University of Delaware, 150 Academy Street, Newark, DE 19716, USA; University of Western Australia, Crawley, Australia

**Keywords:** biomolecular small-angle scattering, X-ray scattering, neutron scattering, standards, benchmarking standards, scattering-profile calculation

## Abstract

Through an expansive international effort that involved data collection on 12 small-angle X-ray scattering (SAXS) and four small-angle neutron scattering (SANS) instruments, 171 SAXS and 76 SANS measurements for five proteins (ribonuclease A, lysozyme, xylanase, urate oxidase and xylose isomerase) were acquired. From these data, the solvent-subtracted protein scattering profiles were shown to be reproducible, with the caveat that an additive constant adjustment was required to account for small errors in solvent subtraction. Further, the major features of the obtained consensus SAXS data over the *q* measurement range 0–1 Å^−1^ are consistent with theoretical prediction. The inherently lower statistical precision for SANS limited the reliably measured *q*-range to <0.5 Å^−1^, but within the limits of experimental uncertainties the major features of the consensus SANS data were also consistent with prediction for all five proteins measured in H_2_O and in D_2_O. Thus, a foundation set of consensus SAS profiles has been obtained for benchmarking scattering-profile prediction from atomic coordinates. Additionally, two sets of SAXS data measured at different facilities to *q* > 2.2 Å^−1^ showed good mutual agreement, affirming that this region has interpretable features for structural modelling. SAS measurements with inline size-exclusion chromatography (SEC) proved to be generally superior for eliminating sample heterogeneity, but with unavoidable sample dilution during column elution, while batch SAS data collected at higher concentrations and for longer times provided superior statistical precision. Careful merging of data measured using inline SEC and batch modes, or low- and high-concentration data from batch measurements, was successful in eliminating small amounts of aggregate or interparticle interference from the scattering while providing improved statistical precision overall for the benchmarking data set.

## Abstract 2

Small-angle X-ray scattering (SAXS) and small-angle neutron scattering (SANS) measurements of five standard proteins in solution using 12 SAXS and four SANS instruments demonstrate reproducibility and yield consensus scattering profiles that provide a foundation benchmarking set to evaluate approaches to scattering-profile prediction from atomic coordinates.

## 1. Introduction

Biomolecular small-angle scattering (SAS) has enjoyed decades of continuing growth in its impact on structural biology (for recent reviews, see Koch *et al.*, 2003 ▸; Jacques & Trewhella, 2010 ▸; Trewhella, 2016 ▸, 2022 ▸; Tuukkanen *et al.*, 2017 ▸; Mahieu & Gabel, 2018 ▸; Brosey & Tainer, 2019 ▸; Da Vela & Svergun, 2020 ▸). The elastic, coherent scattering profile from a solution of monodisperse, non-interacting biological molecules of uniform size yields structural parameters such as the radius of gyration (*R*_g_), the molecular volume (for example as the Porod volume *V*_P_) and the distribution of interatomic distances [*P*(*r*) versus *r*] that includes an estimate of the maximum linear dimension (*d*_max_) (for comprehensive texts, see Svergun *et al.*, 2013 ▸; Chaudhuri *et al.*, 2017 ▸; Lattman *et al.*, 2018 ▸). The full utilization of SAS data to gain biological insights, however, depends upon the ability to accurately predict or simulate the SAS profile from atomic coordinates for comparison with measurements. Since the publication of the first programs to calculate small-angle X-ray scattering (SAXS; Svergun *et al.*, 1995 ▸) and small-angle neutron scattering (SANS; Svergun *et al.*, 1998 ▸) profiles from atomic coordinates there has been an ongoing acceleration in the rate of biomolecular SAS publications and citations (Trewhella, 2022 ▸). Since these first programs, there have been numerous further developments and new approaches to SAS profile prediction (see, for example, Grishaev *et al.*, 2010 ▸; Poitevin *et al.*, 2011 ▸; Chen & Hub, 2014 ▸; Schneidman-Duhovny *et al.*, 2016 ▸; Grudinin *et al.*, 2017 ▸; Hub, 2018 ▸), including an extension to ensembles for dynamic and multistate systems (see, for example, Bernadó *et al.*, 2007 ▸; Schneidman-Duhovny *et al.*, 2016 ▸; Cordeiro *et al.*, 2017 ▸). Each developer has chosen a preferred set of experimental data against which to test their approach as there is no standard set of data to evaluate the differences among the different approaches or to test new approaches in a standard way. In addition, the very use of SAS data for structural analysis implies an assumption of their reproducibility: data sets collected independently using different instruments from the same biomolecule in the same solution conditions are assumed to coincide within experimental error. However, no such demonstration has ever been carried out.

The aim of this project was to generate a set of experimental SAS profiles for proteins of known structure that can be used to benchmark different approaches to calculating SAS profiles from atomic coordinates while also testing the intrinsic reproducibility of the experiment. To this end, SAS profiles for five proteins were measured on different beamlines using a common source for each protein and standard buffers. Each protein was measured using SAXS as well as SANS with H_2_O and D_2_O buffers. These three sets of data are influenced by distinct scattering-contrast values for the protein and its hydration layer with respect to the bulk solvent and potentially could be used to test different models of the hydration layer (Svergun *et al.*, 1998 ▸; Zhang *et al.*, 2012 ▸; Kim & Gabel, 2015 ▸).

Sets of data were submitted to the project coordinators (JT and PV) for assessment as scattered intensity *I* as a function of the momentum transfer or scattering-vector amplitude *q* [*i.e.* *I*(*q*) versus *q*, where *q* = (4πsinθ)/λ, θ is half of the scattering angle and λ is the wavelength of the radiation] with associated standard errors for [Sample + Solvent], [Solvent] and [Sample + Solvent] − [Solvent]. Data over the widest *q*-range possible with accurate error propagation were requested for the benchmarking goal. Initial assessment included evaluation of the Guinier-derived *R*_g_, *P*(*r*)-derived *R*_g_, *d*_max_ and *V*_P_ values compared with expected values based on the known sequence and crystal structure of each protein to identify potential problems, such as sample aggregation or interparticle interference. The experimental reproducibility was then assessed and consensus scattering profiles were calculated and compared with theoretical predictions.

## 2. Criteria for selection of proteins

The selection of suitable proteins initially focused on identifying structures that were relatively rigid in order to avoid possible complications due to flexible regions or structural inhomogeneity. Further, there should be high-resolution crystal structures of good quality for each protein, and a range of sizes and shapes was desirable. Also, the selected proteins needed to be readily available at high purity with conditions for optimal SAS data collection available from previous studies to minimize the potential for interparticle interference or aggregation that would bias the results.

The search for proteins that could meet the above conditions proved to be challenging, not least because dynamics to some degree play an essential role in nearly all of biology. Added to the demanding criteria was the fact that the preparation of ideal, dilute solutions on a scale to enable this project was nontrivial. Samples and buffers were shipped internationally from a common source to control for solution variability as much as was practical. While shipping of samples has become more commonplace for users of large-scale facilities ahead of their scheduled experiments, in this case the required use of discretionary beam time by many participants meant that with heavily oversubscribed beam schedules some measurements could not be made for as long as nine months after sample shipment. Conditions for stable storage were thus also important.

**Figure 1:**
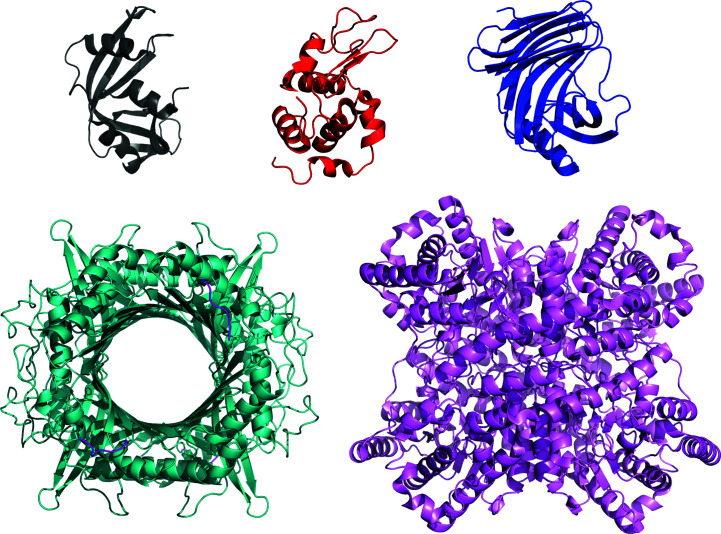
Ribbon representations of the crystal structures of RNaseA (PDB entry 7rsa, black), lysozyme (PDB entry 2vb1, red), xylanase (PDB entry 2dfc, blue), urate oxidase (PDB entry 3l8w, dark cyan, with added C-terminal SLKSKL in magenta) and xylose isomerase (PDB entry 1mnz, purple).

The proteins that were ultimately selected for the study included the three relatively small proteins (<20 kDa) ribonuclease A (RNaseA), lysozyme and xylanase, and two larger proteins (>30 kDa) that each form stable homotetramers (urate oxidase and xylose isomerase, also known as glucose isomerase). Ribbon representations of the crystal structures of each protein demonstrate the relative sizes and shapes of each protein and the fact that urate oxidase has a large central cavity that is solvent-accessible (Fig. 1 ▸). Bovine serum albumin was considered given its popularity as an intensity standard for SAS studies of proteins, but was not selected due to the flexibility of the loop connecting its two domains and its known tendency to oligomerize in solution over time (Bujacz, 2012 ▸; Trewhella *et al.*, 2017 ▸). Details of each of the selected proteins are provided in Table 1 ▸ and Supplementary Table S1, while Supplementary Table S2 gives the sequences, with modifications and bound ligands, of the proteins used for measurement.

## 3. Experimental protocols

### 3.1. Sample preparation

While the intent was to have a single source and uniform sample handling, in the final analysis there was some variability due to multiple factors. The concentration ranges for each protein measured varied from 0.1 to 10 mg ml^−1^ depending on the characteristics of the individual beamlines. Some additional measurements also were performed using locally sourced lysozyme that were included in the final analysis as there was no significant difference in the measured scattering profiles compared with the centrally provided lysozyme. Also, during implementation some participants modified the solvent conditions, for example to prevent capillary fouling at the very high brightness of one beamline. Participants made decisions based on the capabilities of and experience at each beamline and these are noted in Supplementary Table S3. As it happened, there were no discernible effects from the adjustments to buffers and additives.

#### 3.1.1. Molecular-mass and purity checks

The xylanase and xylose isomerase, which were originally purchased from Hampton Research (kindly donated to the project by Tim Ryan and Nigel Kirby), had been stored for some years. Therefore, they were subjected to denaturing polyacrylamide gel electrophoresis (SDS–PAGE) and intact protein mass spectrometry as checks on purity and to ensure that no degradation had occurred. Recombinant urate oxidase from *Aspergillus flavus* was specifically prepared for this project (a gift from Sanofi–Aventis, Aramont, France; available under the brand name Fasturtek) and had to be shipped internationally in solution and subject to storage at 4°C, in some cases for several months. A sample of urate oxidase therefore was also subjected to intact protein mass spectrometry after shipment and a period of such storage.

The SDS–PAGE gel lanes with overloaded xylanase and xylose isomerase (Supplementary Fig. S1) showed a single dominant band at the expected molecular mass for the monomer, with some very weak higher molecular mass bands that are attributable to trace contaminants. The major observed masses for xylanase, urate oxidase and xylose isomerase are within 20 p.p.m. of the expected mass (Table 1 ▸), with additional minor peaks that are most likely to be sodium or potassium adducts (Supplementary Fig. S2).

#### 3.1.2. Preparation of buffers for RNaseA, lysozyme, xylanase and xylose isomerase

Buffered solutions for each protein were prepared in autoclaved bottles with filtering (0.22 µm filter) and transferred to sterile 50 ml Falcon tubes for transport to the participating laboratories as ∼40 ml aliquots to be diluted 1:10 using 18 MΩ cm^−1^ water and used in final dialysis steps or for column elution prior to SAS measurements. For SANS measurements, both H_2_O and D_2_O 10× buffer solutions were provided. Each diluted buffer was to be checked for pH and adjusted to the desired values (as per Table 1 ▸) as needed.

The free radical scavenger NaN_3_ (Harbour & Issler, 1982 ▸) [0.1%(*w*/*v*), ReagentPlus, 99.5%; Sigma–Aldrich catalogue No. S2002) was recommended to be added to buffers just prior to SAXS sample preparation due to its time-dependent degradation; however, this was not performed in most cases (Supplementary Table S3), in part because many facilities did not have the required safety protocols in place for handling azide.

#### 3.1.3. Preparation of RNaseA, lysozyme, xylanase and xylose isomerase

Approximately 15 mg of each protein accompanied by their 10× buffer solutions were shipped cold (that is, maintained at 2–8°C) on 11 June 2019 from the Australian Synchrotron (ANSTO) by special courier to the participating laboratories.

Xylanase and xylose isomerase were supplied as a 43%(*v*/*v*) glycerol stock (0.5 ml of 36 mg ml^−1^ protein) and an ammonium sulfate microcrystalline precipitate (0.5 ml of 33 mg ml^−1^ protein in 0.91 *M* ammonium sulfate), respectively. Prior to SAS measurements, these protein stocks were dialyzed with 3 × 2 h changes and a 1:50 volume ratio against locally prepared buffer (50 m*M* Tris pH 7.5, plus 1 m*M* MgCl_2_ for xylose isomerase only) with progressively decreasing glycerol [20%, 10% and 0%(*w*/*v*)] or salt (500, 250 and 150 m*M* NaCl), respectively. A final dialysis of 2 × 6 h changes was then performed with a 1:100 volume ratio against the measurement buffer provided together with the protein. This sequence ensures the sufficient removal of glycerol from the xylanase and of ammonium salt from the xylose isomerase.

RNaseA and lysozyme, which were supplied as commercial powders, were dissolved directly in the measurement buffer and dialyzed against the measurement buffer with 2 × 2 h changes and a 1:100 volume ratio. According to local practice, the solutions were spun in an Eppendorf centrifuge (or equivalent) for 5 min to remove potential dust/particles.

#### 3.1.4. Preparation of urate oxidase and its buffer

Urate oxidase in complex with its very high affinity inhibitor 8-azaxanthine (molar mass 153.10 g mol^−1^) with all four sites bound was specially prepared according to Retailleau *et al.* (2004 ▸). For SAS measurements, ∼5 mg of the complex in the measurement buffer (as 0.5 ml of a 10 mg ml^−1^ solution) with sufficient buffer for SEC–SAS and batch SAS measurement was shipped on ice to each laboratory on 11 June 2019. The protein is known to be extremely stable in the measurement buffer at 4°C (Commission du Médicament et des Dispositifs Médicaux Stériles, 2005 ▸). At the concentrations used for SAS measurements there is insignificant free inhibitor and hence there is no need for free inhibitor in the measurement buffer.

Prior to shipment, urate oxidase was subjected to size-exclusion high-performance liquid chromatography (SE-HPLC) using an S200 column to confirm that it was the pure tetramer with no significant higher order oligomers. Prior to batch SAS measurements, it was recommended that SEC could be performed on an aliquot to evaluate the monodispersity of the sample following transport and, if indicated, a SEC purification step with concentration as needed could be performed.

#### 3.1.5. SANS sample preparation

Compared with SAXS, SANS measurements typically require larger samples, longer data-acquisition times and preparation of samples in D_2_O. Each facility optimized their sample preparation locally. Also, lysozyme is more soluble in H_2_O compared with D_2_O (Broutin *et al.*, 1995 ▸), and to minimize the formation of aggregates or possible gelling it was dissolved first in H_2_O and then dialyzed into D_2_O or subjected to exchange on a column.

At the Institut Laue–Langevin (ILL) exchange of buffers was accomplished by recovering samples after SEC–SANS and reconcentrating as needed to measure in batch mode. At the National Institute of Standards and Technology (NIST) samples were subjected to SEC and measured directly after SEC without performing dialysis or concentrating. At the Australian Nuclear Science Organization (ANSTO) samples were first purified using SEC with the appropriate H_2_O buffer and the peak fractions were pooled, concentrated if required and dialyzed into H_2_O or D_2_O buffer prior to measurement. Additional SANS measurements were made at ANSTO on RNaseA and lysozyme after elution from SEC followed immediately by dialysis and measurement without concentrating.

Solvent blanks for SANS measurements were taken either from column elution flowthrough or the final dialysis step. Reported pH values are as measured in D_2_O and H_2_O; that is, no adjustment of pH values was made for measurements in D_2_O, as per modern practice.

### 3.2. Data acquisition and initial data-evaluation protocol

Full details of sample handling prior to SAS measurements and SAS data acquisition at each facility are provided in Supplementary Table S3. A total of 247 SAS profiles were submitted from 12 SAXS and four SANS instruments for initial evaluation, including 44 SEC–SAXS, 118 batch SAXS in H_2_O, nine batch SAXS in D_2_O, ten SEC–SANS (five each in H_2_O and D_2_O) and 36 and 30 batch SANS in H_2_O and D_2_O, respectively (Supplementary Table S4*a* ).

Guinier and *P*(*r*) analyses for the submitted data sets were analyzed by the project coordinators (JT and PV) using a standard protocol to facilitate the initial comparison of results. *autoRg* and *autoGNOM* (from *ATSAS* 3.0 and 3.1; Franke *et al.*, 2017 ▸; Manalastas-Cantos *et al.*, 2021 ▸) were used, and quoted errors are as reported by these routines. Generally, minimal adjustments were made to the selected Guinier ranges, mostly to make *q*_max_ *R*_g_ ≃ 1.3, while maximum linear dimension (*d*_max_) values were rounded to whole numbers (in Å). Also, where indicated the *d*_max_ value selected by *autoGNOM* was manually refined to avoid apparent truncation or overextension of the *r* range by ensuring that *P*(*r*) approaches *d*_max_ smoothly as a horizontal tangent. For folded globular proteins, the release of the *P*(0) = 0 constraint can be used to detect possible solvent-subtraction errors as shown by a significant difference. Where this *P*(0) = 0 test implied a solvent-subtraction error, the subtractions were adjusted. This adjustment was more generally needed for SANS data (see Section 3.6).

Multiple protein concentrations were commonly measured in batch mode; however, there were generally insufficient concentration points measured over a wide enough concentration range for reliable extrapolation to infinite dilution. Instead, the project coordinators selected the optimal data set for analysis to have the maximal signal to noise and minimal evidence of aggregation or interparticle interference based on assessment of the Guinier plots and *P*(*r*) transforms.

### 3.3. Developing the *datcombine* tool to combine data in a standard way

To develop a consensus scattering profile for each protein the *datcombine* tool was created and is now available in *ATSAS* 3.1.0. Data at the various instruments were generally collected on an arbitrary intensity scale and over a range of concentrations, thus requiring the application of a multiplicative scale factor before combination. It was also necessary to apply an additive constant to account for differences in the background due to small inaccuracies in solvent subtraction. These adjustments are ideally performed for data on the same *q*-scale. The *datcombine* tool thus takes a set of data recorded at the various participating facilities for a given protein and first re-grids them onto a common *q*-scale. For the SAXS data, a uniform Δ*q* = 0.005 Å^−1^ was used for RNaseA, lysozyme and xylanase, while for the larger urate oxidase and xylose isomerase finer Δ*q* grids were used to preserve more data points in the Guinier region; a uniform Δ*q* = 0.002 Å^−1^ was used for urate oxidase, while a graduated scale with Δ*q* = 0.001 Å^−1^ to *q* = 0.05 Å^−1^ followed by 0.002 Å^−1^ to *q* = 0.3 Å^−1^ and 0.004 Å^−1^ to *q* = 1 Å^−1^ was needed to accommodate the submitted data for xylose isomerase. For the SANS data, the lower *q* regions for all proteins were re-gridded to Δ*q* = 0.002 Å^−1^ and transitioned to 0.006 Å^−1^ at the *q*-value dictated by Δ*q* in the submitted data (between 0.02 and 0.08 Å^−1^). Scaling and constant adjustment of the re-gridded data were implemented using the Levenberg–Marquardt minimization (Moré *et al.*, 1984 ▸) of all pairwise χ^2^ comparisons, an expression equivalent to minimizing the objective function *f* (equation 1), to determine the scaling coefficients *a_j_* and background offsets *b_j_* for each of the *N* data sets across all *M* data points,

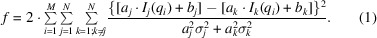

The *datcombine* tool also allows the application of filters for outlier data points and/or data with statistical errors that only serve to increase the noise in the final consensus scattering profile. Depending on the sample concentration and the various instrument configurations and instrument parameters (such as detector size and distance, exposure time and the incoming number of photons) the uncertainty of each data point [*I_j_* (*q_i_*)] will vary, a fact that is reflected in the magnitude of the error estimate. The total error estimate of *M* averaged data points with respective error estimates σ_*i*_, *i* = 1, …, *M*, propagates as 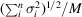 . Using *M*+1 data points should reduce the total propagated error estimate, that is

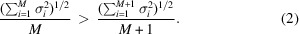

Therefore, any given *I_j_* (*q_i_*) that comes with such high uncertainty σ_*i*_ that it would increase the propagated error of the average can be excluded. The calculation is independent of the actual intensity value and assumes that the errors are correctly propagated. Statistical error estimates for the contributed data were validated by comparing all pairwise solvent measurements.

The program first sorts the data at each *q_i_* value by the magnitude of the errors, starting from the smallest, and proceeds to add data with errors of increasing magnitude that do not increase the propagated average. We note here that there are alternatives to using the average error to exclude high-uncertainty data, for example by using a maximum-likelihood error estimate and an error-weighting scheme. Resolution of the optimal approach is complicated, however, by the facts that the data have been re-gridded and experimental errors in neighbouring *q* channels are correlated to some extent. That said, the differences in the magnitude of propagated errors for the plain average or the maximum-likelihood approaches are expected to be small, and for the purposes here we chose to implement *datcombine* using the subroutines within the *ATSAS* program package, which have been thoroughly tested over a long period of time.

Data points also can be excluded by the identification of outliers, defined here as intensity values that are not very likely to have been drawn from the expected normal distribution of intensities at any given *q_i_* value. To detect such outliers, the modified *Z*-score of Iglewicz & Hoaglin (1993 ▸) is employed, 

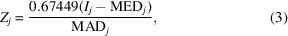

where MED_*j*_ is the median of the intensities at any *q_i_* value and MAD_*j*_ is the corresponding median absolute deviation, a variation estimate for the median. In data processing, any intensity value [*I_j_* (*q_i_*)] where *Z_j_* > 2 is removed. As the definition of an outlier depends on the current scaling, and any potential outlier was used to calculate the scaling coefficients, scaling and constant adjustment are then recalculated with the identified outliers removed. The whole process is repeated until no more outliers are removed after scaling and adjusting the data.

A user manual for *datcombine* is available online (https://www.embl-hamburg.de/biosaxs/manuals/datcombine.html).

### 3.4. Modelling

There are several approaches to calculating SAS scattering profiles from atomic coordinates, and it is not within the scope of this study to review or evaluate all of the different approaches taken. Further, the consensus data from this study by themselves provide no basis for concluding that any one method is preferred. Rather, the aim here is to provide a set of data that could be used to improve any given approach. Nevertheless, it was relevant to see how well the consensus profiles compared with prediction, and so from the readily available methods we considered examples that took different approaches to modelling the hydration layer and its contribution to the scattering. *WAXSiS* (Chen & Hub, 2014 ▸; Knight & Hub, 2015 ▸) uses explicit-solvent all-atom molecular-dynamics (MD) simulations to account for the hydration layer and excluded solvent, and when fitting to an experimental curve an optional additive constant can be applied. *CRYSOL* (Svergun *et al.*, 1995 ▸), *CRYSON* (Svergun *et al.*, 1998 ▸), *Pepsi-SAXS*/*Pepsi-SANS* (Grudinin *et al.*, 2017 ▸) and *FoXS* (Schneidman-Duhovny *et al.*, 2016 ▸) represent the hydration layer as a shell of uniform contrast surrounding the atomic structure. When optimizing the fit to experimental data, free parameters are refined to account for the excluded volume of bulk solvent by the protein and for the contrast of the hydration shell, and there is an optional constant subtraction to account for errors in background subtraction. The modelling presented here used *CRYSOL* and *CRYSON* as implemented in *ATSAS* online (version 3.1.0; https://www.embl-hamburg.de/biosaxs/atsas-online/), *Pepsi-SAXS* run locally (Linux version 3.0), *Pepsi-SANS* as implemented on the ILL *Pepsi* home site (https://pepsi.app.ill.fr/) and *FoXS* (version 2.16.0) via the *FoXS* website (https://modbase.compbio.ucsf.edu/foxs/). To improve the convergence of the predicted SAS profiles, custom *WAXSiS*-type calculations for SAXS and SANS, incorporating either X-ray form factors or neutron scattering factors, were performed locally using *GROMACS* (Abraham *et al.*, 2015 ▸) and were run for longer times compared with the version available on the *WAXSiS* website (http://waxsis.uni-goettingen.de/) that uses *YASARA* (Krieger & Vriend, 2015 ▸; Section S1 has full details of the MD simulation systems, which are openly shared at Zenodo at https://doi.org/10.5281/zenodo.7057567).

The atomic coordinates of crystal structures deposited in the Protein Data Bank (PDB) with accession codes 7rsa (Wlodawer *et al.*, 1988 ▸), 2vb1 (Wang *et al.*, 2007 ▸), 2dfc (Watanabe *et al.*, 2006 ▸), 3l8w (Gabison *et al.*, 2010 ▸) and 1mnz (E. Nowak, S. Panjikar & P. A. Tucker, unpublished work) were used to calculate SAS profiles for RNaseA, lysozyme, xylanase, urate oxidase and xylose isomerase, respectively. In addition, the NMR solution structure of RNaseA in the RECOORD database (https://www.ebi.ac.uk/pdbe/recalculated-NMR-data), which is a 32-model ensemble (PDB entry 2aas; Santoro *et al.*, 1993 ▸), was considered. For xylose isomerase, a single N-terminal Met missing from the crystal structure was added to the coordinate file using *PyMOL* (version 2.3.3; Schrödinger). For urate oxidase the crystal structure with PDB entry 3l8w contains the inhibitor xanthine, whereas the inhibitor in the SAS samples was 8-azaxanthine, which differs from xanthine by just one atom (a C to N substitution, 1 Da molecular mass) and binds in the same way. PDB entry 3l8w was chosen for its superior resolution (1.0 Å) compared with PDB entry 1r51 (1.75 Å), which does have the 8-azaxanthine inhibitor, but comparison of the two structures in *PyMOL* gives r.m.s.d. values of 0.26 Å over one chain and 0.326 Å over all four chains and indistinguishable predicted scattering patterns were obtained using *CRYSOL*. Both PDB entries 3l8w and 1r51 have six amino acids missing from the C-terminus (SLKSKL). A PDB file was thus prepared starting with PDB entry 3l8w and completing the C-terminus with the six missing residues using *ModLoop* (Fiser & Sali, 2003 ▸). For all proteins, additional ions or ligands that were present in the coordinate files but not present in the solution conditions were removed.

### 3.5. SAXS results

#### 3.5.1. Preliminary evaluations

Histograms of the derived structural parameters (Supplementary Figs. S3 and S4) and the corresponding *R*_g_ averages and ranges for the batch and SEC–SAXS data (Table 2 and Supplementary Table S5) include data from all instruments and show clustering of values with varying degrees of spread for different proteins. These data include SAXS measurements that were made for urate oxidase and xylose isomerase in H_2_O and D_2_O, as D_2_O had no discernible impact on the SAXS profile for these two proteins (Supplementary Table S6).

Of the five proteins measured, xylose isomerase stands out as having the tightest distribution of derived structural parameters, with no significant variation in mean values between batch and SEC–SAXS data. Importantly, xylose isomerase consistently showed significant interparticle interference effects at low *q* values for samples measured at concentrations of >1 mg ml^−1^ and it was necessary to carefully examine and re-reduce a significant portion of the submitted SEC–SAXS data to exclude measurement frames in which the concentration exceeded this value.

Three sets of batch-only SAXS data submitted for RNaseA showed severe aggregation at all concentrations measured and were therefore not included in further analysis. The remaining sets gave a cluster of structural parameters, with an ∼0.5 Å increase in mean *R*_g_ values and a 2–3 Å increase in *d*_max_ for the batch data compared with SEC–SAXS. These increases are potentially attributable to the sensitivity of the small RNaseA protein to radiation-induced aggregation and/or to a small degree of concentration or time-dependent aggregation.

Compared with xylose isomerase, the SEC–SAXS results for urate oxidase show a broader distribution of Guinier *R*_g_ values that is significantly reduced in the *P*(*r*)-derived *R*_g_ values, indicating that the overall profile shape is consistent but with small variations at very low *q* values, which are likely to be due to a small degree of sample heterogeneity for this sample. This interpretation is consistent with the observation that the batch data for urate oxidase give somewhat larger structural parameters on average (by ∼0.7 Å in *R*_g_ and ∼15 Å in *d*_max_) compared with SEC–SAXS measurements. Due to the timing of sample availability and SAS instrument availability some measurements for this protein were delayed by up to 6–7 months from shipment.

Xylanase shows the largest mean shift in *R*_g_ and *d*_max_ values between SEC–SAXS and batch SAXS measurements, with all of the batch and half of the SEC–SAXS measurements yielding *P*(*r*) profiles with prominent, albeit relatively small, positive values at *r* values of >50 Å. Further, multi-angle laser light scattering (MALLS) data measured at the European Molecular Biology Laboratory (EMBL) Hamburg and BioCAT at the Advanced Photon Source (APS) (data not shown) indicated the presence of dimers. It thus appears that the majority of the SAXS data show some degree of persistent xylanase dimers. Only four of the SEC–SAXS profiles gave Guinier plots and *P*(*r*) distributions that had characteristics consistent with monomeric xylanase, that is linear Guinier regions and well behaved bell-shaped *P*(*r*) functions with the expected *R*_g_ and *d*_max_ values based on the crystal structure monomer, and when *P*(*r*) was calculated with *d*_max_ = 100 Å the profiles were essentially zero, within error, from 50 to 100 Å. It therefore was decided to continue analysis with just these four SEC–SAXS measurements.

Lysozyme also showed significant variability both within and between measurement classes. The batch SAXS results show two clusters of *P*(*r*)-derived *R*_g_ values centred at ∼14.5 Å and at ∼15.4 Å, while the corresponding SEC–SAXS values have a predominant cluster of *R*_g_ values around 15 Å and an outlier near 14 Å. Notably, two of the SEC–SAXS measurements even gave *P*(*r*) profiles that indicated the presence of unresolved aggregate with uncharacteristically large *R*_g_ values, potentially due to lysozyme having a heightened sensitivity to radiation damage.

On average, the spread of *R*_g_ values for the batch measurements is greater than that observed for the SEC–SAXS data, with the average spread and standard deviations for the batch measurements being approximately two times larger compared with SEC–SAXS measurements (Supplementary Table S5).

#### 3.5.2. Optimizing *I*(*q*) versus *q* and obtaining a consensus SAXS data set

To obtain the optimal scattering profiles over the widest *q*-range possible, SEC–SAXS data were merged with batch SAXS data. This merging procedure offers the opportunity to eliminate the influence at low *q* values of small amounts of potential contaminating aggregates or interparticle interference in the batch data, while batch data collected at higher concentrations and for longer times provide improved statistics at higher *q* values that are unaffected by small amounts of aggregate or interparticle interference. Similarly, some merged data sets were constructed using all batch data by combining lower concentration data with higher concentration data. The merging protocol used *primusQt* from the *ATSAS* suite (versions 3.0.0, 3.0.1 or 3.1.0) and was performed centrally (by JT and PV) to ensure a consistency of approach. Starting with an overlap region (typically ∼50–100 data points) in the mid-*q* regime, *I*(*q*) profiles were placed on a common scale to prepare for merging the lower *q* region of a SEC–SAXS or lower concentration batch measurement with the higher *q* region of a higher concentration batch measurement. An iterative process was used to test for any influence of potential aggregation or interparticle interference from the higher concentration batch data on the merged data by systematically increasing the minimum *q* value accepted from the batch data and repeating the *P*(*r*) calculation for the resulting merged *I*(*q*) profile and comparing with that obtained from the SEC–SAXS or lower concentration batch data as applicable. Once it was established that the data from the higher concentration measurement did not alter the *P*(*r*) shape or derived structural parameters, the overlap region was minimized to still enable accurate scaling of the two data sets while eliminating un­necessary data with large experimental uncertainties. Generally, the highest concentration batch measurement was used in the merge with either SEC–SAXS data or a lower concentration batch measurement assessed to be free of interparticle correlations or aggregation. In some cases, a three-way merge gave the optimal result, for example with SEC–SAXS and two batch SAXS measurements at different concentrations.

Due to the high degree of variability in the lysozyme results, data were selected for inclusion in the calculation of a consensus set from either pure SEC–SAXS data or merged data that gave well behaved *P*(*r*) transforms. That is, the expected bell-shaped profile is observed with no negative dip or additional positive features upon extending *r* beyond the putative *d*_max_ that would indicate interparticle interference or aggregation, respectively. There were ten lysozyme scattering profiles that met these criteria, and they had *P*(*r*)-derived *R*_g_ values ranging from 14.2 to 15.2 Å, which is significantly greater than the expected variation given the statistical precision to which the data were measured.

**Figure 2:**
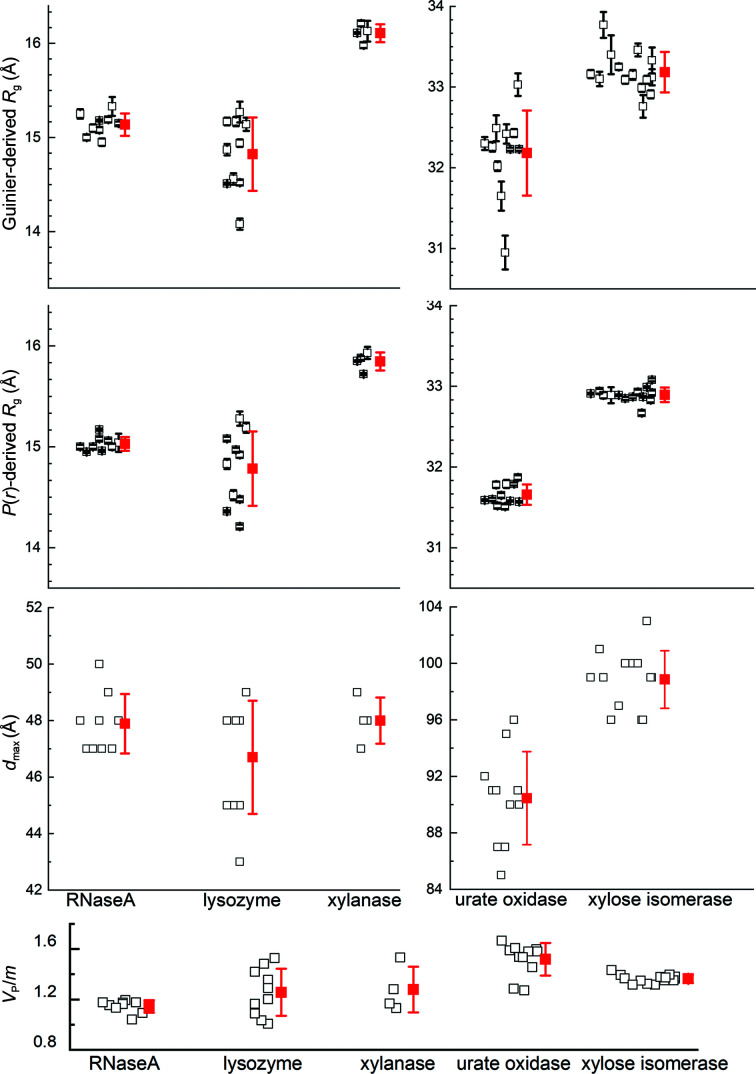
Distribution of Guinier and *P*(*r*)-derived *R*_g_ values, *d*_max_ values and the Porod volume to molecular mass ratio (*V*_P_/*m*) for the data contributing to the consensus SAXS profiles for each protein. Individual experimental values are represented as black open squares, with horizontal offsets for clarity and error bars for *R*_g_ values (standard errors). Red squares represent the mean values for each set, with error bars indicating ±1 standard deviation.

After the above evaluations, nine, ten, four, 11 and 14 independently measured scattering profiles consisting of a mixture of pure SEC–SAXS, pure batch SAXS and merged SEC–SAXS/batch SAXS data were selected to calculate potential consensus profiles for RNaseA, lysozyme, xylanase, urate oxidase and xylose isomerase, respectively (see Supplementary Table S4*b* for the exact make-up). The Guinier- and *P*(*r*)-derived *R*_g_ values, *d*_max_ values and Porod volume/molecular mass (*V*_P_/*m*) ratios for these data sets are clustered largely as expected (Fig. 2 ▸). RNaseA, xylanase and xylose isomerase show relatively tight clustering, while lysozyme and the Guinier *R*_g_ values for urate oxidase show a greater spread than expected based on the statistical precision to which the data were measured. Indeed, the SAXS data for xylose isomerase proved to be the most robust among all of the proteins, with the largest number of contributing profiles, including SAXS measurements in H_2_O and D_2_O. All facilities contributed data for multiple proteins that were included in the final data sets for generating consensus profiles, which for each protein (excepting xylanase) used data from eight or more of the 12 participating SAXS facilities.

Examination of the background levels for the buffer-subtracted SAXS data collected on different instruments showed some variability after scaling. RNaseA and xylose isomerase had sufficient statistical precision to observe that most of the data in the mid-to-high-*q* region lay in a relatively narrow band, but with some outliers (Supplementary Fig. S5). For RNaseA and xylose isomerase, the band width is ∼0.2% and ∼0.4% of *I*(0), respectively (sampled at *q* ≃ 0.5 Å^−1^). Assuming that the accurate background level is included within these bands, this corresponds to an uncertainty in the background scattering level of approximately ±8% for RNaseA and ±20% for xylose isomerase. Notably, the outliers are likely to reflect issues with solvent blank preparation for individual proteins as they were not consistently observed for the five proteins measured on any one instrument.

**Figure 3:**
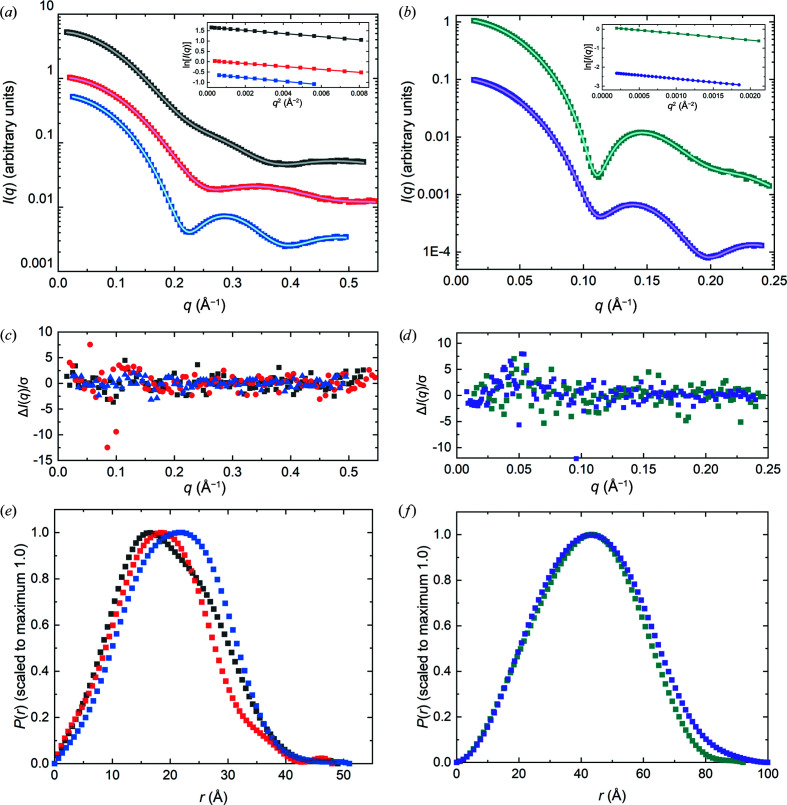
(*a*, *b*) *I*(*q*) versus *q* for consensus SAXS data (symbols) for each protein with *P*(*r*) model fits (lines). Insets are Guinier plots to *qR*_g_ = 1.3. (*c*, *d*) Error-weighted residual plots for the *P*(*r*) model fits in (*a*) and (*b*), respectively. (*e*, *f*) *P*(*r*) versus *r* corresponding to the fits in (*a*) and (*b*), respectively. Error bars are standard errors, and where not apparent are smaller than the symbols. The colour key for the symbols throughout is RNaseA, black; lysozyme, red; xylanase, blue; urate oxidase, dark cyan; xylose isomerase, purple.

To minimize the influence of parasitic scattering or small degrees of sample heterogeneity in calculating the consensus profile, a low-*q* limit was set for each SAXS profile at the value selected for Guinier analysis by *autoRg*. Calculations using *datcombine* were made with filters disabled, with outlier-only and error-only filters and with both outlier and error filters, each of which yielded essentially the same scattering profile, differing only in the error distribution resulting from the exclusion of different data with different filtering options. The general agreement among the set of SAXS profiles obtained for each protein is demonstrated by the superposition of the individual profiles with each other (after scaling and constant adjustment) and with the consensus profiles from *datcombine* (Supplementary Fig. S6 and S7). Notably, the structural parameters reported for the SEC–SAXS data and consensus profiles are in excellent agreement (Table 2 ▸). Further, the average *V*_P_/*m* values for the consensus profiles are all in the range 1.29–1.61, which compares favourably with estimated values based on calculated partial specific volumes and hydration for each protein sequence (Section S2, Supplementary Table S1). The *P*(*r*) model fits to the consensus profiles (Fig. 3 ▸) give the expected *P*(*r*) profile, with error-weighted difference distributions largely having the expected random distribution of points about a good model fit, ideally ±3 with standard errors dominated by propagated counting statistics. Here, we show the outlier- and error-filtered results for all proteins except xylose isomerase, where only the outlier filter was applied. For xylose isomerase, the outlier plus error-filter result gives an unrealistically small error distribution in the mid-*q* region as assessed by the error-weighted difference plot, likely due to the dominance of one or a few high statistical precision measurements in certain regions. The Guinier plots (insets in Figs. 3 ▸ *a* and 3 ▸ *b*) are all linear with Pearson correlation coefficients >0.999.

### 3.6. SANS results

#### 3.6.1. Preliminary evaluations

**Figure 4:**
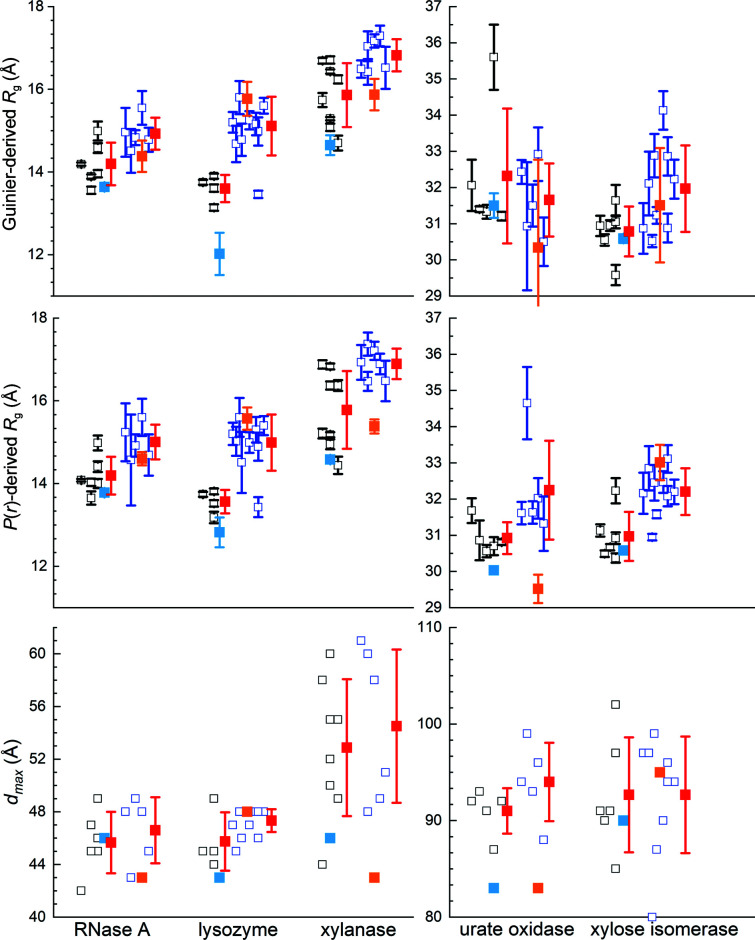
Distribution of Guinier- and *P*(*r*)-derived *R*_g_ values and associated *d*_max_ values for SANS measurements for each protein in D_2_O (batch data, open black squares; SEC–SANS data, light blue filled squares) and H_2_O (batch data, open blue squares; SEC–SANS data, orange filled squares) with horizontal offsets for clarity. Errors bars on individual *R*_g_ values are standard errors. Red squares represent the mean value for each set, with the error bar indicating ±1 standard deviation. The values for H_2_O urate oxidase SEC–SANS are for data that have a constant additive background adjustment to match the batch data.

While SANS has the unique advantage of using deuterium substitution to achieve contrast variation in studies of complex, multi-component systems (for recent reviews, see Mahieu & Gabel, 2018 ▸; Trewhella, 2022 ▸; Krueger, 2022 ▸), neutron sources are orders of magnitude less intense than synchrotron sources. For example, the flux on the sample for the high-intensity D22 SANS instrument at the ILL is comparable to that achieved with benchtop X-ray sources. As a result, counting statistical errors in SANS measurements are much greater than for SAXS. Additionally, the incoherent scattering cross-section from hydrogen (^1^H) is orders of magnitude greater than the coherent neutron scattering cross-sections of nuclei in a typical biological sample, resulting in a background of isotropic scattering. This incoherent contribution introduces noise that is especially significant for measurements in H_2_O and with increasing *q* values given the rapid decay of the coherent scattering contribution away from zero angle. There are also far fewer neutron scattering facilities worldwide and traditionally SANS instruments have generally been viewed as having insufficient intensity to support SEC–SANS, although it has been developed at the high-intensity D22 instrument at the ILL (Johansen *et al.*, 2018 ▸; Jordan *et al.*, 2016 ▸). Thus, just one set of SEC–SANS data was collected in H_2_O and in D_2_O for all five proteins and fewer batch data sets per protein compared with the SAXS measurements (Supplementary Table S4*a* ). Because neutron radiation is non-ionizing and thus nondamaging to biomolecules, no measurements had to be excluded due to radiation-induced aggregation, although D_2_O-induced aggregation proved sufficiently severe in one high-concentration lysozyme measurement that it was excluded. Also, one set of submitted xylanase measurements (two each for measurements in H_2_O and D_2_O buffer) had anomalously high backgrounds and was not used. Otherwise, all of the contributed SANS data (Supplementary Table S4*c* ) were used in the final analyses. From these data, the Guinier- and *P*(*r*)-derived *R*_g_ values and *d*_max_ values for the SANS data sets for each protein in H_2_O and D_2_O show the expected clustering. Further, the expected decrease in the average structural parameters for SANS measurements in D_2_O compared with H_2_O, a consequence of the differences in hydration-layer contrast, is observed (Fig. 4 ▸, Table 3 and Supplementary Table S7).

#### 3.6.2. Optimizing *I*(*q*) versus *q* and the consensus SANS data sets

Merging data acquired using SEC–SANS and batch data for an optimal SANS profile proved to be beneficial for RNaseA and lysozyme in D_2_O and for xylanase in D_2_O and H_2_O. Given the relatively poorer statistics inherent to the SANS data and the fact that there is only one SEC–SANS measurement per protein, these merges were performed with the consensus batch SANS data using the same procedure as for the SAXS data merges. Except where noted, the consensus results reported here are for the *datcombine* results with both outlier and error filters applied.

The SEC–SANS *R*_g_ values for RNaseA in D_2_O were on average significantly smaller than the mean for the batch measurements (Table 3 ▸), suggesting the presence of a small amount of aggregate in at least some of the batch data. It was therefore meaningful to merge the SEC–SANS D_2_O data with the consensus batch result calculated using all six batch measurements in D_2_O. The larger errors for RNaseA meant there was no significant difference between the consensus batch result and the SEC–SANS data; therefore, all five batch profiles plus the SEC–SANS profile were combined.

Of the five lysozyme batch data in D_2_O, four gave *R*_g_ values in the range 13–14 Å, with one value of >15 Å that clearly had a large amount of aggregate and was therefore excluded from further analysis. The SEC–SANS data gave a Guinier *R*_g_ value of 12.16 ± 0.42 Å, which agrees with the *CRYSON*-predicted value for lysozyme in D_2_O based on the crystal structure (Supplementary Table S8). The SEC–SANS data for lysozyme (and for xylanase) in D_2_O had a significantly greater *q*_min_ (0.04 Å^−1^) and Δ*q* (0.0055 Å^−1^) compared with the other SEC–SANS data sets, making it more challenging to identify a good merge region for combining with batch data, but a satisfactory merge was made between the SEC–SANS profile and the consensus result from the four batch profiles. For lysozyme in H_2_O, the SEC–SANS and batch *R*_g_ values were the same within the errors and thus the SEC–SANS and all batch measurements were simply combined to yield the consensus profile.

Xylanase SEC–SANS data for measurements in D_2_O and H_2_O were consistent with scattering predominantly from the monomer form based on *R*_g_ and *d*_max_ values. In contrast, all of the batch data gave *P*(*r*) profiles indicating the presence of varying amounts of dimer. The SEC–SANS data were therefore merged with the consensus batch data. For the H_2_O result only the outlier filter was applied as the error filter consistently gave negative values at high *q*.

For urate oxidase, all batch plus SEC–SANS data were combined for measurements in D_2_O as there was no significant difference in Guinier *R*_g_ for SEC–SANS compared with the result with all batch data. For urate oxidase in H_2_O, the SEC–SANS data had an unusually high background and gave a Guinier *R*_g_ value that was unrealistically small. Upon scaling and adjusting to the batch data, the Guinier *R*_g_ value came within the error of the predicted value, but with very large errors. In the end, all batch plus SEC–SANS data for each of the D_2_O and H_2_O sets of measurements were included in calculating the consensus profiles.

Xylose isomerase in D_2_O and H_2_O gave the same Guinier *R*_g_ values, within error, for the SEC–SANS and all batch consensus data. Attempts to combine the SEC–SANS and batch data for measurements in D_2_O were complicated by their very different background levels and the fact that the SEC–SANS data were collected at twice the concentration of the highest concentration batch data. The superior statistics of the SEC–SANS data were such that they overwhelmingly dominated the result when included in *datcombine* with the error filter on. Using the outlier-only filter with all batch plus SEC–SANS data or both the error and outlier filters with batch-only data gave very similar results in terms of the profile shape, but with improved statistics for the latter, which is what is presented here. For the data in H_2_O the SEC–SANS data had sufficiently poor statistics that they did not survive the error filter. However, there were two sets of batch measurements made at 6.8 mg ml^−1^ protein concentration where the low-*q* regime showed significant interparticle interference. We therefore used the same merge process as for the SEC–SAXS batch data merges, but in this case merging the consensus batch result (with outlier and error filters applied) for the lower concentration data with the high-*q* regime of the two 6.8 mg ml^−1^ measurements.

In summary, for RNaseA, lysozyme and xylanase in D_2_O and for xylanase in H_2_O, SEC–SANS data were merged with consensus results from batch data to remove the influence of small amounts of aggregate, or in the case of xylanase likely dimer. For RNaseA and lysozyme in H_2_O, and urate oxidase in H_2_O or D_2_O, batch and SEC–SANS data showed no significant differences and were simply combined. In the case of xylose isomerase, batch data measured for samples <2 mg ml^−1^ were combined, and in the case of measurements in H_2_O were merged with higher concentration data (6.8 mg ml^−1^ for *q* > 0.04 Å^−1^) to improve the high-*q* statistics. Like the SAXS results, there is general agreement among the set of scattering profiles combined for each protein in H_2_O and D_2_O, as demonstrated by the superposition of the individual SANS profiles from *datcombine* with filters disabled. There is also good superposition of the *datcombine* outputs (Supplementary Figs. S8 and S9).

The structural parameters reported for the SEC–SANS data and the consensus SANS profiles (Table 3 ▸) are in good agreement, except for lysozyme and urate oxidase in H_2_O, each of which had issues with the SEC–SANS measurement, as noted above. Of the SEC–SANS/batch SANS merged profiles, RNaseA in D_2_O and xylanase in D_2_O and H_2_O each show excellent agreement with the SEC–SANS data over the entire *q*-range (Supplementary Fig. S10), with χ^2^ values in the range 0.38–1.1 and *CorMAP*-adjusted *P* values in the range 0.5–0.44. The comparison for lysozyme in D_2_O is not as good, with a noticeable deviation around 0.2 Å^−1^ reflected in the somewhat larger χ^2^ = 1.3. Attempts to improve this comparison were unsuccessful and suggest that the result is due to difficulties in merging these data, where the useful overlap region was limited by the experimental *q*-ranges and the fact that the SEC–SANS and batch data had very different backgrounds. It also may be the case that some influence from aggregates in the batch data was not fully removed.

**Figure 5:**
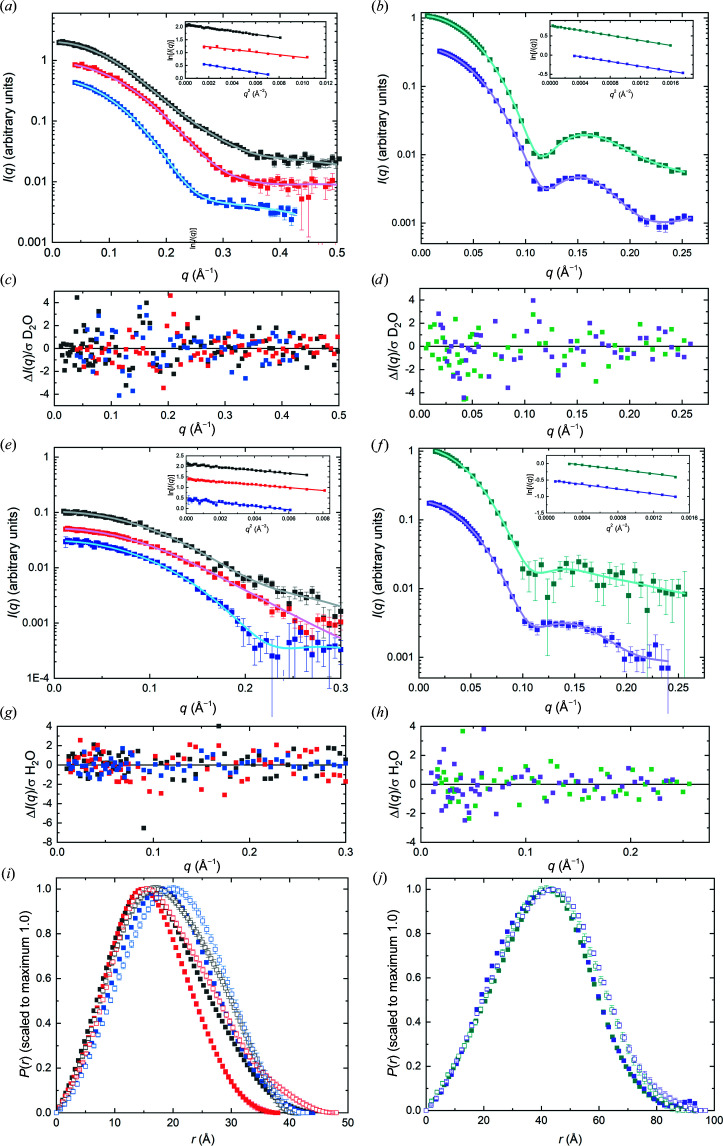
(*a*, *b*) *I*(*q*) versus *q* (symbols) with *P*(*r*) model fits (black lines) from consensus SANS data in D_2_O for each protein, with Guinier plots (to *qR*_g_ = 1.3) as insets. (*c*, *d*) Error-weighted residual plots for the fits in (*a*) and (*b*), respectively. (*e*) and (*f*) are the same plots as in (*a*) and (*b*) but for SANS data in H_2_O, with (*g*) and (*h*) showing the corresponding error-weighted residual plots. (*i*, *j*) Corresponding *P*(*r*) versus *r* plots for the fits in (*a*) and (*b*) (D_2_O, solid squares) and (*e*) and (*f*) (H_2_O, open squares). The colour key throughout is RNaseA, black; lysozyme, red; xylanase, blue; urate oxidase, dark cyan; xylose isomerase, purple. Error bars are standard errors, and where not apparent are smaller than the symbols.

The *P*(*r*) model fits to the combined data (Fig. 5 ▸) give the expected bell-shaped *P*(*r*) versus *r* profile, with error-weighted difference distributions largely having the expected ±3 standard deviations. Guinier plots are all linear with Pearson correlation coefficient values >0.99 except for lysozyme in D_2_O (0.97) and xylanase in H_2_O (0.98); for urate oxidase and xylose isomerase in D_2_O they were >0.999. The *P*(*r*) versus *r* plots for each protein in H_2_O and D_2_O show the expected shift to smaller *r* values due to the decreasing impact of the hydration layer in D_2_O compared with H_2_O and this trend is reflected in the *R*_g_ values from the consensus profiles (Table 3 ▸).

## 4. Comparisons with prediction

**Figure 6:**
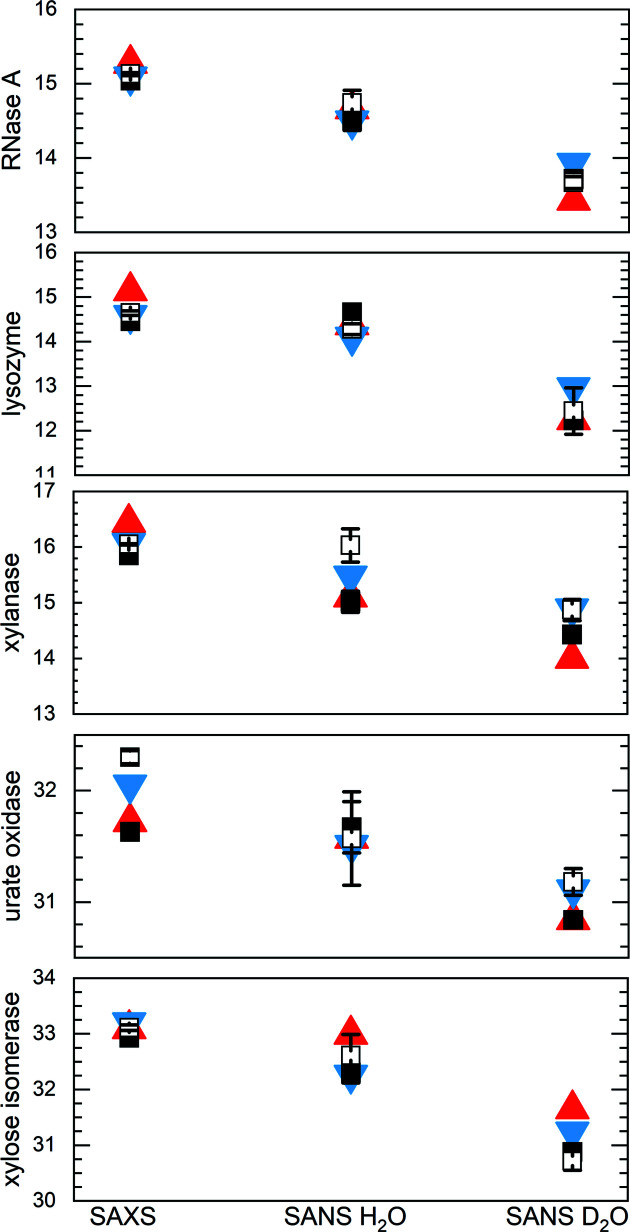
Guinier- and *P*(*r*)-derived *R*_g_ values (open and filled black squares, respectively) for RNaseA, lysozyme, xylanase, urate oxidase and xylose isomerase consensus profiles for SAXS, SANS in H_2_O and SANS in D_2_O measurements (Tables 2 ▸ and 3 ▸) compared with *R*_g_ values predicted with *CRYSOL*/*CRYSON* (red filled triangles) and *WAXSiS* (blue filled inverted triangles) (Supplementary Table S8). Where they are not evident, error bars for *R*_g_ from the consensus curve are smaller than the symbols.

A preliminary assessment of the agreement between experiment and prediction based on the crystal structures described above (in Section 3.4) used *R*_g_ values from Guinier fits of the *WAXSiS* predictions, as well as the *R*_g_ and *d*_max_ values output by *CRYSOL* (Svergun *et al.*, 1995 ▸) and *CRYSON* (Svergun *et al.*, 1998 ▸) (implemented in *ATSAS* online 3.1 with default parameters and without any fitting to experimental data) (Supplementary Table S8). When compared with experiment, the experimental *R*_g_ values for each protein show a decrease from SAXS to SANS in H_2_O to SANS in D_2_O measurements that is generally consistent with the predictions, with SANS measurement of xylanase in H_2_O and xylose isomerase in D_2_O falling just outside the predicted values (Fig. 6 ▸). Shape-restoration calculations using *DAMMIN* (Svergun, 1999 ▸; as implemented in *ATSAS* online 3.0) yielded total excluded volumes of 18 380, 15 973, 26 800, 236 005 and 268 299 Å^−3^ for RNaseA, lysozyme, xylanase, urate oxidase and xylose isomerase, respectively. Taking the calculated volume/mass ratios from Supplementary Table S1, these volumes give estimates of the molecular masses that are 91%, 77%, 90%, 113% and 102%, respectively, of the values expected from the chemical composition. For urate oxidase, the 13% excess volume may be associated with the existence of a large central water-filled quasi-cylindrical channel in this protein (see Fig. 1 ▸). The somewhat lower excluded volume value for lysozyme (23% lower than expectation compared with 2–13% for all the other proteins) suggests the influence of a small amount of interparticle interference in the consensus profile.

**Figure 7:**
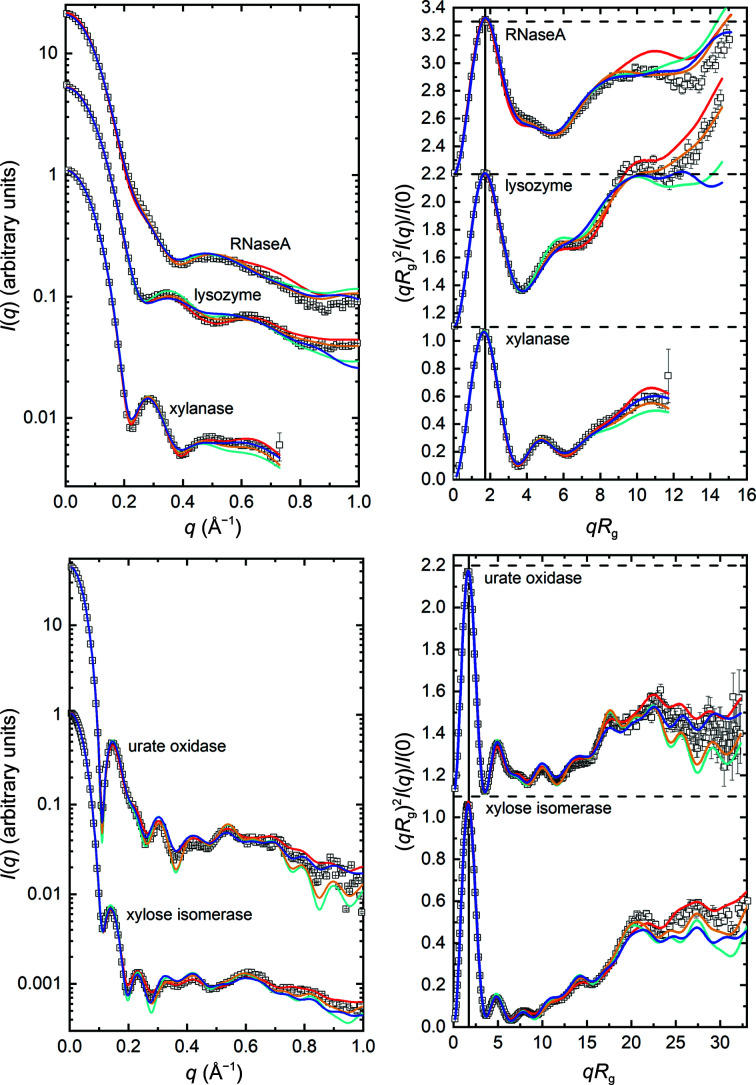
*I*(*q*) versus *q* and dimensionless Kratky [(*qR*_g_)^2^ *I*(*q*)/*I*(0) versus *qR*_g_] plots for the consensus data (open black squares) overlaid with the profiles predicted by *WAXSiS* (red), *CRYSOL* (cyan), *Pepsi-SAXS* (orange) and *FoXS* (blue) from the crystal structures. Every second or third experiment point is omitted for clarity. *I*(*q*) versus *q* plots are offset vertically, while the Kratky plots are stacked vertically so that for each panel the dashed lines are for (*qR*_g_)^2^ *I*(*q*)/*I*(0) = 0.0 or 1.1 for the plots above or below, respectively. The solid black reference lines in the Kratky plots are at *qR*_g_ = 1.73. Error bars are standard errors based on propagated counting statistics.

Beyond the parameter predictions, multiple methods predicted the main features of the SAXS profiles. As examples, we show the results from *WAXSiS*, *CRYSOL*, *Pepsi-SAXS* and *FoXS* calculations using atomic coordinates for the crystal structures described in Section 3.4 (Fig. 7 ▸). The dimensionless Kratky plots are the most useful in evaluating the fits at mid-to-high *q* values (>0.2 Å^−1^), and these also nicely show the expected bell shape with a maximum magnitude of ∼1.1 at *qR*_g_ ≃ 1.73 for globular, essentially isometric particles (Durand *et al.*, 2010 ▸). The *WAXSiS* results required scaling and an additive constant (using *primusQt*) to avoid divergence of the scattering profile at mid-to-high *q* values as might be expected given the variability in background levels observed in the original experimental data. While qualitatively there is good agreement between the predicted and consensus profiles, error-weighted residual intensity plots (Supplementary Fig. S11*a* ) reveal differences. There is a broad oscillation in the difference plot for RNaseA spanning the *q* region 0–0.4 Å^−1^, while the urate oxidase and xylose isomerase difference plots show much sharper oscillating features. The latter features are due to small differences in the amplitudes and positions of the maxima and minima arising from the approximately spherical nature of the scatterers. These differences are especially amplified in the residual plots for the consensus SAXS data because the propagated statistical errors are exceptionally small, which is also reflected in the magnitude of the reduced χ^2^ values (Supplementary Table S9). For the SAXS data that have the smallest errors, the χ^2^ values average 26.5 for all but RNaseA, which consistently gave the largest values for all methods, with an average of 79.6. The χ^2^ values are smallest for SANS in H_2_O (2.1), which has the largest measurement errors, and intermediate for SANS in D_2_O (12.5). These trends demonstrate one of the limitations of χ^2^ as a measure of quality of fit for any model as it is the magnitude of the data that determines the magnitude of χ^2^.

It is noteworthy that the χ^2^ values for the RNaseA SAXS data were consistently larger than those for the other proteins. Further, the consistent and distinctive broad oscillation in the RNaseA residual plot is characteristic of differences in the spacing of domains, or potentially some sort of oligomerization. The latter was judged to be unlikely after re-examination of selected SEC–SAXS data sets, which did not show evidence for either oligomers in the main elution peak or a monomer–dimer equilibrium. As an NMR solution structure is available for RNaseA, some preliminary calculations were performed using the conformers in the NMR structure (PDB entry 2aas; Santoro *et al.*, 1993 ▸) in the RECOORD database. All 32 scattering curves from the ensemble were calculated using *WAXSiS* as implemented on the web server (Knight & Hub, 2015 ▸). A linear combination of those curves that best fit the experimental curve was found using an NNLS tool in *US-SOMO* (Brookes *et al.*, 2016 ▸) and showed a significantly improved fit with two conformations of the initial 32 (conformers 3 and 7 in proportions of 0.73 and 0.27, respectively; not shown). These results suggest that the conformation of RNaseA may be constrained by crystal-packing forces and that further exploration is required to understand its solution state, with the solution NMR conformations providing one possible avenue to explore.

**Figure 8:**
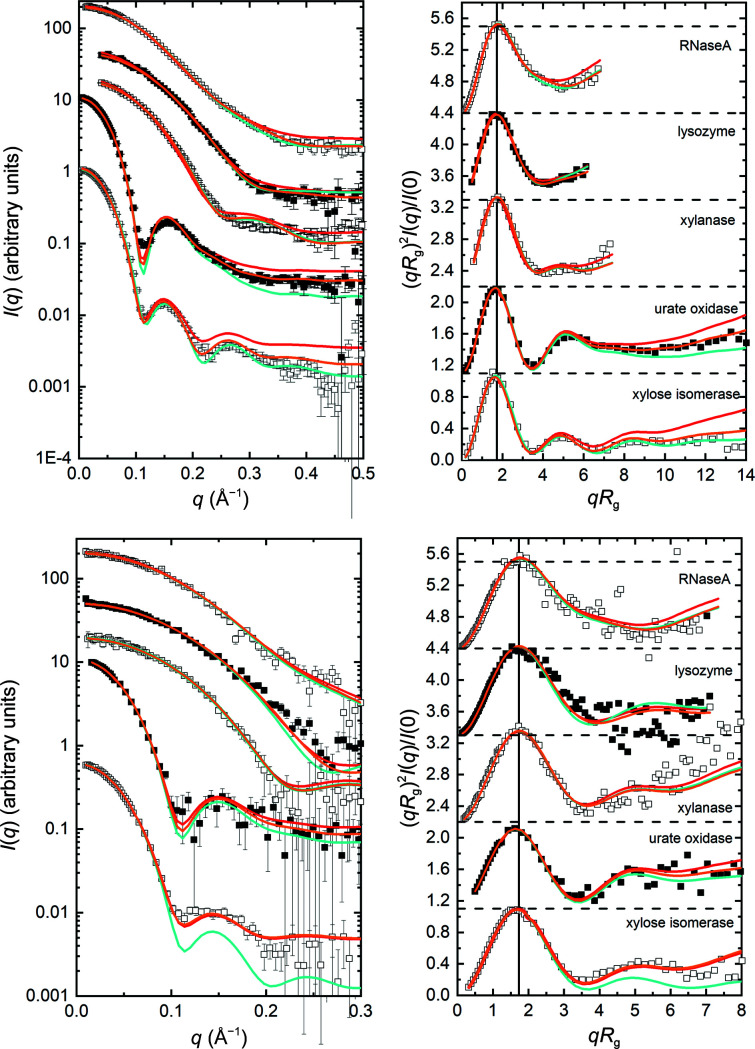
*I*(*q*) versus *q* and dimensionless Kratky [(*qR*_g_)^2^ *I*(*q*)/*I*(0) versus *qR*_g_] plots for the consensus SANS data (black squares) in D_2_O (upper plots) or H_2_O (lower plots) overlaid with the profiles predicted by *WAXSiS* (red), *CRYSON* (cyan) and *Pepsi-SANS* (orange) from the crystal structures. *I*(*q*) versus *q* plots are offset vertically for clarity; from top to bottom, RNaseA, lysozyme, xylanase, urate oxidase and xylose isomerase. Kratky plots are stacked vertically so that for each panel the dashed lines are for (*qR*_g_)^2^ *I*(*q*)/*I*(0) = 0.0 or 1.1 for the plots above or below, respectively. Black solid reference lines in the Kratky plots are at *qR*_g_ = 1.73. Error bars are standard errors based on propagated counting statistics.

*WAXSiS*, *CRYSON* and *Pepsi-SANS* predictions based on crystal structure coordinates for RNaseA, lysozyme, xylanase, urate oxidase and xylose isomerase (Fig. 8 ▸) also show good qualitative agreement with the consensus profiles, although the statistical quality of the data restricts the useful comparisons to *q* <0.5 Å^−1^ for SANS in D_2_O buffers and to *q* < 0.3 Å^−1^ for SANS in H_2_O buffers. Also, inspection of the error-weighted residual plots (Supplementary Figs. S11*b* and S11*c* ) shows some differences, notably for urate oxidase and xylose isomerase that, as for the SAXS data, arise from small differences in the amplitudes and positions of the maxima and minima in the scattering profiles. The significantly greater error amplitudes for the SANS data result in smaller excursions in the residual plots. As was the case for the SAXS data, *WAXSiS*-predicted profiles required scaling and an additive constant.

All of the data and models described here, including *DAMMIN* calculations, have been deposited in SASBDB, and raw neutron data have been made available per Section 8.

## 5. Measurements beyond *q* = 1 Å^−1^

**Figure 9:**
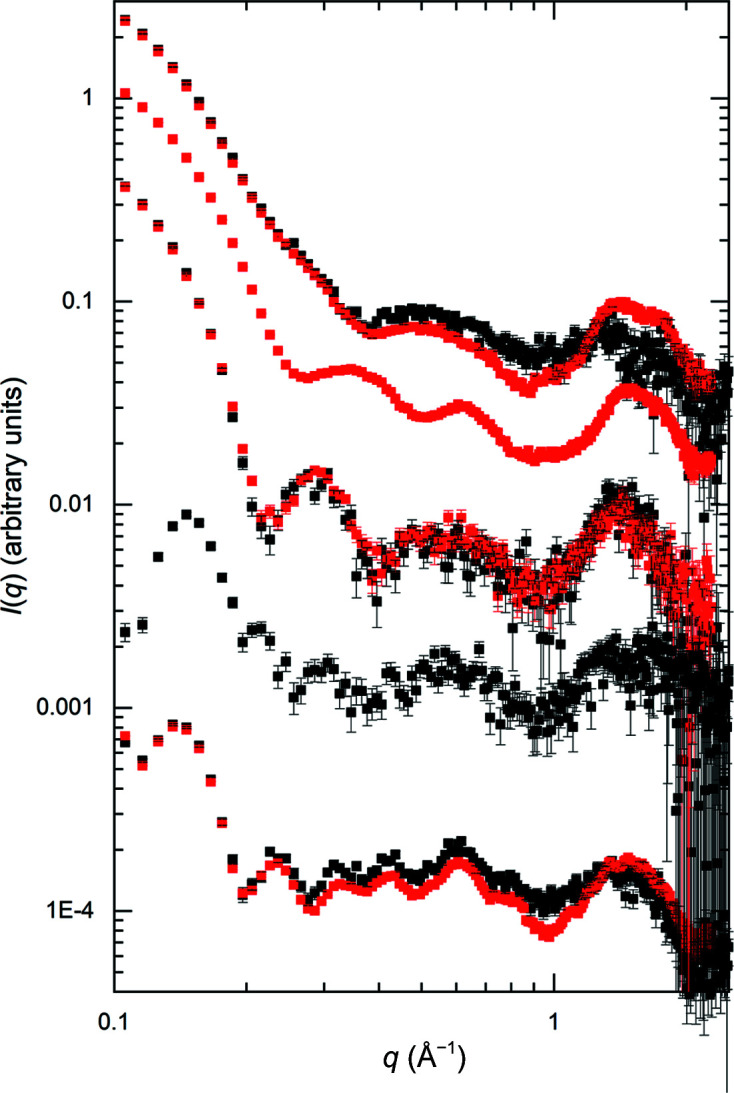
Data for (top to bottom traces) RNaseA, lysozyme, xylanase, urate oxidase and xylose isomerase from SEC–WAXS (black symbols, measured on the P12 BioSAXS beamline at EMBL, no lysozyme data) and batch WAXS (red symbols, measured on the 12-ID-B beamline at the APS, no urate oxidase data). The plot is log–log, with every second data point skipped for the sake of clarity and starting at *q* = 0.1 Å^−1^ to better highlight the data beyond *q* = 1 Å^−1^. Full log–log and log linear plots are given in Supplementary Fig. S12. Error bars are standard errors based on propagated counting statistics.

Two facilities measured SAXS data beyond *q* = 1 Å^−1^ (Fig. 9 ▸ and Supplementary Fig. S12). The P12 BioSAXS beamline at EMBL measured data to *q* = 2.65 Å^−1^ in SEC–WAXS mode, while data were acquired to *q* = 2.25 Å^−1^ in batch mode on the 12-ID-B beamline at APS. The batch-mode measurement allowed improved statistics in the high-*q* data, while the SEC–WAXS configuration gave uniform Δ*q* over the entire *q*-range using a sample subjected to SEC immediately before measurement. RNaseA, xylanase and xylose isomerase were each measured on both instruments, and each shows clear features beyond *q* = 1 Å^−1^ that are reproduced. Lysozyme was only measured on beamline 12-ID-B at APS, while urate oxidase was only measured on beamline P12 at EMBL. All five proteins show a broad feature in the scattering centred around *q* ≃ 1.5 Å^−1^. For RNaseA, the higher statistical quality of the data from APS beamline 12-ID-B allows the resolution of this broad feature into two peaks. This region includes scattering from the protein secondary structure, primary solvation layers and hydrophobic packing. The reproducibility of these WAXS profiles indicates promise for future studies aimed at detailed interpretation and modelling of these features, and for this purpose these WAXS data sets are available in the respective SASBDB entries for each protein (see Section 8).

## 6. Discussion

The SAXS and SANS data presented here were measured with sources that varied in brightness by orders of magnitude, from a rotating-anode X-ray source to synchrotrons of different generations and neutron instruments with distinct resolutions at three reactors that produce different neutron fluxes. Some instruments were not equipped with SEC–SAS. Nevertheless, the SAS profiles for each protein have proven to be reproducible, with the caveat that an additive constant adjustment was generally required to account for the difficulties in ensuring perfect solvent subtraction. The demonstrated reproducibility included the SAS-derived structural parameters and the overall SAS profile shape, including characteristic oscillations, for all five proteins. Further, most participating facilities contributed data for each protein. In the case of the two sets of independent SAXS measurements to *q* > 2.2 Å^−1^, there was also excellent reproducibility for the three proteins measured at both facilities.

This result is particularly significant when considering the logistical difficulties encountered due to the inherent fragility of biological samples, requirements for international shipping and limitations on access to beam time at largely oversubscribed instruments. Accurate solvent subtraction was especially challenging for the SANS data given the relatively poorer counting statistics achievable and the large incoherent scattering cross-section for ^1^H that is limiting for measurements in H_2_O. Even for measurements in D_2_O buffers, sufficient control of ^1^H content is challenging. These effects lead to significant uncertainties for solvent subtraction, and while under- or over-subtraction is typically tested for by releasing the *P*(*r*) = 0 at *r* = 0 constraint and adjusting the subtraction for improved *P*(*r*) properties, this procedure does not necessarily fully resolve subtraction issues, especially where there might be structural flexibility. Even so, the results obtained here showed the expected reduction in *R*_g_ and *d*_max_ values trending from measurements with SAXS to SANS in H_2_O to SANS in D_2_O.

Xylose isomerase proved to be the most robust of the proteins for SAS measurements, with no evident issues with aggregation in any of the SAS measurements. This protein is easily stabilized as an ammonium salt microcrystalline precipitate and this study has demonstrated that it can be stored for years without degrading. Further, it is soluble to very high concentrations and is unaffected by D_2_O in the solvent. However, critical to successful measurement of xylose isomerase was the avoidance of the interparticle interference that was consistently observed in the lowest *q* SAS regime if the concentration of this highly charged protein exceeded 1 mg ml^−1^, despite the charge-screening effects of the 100 m*M* NaCl present in the solvent. To overcome this problem, it was necessary to only use data acquired at <1 mg ml^−1^ in the low-*q* regime and to carefully merge with higher concentration data to achieve the optimal statistical quality of the measured *q*-range. Hampton Research no longer supplies the xylose isomerase used for this study and, given its favourable properties as a standard, there is an argument for pursuing options for supplying this protein to SAS users, perhaps via one of the national or international facilities that already support sample preparation or the supply of standards.

For urate oxidase, the necessity to ship solutions on ice and the upper limit on protein concentration of 5 mg ml^−1^ resulted in generally poorer statistics and a larger spread in Guinier *R*_g_ values compared with xylose isomerase that is attributable to a small degree of sample heterogeneity. Also, a few SAXS profiles for RNaseA were discarded due to evident severe aggregation, which was possibly radiation-induced. Xylanase proved to be a challenge for most facilities due to its unanticipated tendency to form persistent dimers.

Lysozyme samples showed the greatest spread in measured *R*_g_ values among the data combined to obtain a consensus SAXS measurement, and showed some unexpected behaviour with SANS in that the SEC–SANS H_2_O data appeared to have significant aggregation but not the batch H_2_O data or the SEC–SANS D_2_O data. Lysozyme has been a popular standard protein for SAXS measurements, and it is an important model protein more broadly. Conventionally, SAXS measurements have been made on solutions at low pH where there is a significant charge on the protein surface but monodispersity in solution is well established (Krigbaum & Kuegler, 1970 ▸). The current set of measurements were performed at pH 4.5 in 50 m*M* sodium citrate with 150 m*M* NaCl in order to provide charge screening to minimize charge repulsion and consequent interparticle interference effects. The citrate was also expected to act as a free-radical scavenger to provide protection from radiation-induced aggregation, to which lysozyme is known to be very sensitive. Nevertheless, lysozyme measurements appear to have had the twin issues of being vulnerable to potential interparticle interference and/or radiation-induced aggregation. The degree of variation in the SAXS *R*_g_ values from this study is similar to the distribution found among the eight lysozyme depositions currently in SASBDB that were measured at different pH values and concentrations (SASDBD entries SASDA96, SASDAC2, SASDAG2, SASDCK8, SASDMC2, SASDMD2, SASDME2 and SASDMF2 give a range from 14.2 to 15.2, providing that the latter four depositions that constitute a concentration series are extrapolated to zero concentration). For now, the consensus result for lysozyme obtained in this study is probably the most accurate currently available lysozyme SAS data set, but one would clearly like to improve on the reproducibility for this protein for it to be a useful SAS standard.

Inline SEC substantially reduced the probability of the presence of aggregates in the measured sample, and in the case of xylanase was essential for successful characterization of the monomeric form. Distributions of *R*_g_ [both Guinier- and *P*(*r*)-derived] and *d*_max_ values generally showed narrower distributions in SEC–SAXS compared with batch SAXS measurements. For the xylanase example, only SEC–SAXS and SEC–SANS measurements were characteristic of the monomeric form. For RNaseA and lysozyme in D_2_O, merging SEC–SANS with batch data was essential to remove the influence of aggregates. Thus, if inline SEC is available it is the preferred approach to obtaining a monodisperse sample for SAS measurements, which is the fundamental requirement for interpretation in terms of a single structure. That said, the potential for the dissociation of complexes during SEC and the increased exposure to radiation damage for more dilute solutions must be considered in planning experiments. Further, the xylanase example is a cautionary tale given that in four of the eight SEC–SAXS measurements small amounts of dimer were not removed. It is therefore highly desirable to use SAS-independent measures to evaluate samples for their tendency towards oligomerization or aggregation before SAS measurements, noting here that the MALLS data for xylanase did indicate that dimer formation was a potential issue, and that better separation was achieved compared with inline SEC by using a larger column and a different tubing setup that decreased the band broadening. Of course, inline SEC comes at the cost of lower sample concentrations due to the unavoidable dilution during elution from the column and hence lower signal to noise, especially in the medium-to-high *q*-range. This effect is nicely demonstrated by comparison of the WAXS data collected to *q* > 2.2 Å^−1^ with and without inline SEC. The successful merging of SEC–SAS data with batch data overcame this limitation.

The issues noted above bring to the fore the fact that even for well characterized proteins it takes significant attention to the details of sample preparation, data acquisition, reduction and analysis to obtain a reliable SAS result. The issues that were encountered in this study will only be amplified for less well known systems. Even for these very well known and relatively stable proteins, it was essential to use all of the measures specified in the 2017 publication guidelines for biomolecular SAS (Trewhella *et al.*, 2017 ▸), including utilizing non-SAS methods for initial sample characterization, to have confidence in the final analysis.

The *datcombine* tool was developed to optimally combine data from different instruments, with optional filters to remove outlier data points or data that only served to increase the errors. While alternate methods are available for combining data, for example *Merge* in *primusQt* or *SAXS Merge* within the Integrative Modelling Platform (Spill *et al.*, 2014 ▸), we are unaware of any methods that, in addition to scaling, include scaling plus constant adjustment for optimal agreement with minimization of the global discrepancy (in terms of all pairwise profile comparisons) plus outlier and large error data-point filtering. It should be noted that *datcombine* can be more broadly used, for example for averaging measurements with different concentrations of sample taken at a single instrument. In its application here, the low dispersion of scattering profiles, albeit subjected to minimization of differences by the application of an adjustable scale factor and constant addition, demonstrated the high degree of reproducibility in the independent measurements and legitimized their subsequent combination to yield a consensus curve. The application of outlier and/or error filters provides a consensus curve of the highest statistical quality possible. For the SAXS measurements, consensus SAXS profiles to *q* = 1 Å^−1^, or in the case of xylanase to *q* = 0.7 Å^−1^, provide excellent target scattering profiles for models with atomic detail. While the SANS data are inherently limited regarding statistical quality in the mid- and high-*q* regimes, the region of the scattering profile that determines size and overall shape is well determined and can be useful in examining models for hydration-layer effects. We note here that we did not attempt to account for the *q*-scale smearing of the measured SANS data that results from the larger beam dimensions and wavelength spread that are required to compensate for the much lower neutron fluxes compared with X-rays. Such an undertaking would be extremely complex and is beyond the scope of this study. Furthermore, the reproducibility of the individual measured SANS profiles with their nominal *q* scales and the general good agreement between *R*_g_ values and predicted SANS profiles for the consensus curves demonstrate that the smearing effects are not so large so as to undermine the basic conclusions of the study.

In using the consensus profiles, it is important to keep in mind that in merging or combining data from different sources and using filtering options one can obtain a distribution of errors in *I*(*q*) versus *q* that is distinct from the generally steady change in uncertainty with *q* obtained for the typical SAS profile of a protein from measurement on a single instrument in a fixed configuration. When using an error-weighted least-squares fit of SAS data, such as that implemented in *GNOM*, to calculate *P*(*r*), the result can be sensitive to the distribution of experimental errors. Indeed, while the main peak of the *P*(*r*) profiles obtained from *GNOM* using the *datcombine*-generated scattering profiles with and without filters applied was quite stable, we sometimes observed differences in how the profile terminated around *r* = *d*_max_, and for the consensus SAXS profiles where the propagated errors were smallest there could be small oscillations near *d*_max_.

A variety of atomistic modelling methods showed qualitative good agreement with the overall shapes of experimental scattering profiles from crystal structure coordinates, noting the possibility of improved fits for RNaseA using the NMR solution conformers and the fact that the residual difference plots indicate the potential for improvement. There would be great benefit to SAXS and SANS researchers if different modelling calculations could be accessed from a single point and accept a single set of inputs. Such a resource is under development (co-authors EB and CJ) as a web-based tool to conveniently compare the results of different SAXS profile calculators, built using the *GenApp* framework (Savelyev & Brookes, 2019 ▸) that was originally developed under the *Collaborative Computational Project for SAS* (Perkins *et al.*, 2016 ▸). Community support for such a resource would be of broad benefit, especially for attracting new SAS users.

## 7. Conclusions

With the growing uptake of guidelines for the publication of modelling results from SAS data along with tools for model validation, it was timely to use a round-robin approach to obtain high-quality SAS data over an extended *q*-range from a set of proteins that would be good candidates to use in benchmarking different approaches to the prediction of SAS profiles from atomic coordinates and in the process test the limits of experimental reproducibility. The experimental data on the five proteins studied here demonstrate a high level of reproducibility for this set of relatively well characterized proteins, as well as the limits, for example, in the accuracy of solvent subtraction. The value of adherence to the 2017 guidelines for publication of biomolecular SAS and 3D modelling is well demonstrated. The five consensus SAS profiles obtained provide a core set of consensus SAS data for evaluating, comparing and potentially improving any one approach to theoretical SAS profile prediction.

It is desirable to extend the methods employed here to improve the consensus scattering profiles, especially in the cases of lysozyme and xylanase. All of the original data used to generate the consensus profiles, including the raw SANS data with resolution information calculated based on the geometry and optics of the instrument configuration, are publicly available, so that if new methods for combining data are developed an improved set of consensus data sets may be produced. Furthermore, there will be continuous improvement in beamlines, instruments, data reduction, analysis and sample preparation. Newly collected data from new instrumentation with new procedures on high-quality samples would be expected to increase the reproducibility and ultimately the quality achievable for a consensus profile. The approach used here can be extended to any suitable protein studied using SAS by any group in the world thus motivated. An insightful reviewer of this manuscript pointed out that because active sites commonly require structural flexibility, their exposure to solvent can render a protein more vulnerable to aggregation. In this study, the most robust protein for SAS measurement, with no samples showing signs of aggregation, was xylose isomerase, which has internally oriented active sites. In contrast, urate oxidase has externally oriented active sites, while each of the three monomeric proteins have surface-exposed active sites. Consideration of this property could be added to those outlined in Section 2 for selecting future candidate proteins. Provided that the protein can be made available for measurement on a reasonable number of instruments, data could be collected in a similar way to the measurements reported here and, after comparative analysis, the consensus scattering profile could be added to this core set, thus steadily improving upon and enlarging the benchmark SAS pattern set for prediction. Just one new protein a year would result in a doubling of the core set provided here in just five years.

## 8. Data-deposition details

The consensus data and model fits with zip folders containing the individual contributing scattering profiles have been deposited in SASBDB (Kikhney *et al.*, 2020 ▸; SASDPP4, SASDPQ4, SASDPR4, SASDPS4 and SASDPT4 are for consensus SAXS profiles for RNaseA, urate oxidase, xylose isomerase, xylanase and lysozyme, respectively; SASDPU4, SASDPV4, SASDPW4, SASDPX4 and SASDPY4 are for consensus SANS profiles measured in D_2_O buffer for RNaseA, lysozyme, xylanase, urate oxidase and xylose isomerase, respectively; SASDPZ4, SASDP25, SASDP35, SASDP45 and SASDP55 are for consensus SANS profiles measured in H_2_O buffer for lysozyme, RNaseA, xylanase, urate oxidase and xylose isomerase, respectively). Additional WAXS data (SEC–WAXS and batch) are made available in the full entry zip archives of the respective SASBDB entries for each protein. For the SANS data submissions, the associated zip files include the unsubtracted six-column format batch data with resolution information for all sample and solvent measurements, plus solvent-subtracted six-column format SEC–SANS ILL/D22 data. In addition, raw SANS and SEC–SANS data recorded on the ILL D22 instrument are available at https://doi.ill.fr/10.5291/ILL-DATA.INTER-465. Raw SANS data from ANSTO/Quokka are available from the Zenodo digital archive at https://doi.org/10.5281/zenodo.6789723. The simulation systems used for *WAXSiS* calculations also are available via the Zenodo digital archive at https://doi.org/10.5281/zenodo.7057567.

## 9. Related literature

The following references are cited in the supporting information for this article: Basham *et al.* (2015 ▸), Berendsen *et al.* (1984 ▸), Blanchet *et al.* (2015 ▸), Brookes & Rocco (2022 ▸), Bussi *et al.* (2007 ▸), Cantor & Schimmel (1980 ▸), Chatzimagas & Hub (2022 ▸), Chen *et al.* (2019 ▸), Classen *et al.* (2013 ▸), Cohn & Edsall (1943 ▸), Cowieson *et al.* (2020 ▸), Cromer & Mann (1968 ▸), Durchschlag & Zipper (1994 ▸), Dyer *et al.* (2014 ▸), Essmann *et al.* (1995 ▸), Franke *et al.* (2012 ▸, 2015 ▸), Gerstein & Chothia (1996 ▸), Hajizadeh *et al.* (2018 ▸), Harding *et al.* (1992 ▸), Hess (2008 ▸), Hopkins *et al.* (2017 ▸), Hornak *et al.* (2006 ▸), Jorgensen *et al.* (1983 ▸), Joung & Cheatham (2008 ▸), Kirby *et al.* (2013 ▸, 2016 ▸), Kline (2006 ▸), Kuntz & Kauzmann (1974 ▸), Lindorff-Larsen *et al.* (2010 ▸), Li *et al.* (2016 ▸), Liu *et al.* (2018 ▸), Miyamoto & Kollman (1992 ▸), Panjkovich & Svergun (2018 ▸), Rocco *et al.* (2020 ▸), Sousa da Silva & Vranken (2012 ▸), Thureau *et al.* (2021 ▸), Walker *et al.* (2008 ▸), Wei *et al.* (2004 ▸), Wood *et al.* (2018 ▸) and Wu *et al.* (2020 ▸).

## Supplementary Material

SASBDB reference: SAXS data: ribonuclease A, SASDPP4

SASBDB reference: urate oxidase, SASDPQ4

SASBDB reference: xylose isomerase, SASDPR4

SASBDB reference: xylanase, SASDPS4

SASBDB reference: lysozyme, SASDPT4

SASBDB reference: SANS data: ribonuclease A in D_2_O buffer, SASDPU4

SASBDB reference: lysozyme in D_2_O buffer, SASDPV4

SASBDB reference: xylanase in D_2_O buffer, SASDPW4

SASBDB reference: urate oxidase in D_2_O buffer, SASDPX4

SASBDB reference: xylose isomerase in D_2_O buffer, SASDPY4

SASBDB reference: lysozyme in H_2_O buffer, SASDPZ4

SASBDB reference: ribonuclease in H_2_O buffer, SASDP25

SASBDB reference: xylanase in H_2_O buffer, SASDP35

SASBDB reference: urate oxidase in H_2_O buffer, SASDP45

SASBDB reference: xylose isomerase in H_2_O buffer, SASDP55

Supplementary text, Figures and Tables. DOI: 10.1107/S2059798322009184/cb5140sup1.pdf

## Figures and Tables

**Table 1:** Sample details

	RNaseA	Lysozyme	Xylanase	Urate oxidase	Xylose isomerase
Organism	*Bos taurus* (pancreas)	*Gallus gallus* (hen egg white)	*Trichoderma reesei*	*Aspergillus flavus*	*Streptomyces rubiginosus*
Source (catalogue No. or reference)	Sigma–Aldrich R6513	Sigma–Aldrich L6876 or L4919	Hampton Research HR7-104	Sanofi–Aventis, *Pichia pastoris* expression	Hampton Research HR7-102
Description: UniProt ID (sequence range in construct)	P61823 (27–150)	P00698 (19–147)	F8W669 (1–190)	Q00511 (2–302) with acetylated N-terminal Ser and 8-azaxanthine inhibitor: C_4_H_3_N_5_O_2_	P24300 (1–388)
Calculated extinction coefficient ɛ, *A*_280_ 0.1%					
From sequence†	0.69	2.65	2.80	1.56	1.07
8-Azaxanthine‡				0.28	
Sequence + xanthine				1.84	
Calculated partial specific volume 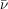 § (cm^3^ g^−1^, 20°C)	0.710	0.716	0.712	0.735	0.727
Mean protein and solvent scattering length densities¶ (10^10^ cm^−2^)	12.621, 9.469	12.507, 9.484	12.518, 9.469	12.360, 9.489	12.363, 9.470
Mean scattering contrast¶ (10^10^ cm^−2^)	3.151	3.023	3.049	2.871	2.893
Molecular mass from chemical composition† (Da)	13690.3 (monomer)	14313.1 (monomer)	20843.6 (monomer)	34150.7 (protomer), 136603 (tetramer)	43227.4 (protomer), 172910 (tetramer)
Molecular mass from mass spectrometry†† (Da)	—	—	20825.0	34151.4 (protomer)	43227.6 (protomer)
Standard solvent composition	50 m*M* Tris pH 7.5, 100 m*M* NaCl	50 m*M* sodium citrate pH 4.5, 150 m*M* NaCl	50 m*M* Tris pH 7.5, 100 m*M* NaCl	100 m*M* Tris pH 8.0, 150 m*M* NaCl	50 m*M* Tris pH 7.5, 100 m*M* NaCl, 1 m*M* MgCl_2_

† Calculated using *ProtParam* (Gasteiger *et al.*, 2005 ▸).

‡ Experimentally determined.

§ Calculated using *SEDNTERP* (Philo, 1997 ▸); see also Section S2 and Supplementary Table S2.

¶ Calculated using *MULCh* (Whitten *et al.*, 2008 ▸).

†† Performed at Sydney Mass Spectrometry. Note: urate oxidase shows a second resolved peak at 34 169 Da.

**Table 2:** Mean structural parameters from SAXS data Batch SAXS and SEC–SAXS data were analysed individually, and the table reports the average value of each parameter with one standard deviation of their distribution given in parentheses. *V*_P_/*m* is the ratio of the Porod volume (*V*_P_) to the molecular mass (*m*). *R*_g_ values for the consensus profiles are quoted with standard errors.

Protein	Batch SAXS	SEC–SAXS	Consensus profile
RNaseA			
*R*_g_, Guinier (Å)	15.66 (0.26)	15.08 (0.08)	15.13 ± 0.02
*R*_g_, *P*(*r*) (Å)	15.55 (0.31)	15.02 (0.08)	15.04 ± 0.01
*d*_max_ (Å)	50 (2)	48 (1)	49
*V*_P_ (Å^3^)	16222 (682)	15784 (577)	17626
*V*_P_/*m*	1.18	1.15	1.29
Lysozyme			
*R*_g_, Guinier (Å)	15.32 (0.81)	15.05 (0.45)	14.64 ± 0.05
*R*_g_, *P*(*r*) (Å)	15.33 (0.87)	14.98 (0.38)	14.46 ± 0.01
*d*_max_ (Å)	49 (5)	48 (3)	48
*V*_P_ (Å^3^)	19859 (4013)	20324 (2553)	18725
*V*_P_/*m*	1.38	1.42	1.31
Xylanase			
*R*_g_, Guinier (Å)	17.18 (0.45)	16.22 (0.22)	16.05 ± 0.01
*R*_g_, *P*(*r*) (Å)	17.42 (0.60)	16.17 (0.43)	15.85 ± 0.01
*d*_max_ (Å)	66 (7)	58 (10)	51
*V*_P_ (Å^3^)	28601 (5143)	26415 (3698)	27151
*V*_P_/*m*	1.37	1.27	1.30
Urate oxidase			
*R*_g_, Guinier (Å)	32.72 (0.53)	31.96 (0.66)	32.30 ± 0.06
*R*_g_, *P*(*r*) (Å)	32.18 (0.81)	31.48 (0.51)	31.63 ± 0.01
*d*_max_ (Å)	104 (21)	88 (4)	92
*V*_P_ (Å^3^)	217966 (30633)	217723 (3777)	219837
*V*_P_/*m*	1.60	1.59	1.61
Xylose isomerase			
*R*_g_, Guinier (Å)	33.12 (0.31)	33.15 (0.22)	33.11 ± 0.05
*R*_g_, *P*(*r*) (Å)	32.96 (0.34)	32.83 (0.08)	32.93 ± 0.01
*d*_max_ (Å)	98 (3)	97 (2)	101
*V*_P_ (Å^3^)	234078 (5839)	239819 (7908)	243121
*V*_P_/*m*	1.35	1.39	1.41

**Table 3:** Structural parameters determined from SANS data Values for batch data are the average of individual analyses of multiple batch measurements, with one standard deviation given in parentheses. SEC–SANS values are from a single measurement with standard errors, while the consensus profile is from the optimal combination of batch and SEC–SANS data as described in the text, also with standard errors.

	Batch SANS H_2_O	SEC–SANS H_2_O	Consensus profile	Batch SANS D_2_O	SEC–SANS D_2_O	Consensus profile
RNaseA						
*R*_g_, Guinier (Å)	14.93 (0.39)	14.38 ± 0.38	14.74 ± 0.17	14.20 (0.52)	13.64 ± 0.07	13.67 ± 0.08
*R*_g_, *P*(*r*) (Å)	15.00 (0.42)	14.60 ± 0.16	14.50 ± 0.13	14.19 (0.46)	13.78 ± 0.07	13.72 ± 0.05
*d*_max_ (Å)	47 (3)	43	41	46 (2)	46	44
Lysozyme						
*R*_g_, Guinier (Å)	15.11 (0.71)	15.77 ± 0.41	14.28 ± 0.12	13.6 (0.33)	12.16 ± 0.42	12.44 ± 0.52
*R*_g_, *P*(*r*) (Å)	14.99 (0.68)	15.57 ± 0.27	14.66 ± 0.12	13.56 (0.28)	12.81 ± 0.48	12.23 ± 0.10
*d*_max_ (Å)	47 (1)	48	48	46 (2)	45	38
Xylanase						
*R*_g_, Guinier (Å)	16.82 (0.39)	15.87 ± 0.38	16.03 ± 0.30	15.86 (0.77)	14.65 ± 0.24	14.87 ± 0.19
*R*_g_, *P*(*r*) (Å)	16.89 (0.37)	15.38 ± 0.17	15.02 ± 0.19	15.69 (1.03)	14.58 ± 0.07	14.43 ± 0.05
*d*_max_ (Å)	55 (6)	43	43	53 (5)	46	44
Urate oxidase						
*R*_g_, Guinier (Å)	31.66 (1.01)	30.34 ± 2.43	31.57 ± 0.42	32.32 (1.90)	31.50 ± 0.34	31.18 ± 0.12
*R*_g_, *P*(*r*) (Å)	32.25 (1.37)	29.52 ± 0.39	31.67 ± 0.23	30.92 (0.44)	30.03 ± 0.06	30.84 ± 0.04
*d*_max_ (Å)	94 (4)	83	91	91 (2)	83	93
Xylose isomerase						
*R*_g_, Guinier (Å)	32.00 (1.19)	31.51 ± 1.58	32.61 ± 0.38	30.79 (0.69)	30.59 ± 0.12	30.72 ± 0.17
*R*_g_, *P*(*r*) (Å)	32.21 (0.65)	33.01 ± 0.49	32.30 ± 0.18	30.97 (0.68)	30.58 ± 0.05	30.88 ± 0.08
*d*_max_ (Å)	93 (6)	95	97	93 (6)	90	95
