# Supplementary material for: A round-robin approach provides a detailed assessment of biomolecular small-angle scattering data reproducibility and yields consensus curves for benchmarking
Source: Acta Crystallogr D Struct Biol. 2022 Oct 20;78(Pt 11):1315–36. doi: 10.1107/S2059798322009184 (PMC9629491; doi:10.1107/S2059798322009184)
Supplement: Supplementary file 1 [file d-78-01315-sup1.pdf]

Acta Cryst D
STRUCTURAL BIOLOGY

Volume 78 (2022)

Supporting information for article:

A round-robin approach provides a detailed assessment of biomolecular small-angle scattering data reproducibility and yields consensus curves for benchmarking

Jill Trewhella, Patrice Vachette, Jan Bierma, Clement Blanchet, Emre Brookes, Srinivas Chakravarthy, Leonie Chatzimagas, Thomas E. Cleveland, Nathan Cowieson, Ben Crossett, Anthony P. Duff, Daniel Franke, Frank Gabel, Richard E. Gillilan, Melissa Graewert, Alexander Grishaev, J. Mitchell Guss, Michal Hammel, Jesse Hopkins, Qingqui Huang, Jochen S. Hub, Greg L. Hura, Thomas C. Irving, Cy Michael Jeffries, Cheol Jeong, Nigel Kirby, Susan Krueger, Anne Martel, Tsutomu Matsui, Na Li, Javier Pérez, Lionel Porcar, Thierry Prangé, Ivan Rajkovic, Mattia Rocco, Daniel J. Rosenberg, Timothy M. Ryan, Soenke Seifert, Hiroshi Sekiguchi, Dmitri Svergun, Susana Teixeira, Aurelien Thureau, Thomas M. Weiss, Andrew E. Whitten, Kathleen Wood and Xiaobing Zuo

S1. Explicit-solvent SAXS/SANS calculations with the WAXSiS method

Custom WAXSiS-type calculations were performed locally in the Hub laboratory (Chatzimagas and Hub, Saarland University). The SAXS and SANS calculations were based on explicit-solvent all-atom molecular dynamics (MD) simulations. The starting configurations for the all-atom MD simulations are taken from published crystal structure coordinates of RNaseA (7RSA), lysozyme (2VB1), xylanase (2DFC), urate oxidase (3l8W), and xylose isomerase (1MNZ), modified as noted in the main text (**3.4**). Crystallization agents and other buffer molecules were removed for all structures.

Simulations were carried out with the Gromacs software (Abraham *et al.*, 2015) version 2021.3. The proteins were placed in dodecahedral simulation box, where the distance between the protein to the periodic boundaries was at least 2 nm. The boxes were subsequently filled with TIP3P water (Jorgensen *et al.*, 1983), and sodium and chloride ions were added to match the experimental NaCl concentrations of 150 mM, 100 mM, 100 mM, 100 mM, 150 mM for RNaseA, lysozyme, xylanase, xylose isomerase, and urate oxidase, respectively, as well as magnesium and chloride ions to match experimental $MgCl_2$ concentration of 1 mM for xylose isomerase. Additional sodium and chloride ions were added to neutralize the system. In total, the systems contained between 46,848 and 210,699 atoms. Interactions of the protein and ions were described with the AMBER99SB-ILDN (Lindorff-Larsen *et al.*, 2010, Hornak *et al.*, 2006) force field and using ion parameters described in (Joung & Cheatham, 2008). The inhibitor xanthin was parametrized with ACPYPE (Sousa da Silva & Vranken, 2012) based on ANTECHAMBER (Wei *et al.*, 2004) using parameters from the AMBER99SB (Hornak *et al.*, 2006) and the atomic partial charges determined by SQM (Walker *et al.*, 2008) using AM1-BCC.

The energy of each simulation system was minimized within 2000 steps using the steepest descent algorithm. Subsequently, the simulation systems were equilibrated for 100 ps with harmonic position restraints applied to the heavy atoms (force constant 1000 kJ mol^{-1} nm^{-2}). Production simulations were run for 50 ns with harmonic position restrains (force constant 1000 kJ mol^{-1} nm^{-2}) on the backbone atoms. Frames were written every 10 ps. The temperature was kept at 298.15 K using velocity rescaling ($\tau = 0.1$ ps) (Bussi *et al.*, 2007) The pressure was controlled at 1 bar with the Berendsen barostat ($\tau = 2$ ps) (Berendsen, 1984). The geometry of water molecules was constrained with the SETTLE algorithm (Miyamoto & Kollman, 1992), and LINCS (Hess, 2008) was used to constrain other bond lengths involving hydrogen atoms. An integration time step of 2 fs was used. The Lennard-Jones potentials with a cut-off at 1.2 nm were used to describe dispersive interactions and short-range repulsion. Electrostatic interactions were computed with the smooth particle-mesh Ewald method (Essmann *et al.*, 1995).

Explicit-solvent SAXS and SANS calculations (Chatzimagas & Hub, 2022, Knight & Hub, 2015) were performed with the rerun functionality of an in-house modification of Gromacs 2018.8, as also implemented on the webserver WAXSiS (Knight & Hub, 2015). The source code and documentation are available on GitLab at https://gitlab.com/cbjh/gromacs-swaxs and https://cbjh.gitlab.io/gromacs-swaxs-docs, respectively. A spatial envelope was built around the protein keeping a distance of 0.7 nm from all solute atoms in all simulation frames. Solvent atoms inside the envelope contributed to the SAS calculations, thereby accounting for the modified density of the hydration layer. The buffer subtraction was carried out using 5000 simulation frames from pure-buffer simulation boxes whose salt content closely matched the respective solutes simulations and which were large enough to enclose the envelopes. The buffer simulations were carried out for 50 ns. The orientational average was carried out using 4000 q-vectors for each absolute value of q, and the solvent electron density was corrected to the experimental

value of 334 e/nm3, as described previously (Chen & Hub, 2014). For SAXS calculations, atomic form factors were modelled as four Gaussians described with the Cromer-Mann-Parameters (Cromer & Mann, 1968). For SANS calculations, the coherent neutron scattering lengths were applied. In SANS calculations (Chen *et al.*, 2019) the D_2O concentrations of 0% and 100% were taken into account according to the experimental conditions.

S2. Derivation of approximate V_p/m ratio

The derivation of the approximate Porod volume/molecular mass ratio (V_p/m) for a globular, folded protein depends on the values of the partial specific volume ($\bar{\vartheta}$) and the degree of hydration α (m_{H2O}/m) where m_{H2O} is the mass of the associated hydration layer in grams. Values for $\bar{\vartheta}$ and α can be calculated for a protein using established methods as implemented in public domain programs such as SEDNTERP3 from the chemical composition (http://www.jphilo.mailway.com/sednterp.htm) or US-SOMO from structures (https://somo.aucsolutions.com/index.php). There are slight differences between the values computed by the two programs for the same protein. Both rely on tabulated molar volumes in solution, SEDNTERP3 being based on the original work of Cohn and Edsall (Cohn & Edsall, 1943) as reported for T = 25 °C in (Harding *et al.*, 1992), while US-SOMO is based on the extended work of (Durchschlag & Zipper, 1994). The two programs calculate by default the $\bar{\vartheta}$ values at T = 25 and 20 °C, respectively, with the possibility of calculating at any given T. For the calculation of α based on the amino acid composition, both programs rely on the original NMR freezing work of (Kuntz & Kauzmann, 1974). SEDNTERP3 offers a calculation at pH 7 and one at pH < 4, while US-SOMO has recently implemented a full pH range-based calculation (Rocco *et al.*, 2020).

As a first approximation for V_p/m for a "typical" folded protein, average values of $\bar{\vartheta}$ were computed utilizing the recently released US-SOMO-AlphaFold (AF) database (Brookes & Rocco, 2022), which contains the computed solution properties of >1,000,0000 AlphaFold-predicted structures, including the full UniProt dataset (https://somo.genapp.rocks/somoaf). A statistical analysis of the $\bar{\vartheta}$ distribution provides an average value of 0.737 cm^3/g (without the contributions of any prosthetic groups as they are not present in the AlphaFold structures), with a full width at half height of ± 0.028 cm^3/g for the 99% confidence interval. Assuming this average $\bar{\vartheta}$ for a "dry" (anhydrous, "naked") protein and expressing it in $Å^3\,Da^{-1}$ (noting that 1 Da = 1.66 10^{-24}g or 1g= (1/1.66) 10^{24} Da) we obtain:

$$\bar{\vartheta} = \frac{0.737 \cdot 10^{24}}{\left(\frac{1}{1{,}66}\right) 10^{24}}\ Å^3 Da^{-1} \quad = 0.737 \cdot 1.66 = 1.225\ Å^3\ Da^{-1}$$

Giving an estimate for the volume of an anhydrous naked protein ($V_{anhydrous}$) of molecular mass m :
$V_{anhydrous} = 1.225 \cdot m$

However, V_p is the hydrated volume, and so

$$V_p = (1.225 + \alpha r_h)m$$

Where r_h is the ratio of the volume occupied by the average hydration water (24.5 $Å^3$) to that of bulk water (29.7 $Å^3$) (Gerstein & Chothia, 1996), i.e.

$r_h = \frac{24.5}{29.7} = 0.825$, then

Typical values of α are 0.3 – 0.4 g_{H2O}/g_{prot} (pages 550-552 (Cantor & Schimmel, 1980))

$$\frac{V_P}{m} = 1.47 - 1.55$$

This approximate range has been confirmed in a systematic calculation performed using the US-SOMO-AlphaFold database. The statistical analysis of the distribution yields an average value for α of 0.362 ± 0.037. For monomeric proteins without prosthetic groups, entering the Uniprot code gives immediate access to both $\bar{\vartheta}$ and α values from the US-SOMO-AlphaFold database. For other proteins, one can calculate their theoretical $\bar{\vartheta}$ and α values, using either SEDNTERP3 or US-SOMO, which we have done for the five reference proteins from this study and find they lie in the range 1.43 – 1.53 (**Table S1**). These estimates are a guide. Typical practice has been to consider ratios as large as 1.6 – 1.7 as an acceptable demonstration for mono-dispersity for a protein in solution. However, developments in instrumentation that give greater accuracy in solvent subtraction with in-line SEC for removing even small amounts of sample heterogeneity would be expected to reduce this upper range. There is also an inherent uncertainty in the experimentally determined V_p that depends upon an integral from 0 – infinity when data are only measured from q_{min} to q_{max}.

Figure S1 SDS-PAGE gels for xylanase and xylose isomerase.

Denaturing gel electrophoresis was performed 10 May 2019 prior to shipment of these samples as a check for purity. The major bands for both xylanase and xylose isomerase are observed as expected for the monomer forms. Weak higher molecular weight bands appear to be trace contaminants.

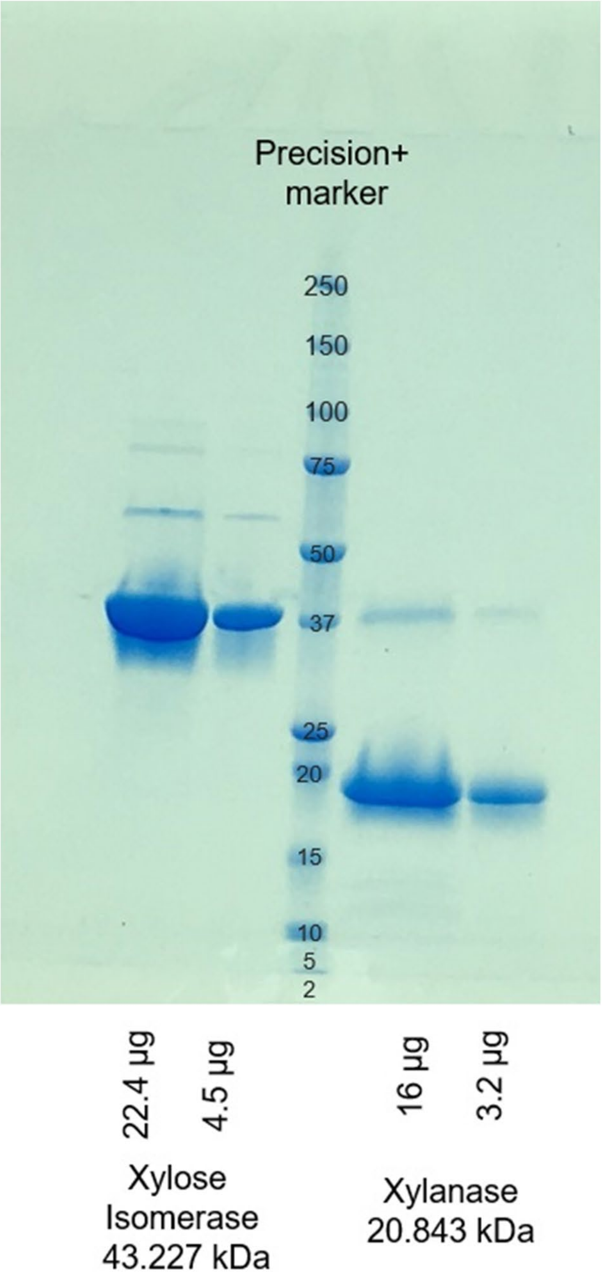

Figure S2 The deconvoluted electrospray ionisation – time-of-flight mass spectra for xylanase, urate oxidase and xylose isomerase.

In preparation for mass spectrometry analysis, xylose isomerase and urate oxidase were dialysed into 20 mM (M = mol/L) ammonium bicarbonate (pH 6.9 and pH 8.0, respectively) while xylanase was dialyzed into 50 mM ammonium formate (pH 4.0). The dialysed xylose isomerase and urate oxidase were mixed 50:50 with 20% acetonitrile, 0.2% formic acid, while the xylanase was mixed 90:10 with 100% acetonitrile. Samples then were directly infused at 50 µl/min into a quadrupole-time-of-flight tandem mass spectrometer (TripleTOF 6600, Sciex) via electrospray ionisation (Sydney Mass Spectrometry, University of Sydney). The mass spectra collected were deconvoluted using PeakView (version 2.2, Sciex). The mass values (Da) of the major peaks are displayed. The major observed masses for xylanase, urate oxidase and xylose isomerase are within 20 ppm of the expected mass, with additional peaks that are most likely sodium or potassium adducts.

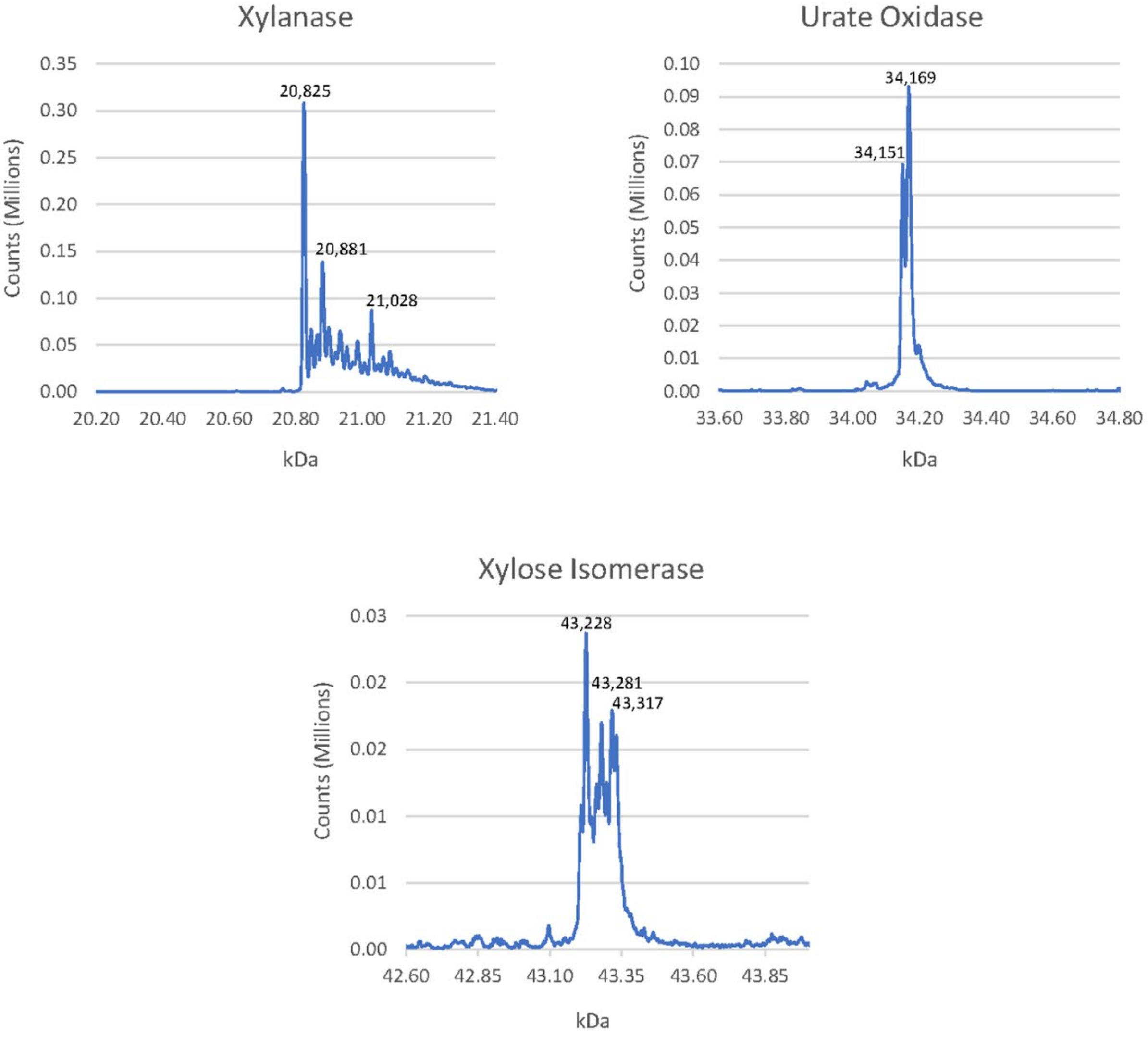

Figure S3 Histograms showing distribution of structural parameters for RNase A, lysozyme, xylanase, for batch (panels **A** and **C**) and SEC-SAXS (panels **B** and **D**) data.

Panels are arranged in vertically placed pairs to highlight systematic differences between results for different measurement modes, which are most evident for RNaseA and xylanase. The same key as in Guinier batch data panel is used for all panels.

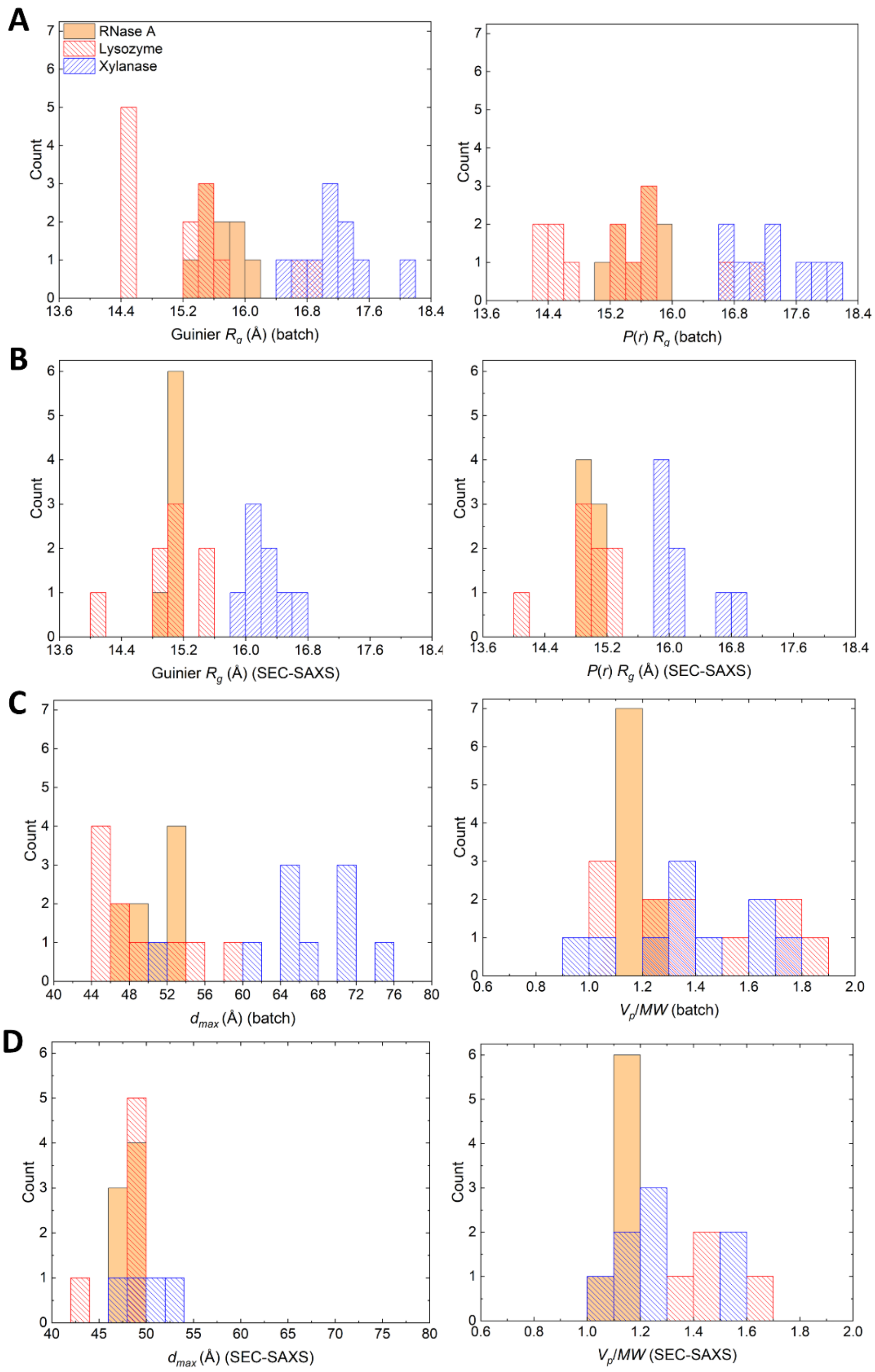

Figure S4 Histograms showing distribution of structural parameters for urate oxidase and xylose isomerase for batch (panels **A** and **C**) and SEC-SAXS (panels **B** and **D**) data.

Panels are arranged in vertically placed pairs to highlight any systematic differences between results for different measurement modes, which are more evident for urate oxidase. One urate oxidase sample was very aggregated with R_g > 33 Å and its d_{max} value (156 Å) is off scale. The same key as in Guinier batch data panel is used for all plots.

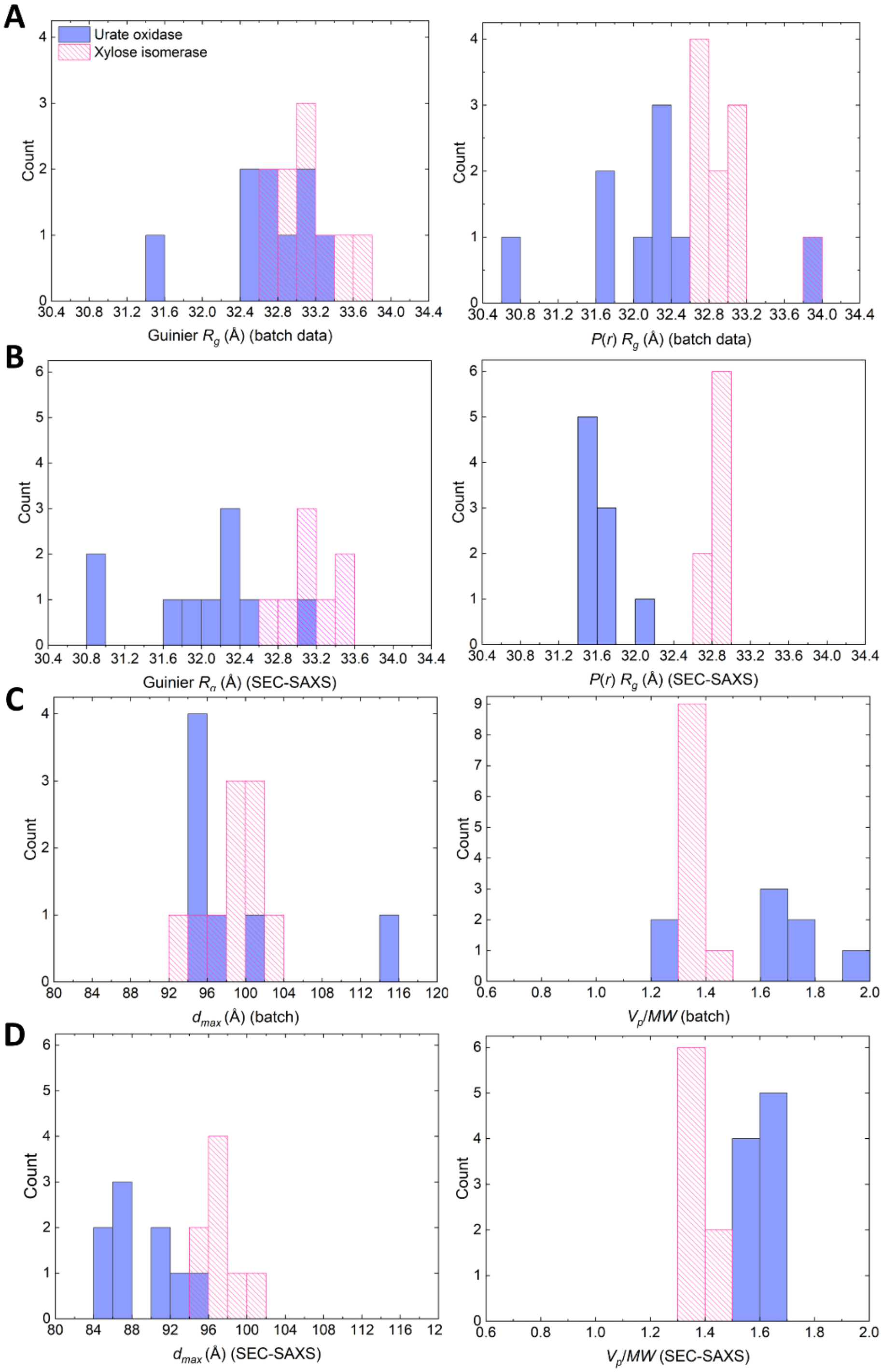

Figure S5 SAXS data used to generate the consensus profiles for **A.** RNase A **B.** xylose isomerase collected on different instruments that have been re-gridded to a common q-scale and scaled.

Variations in background levels are highlighted by the inserts with expanded vertical and horizontal scales. The data are represented by a different coloured symbol for each instrument, with every 2nd point dropped for clarity.

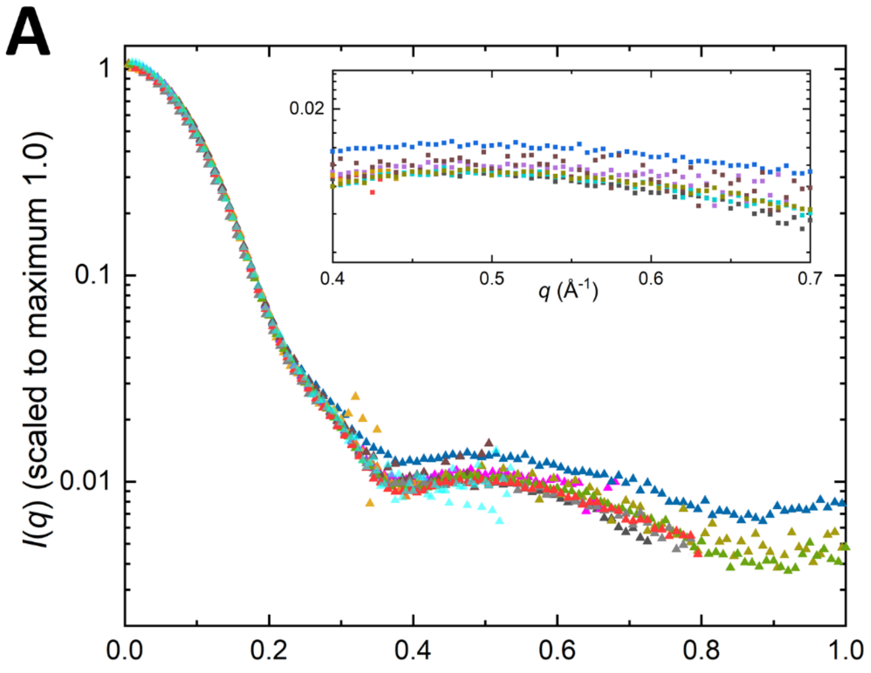

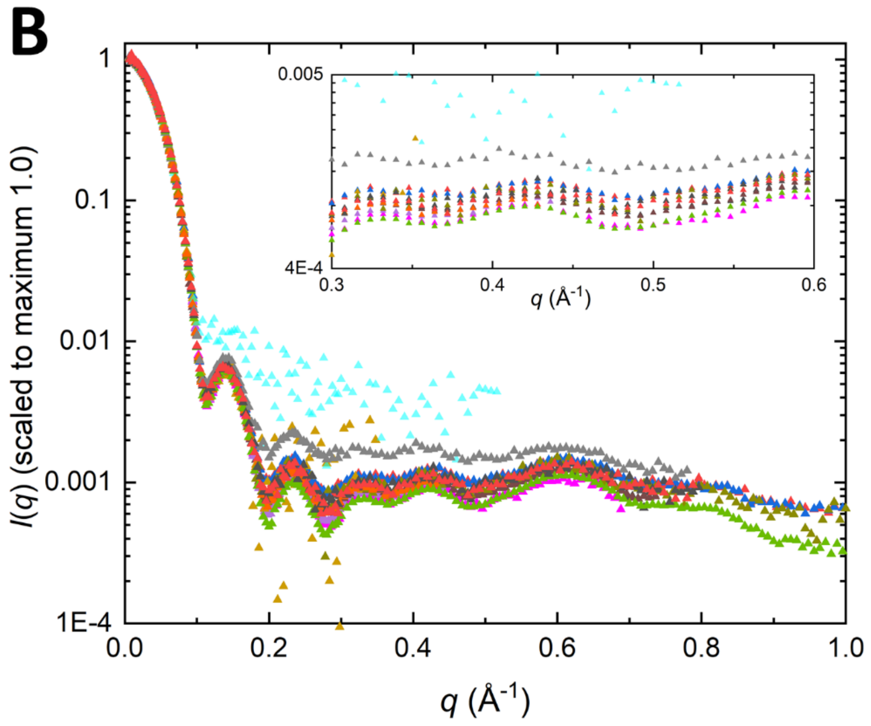

Figure S6 SAXS data as $I(q)$ vs q, Guinier plots and dimensionless Kratky plots for the data combined for the consensus profiles of RNase A (**A** and **B**), lysozyme (**C** and **D**), and xylanase (**E** and **F**).

Symbols are the individual contributing data after scaling and adjustment in *datcombine* with no filters applied. Lines are the consensus result with no filters (black) and with the outlier and error filters applied (red). Guinier plots, as inserts in **A**, **C** and **E** are for consensus results with no filters (black), error- and outlier-filters (red). Error bars as standard errors are shown for all data in Guinier plots, but for clarity only for *datcombine* results for the $I(q)$ vs q and Kratky plots (± 1 standard error propagated from errors provided with the original submitted data). Reference lines on the dimensionless Kratky plots are for qR_g= 1.73, $(qR_g)^2I(q)/I(0)$ = 1.1.

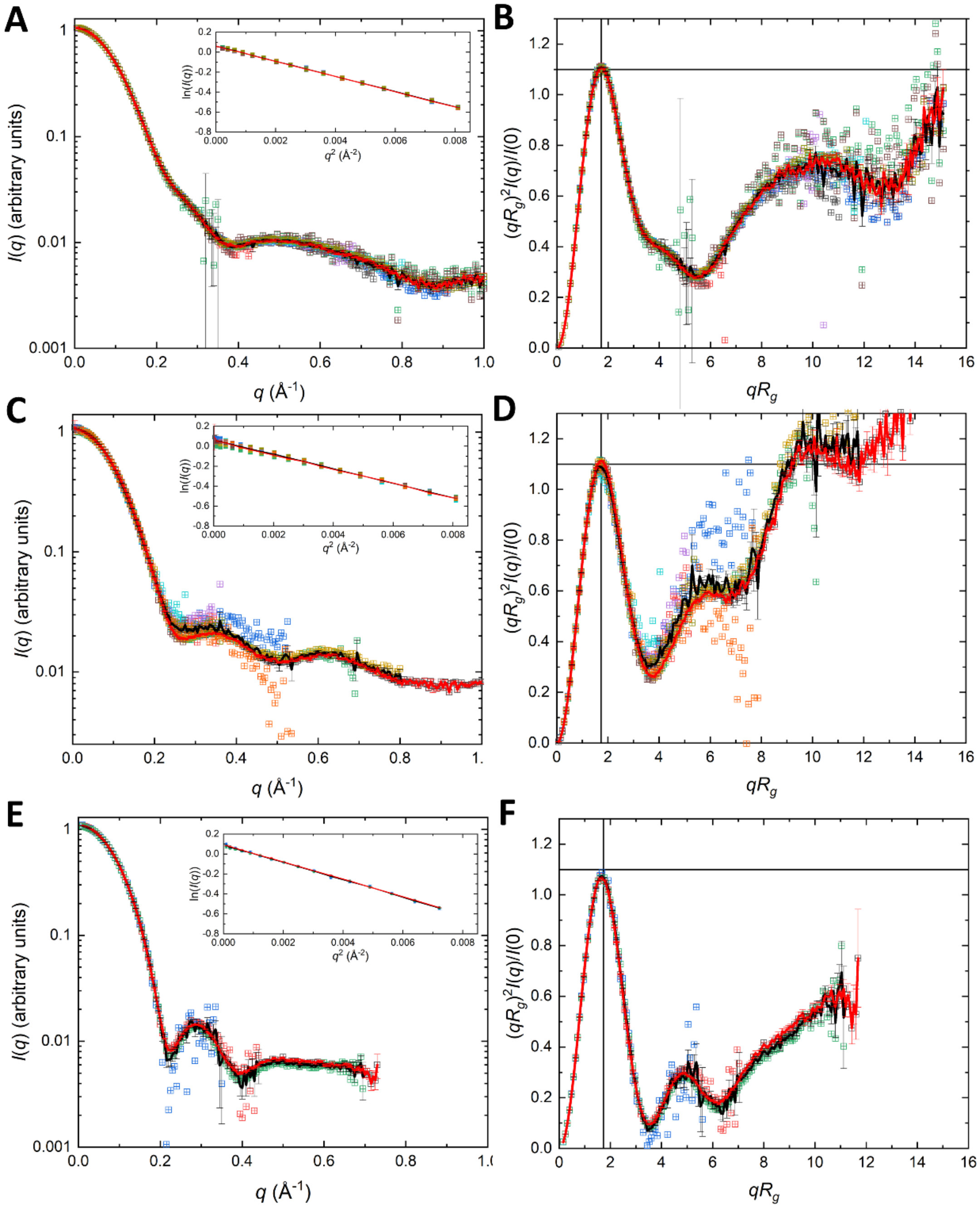

Figure S7 SAXS data as $I(q)$ vs q, Guinier plots, and dimensionless Kratky plots for the combined data sets for urate oxidase (**A** and **B**) and xylose isomerase (**C** and **D**).

Symbols are the individual contributing data after scaling and adjustment in *datcombine* with no filters applied. Lines are the consensus result with no filters (black) and with the outlier and error filters applied (red). Guinier plots, as inserts in **A** and **C** are for consensus results with no filters (black), error- and outlier-filters (red). Error bars as standard errors are shown for all data in Guinier plots, but for clarity only for *datcombine* results for the $I(q)$ vs q and Kratky plots (± 1 standard error propagated from errors provided with the original submitted data). Reference lines on the dimensionless Kratky plots are for qR_g= 1.73, $(qR_g)^2I(q)/I(0)$ = 1.1.

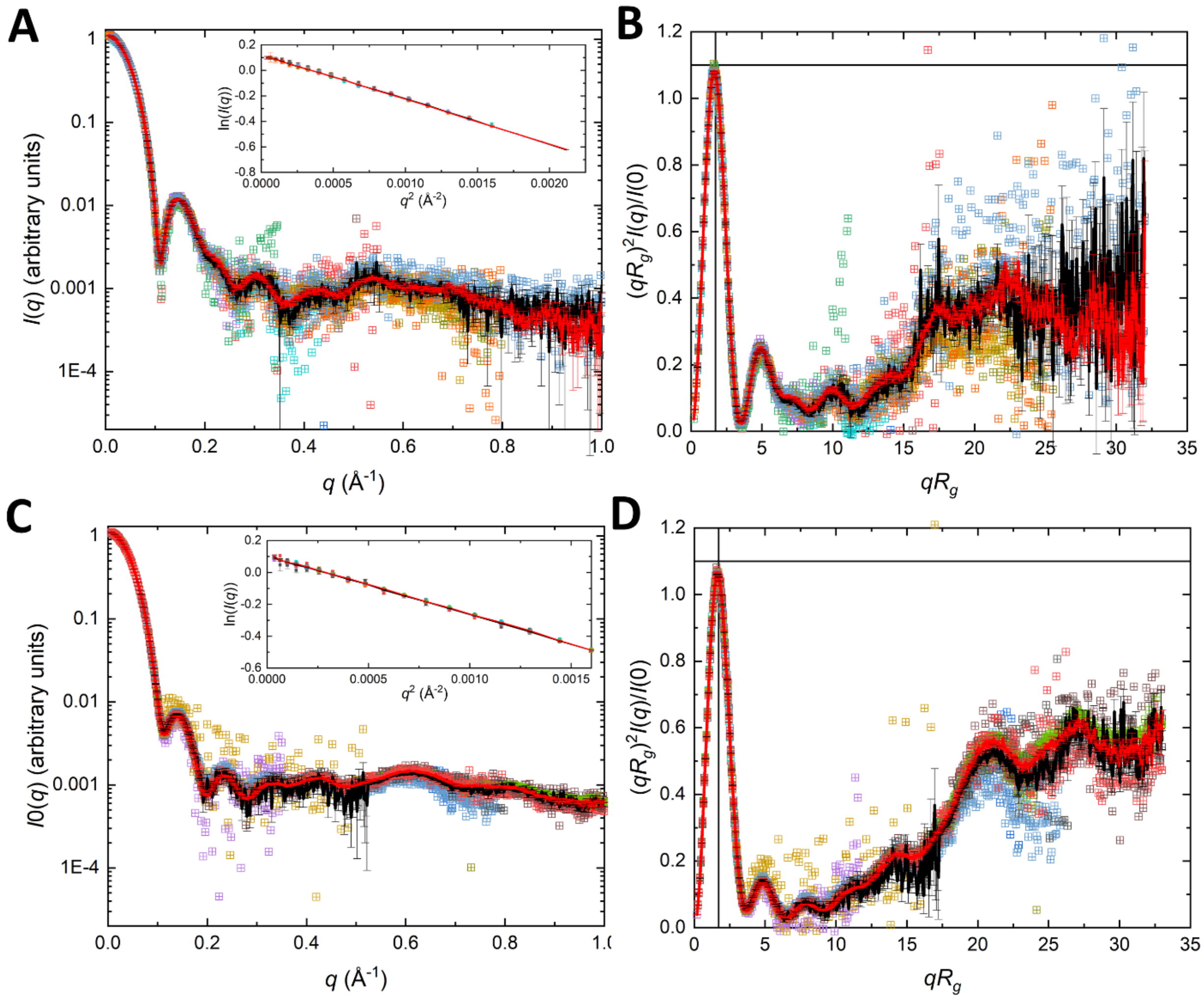

Figure S8 SANS data as $I(q)$ vs q profiles (symbols) and the *datcombine* result with no filters (black lines) and outlier- and error-filters applied (red lines) for RNase A (**A** and **B**), lysozyme (**C** and **D**), and xylanase (**E** and **F**) measured in D_2O (left panels) and H_2O (right panels).

Symbols are the individual contributing data after scaling and adjustment in *datcombine* with no filters applied, lines are the consensus result with no filters (black) and with the outlier and error filters applied (red). Guinier plots (with standard errors) are the consensus result (red squares) and the SEC-SANS measurement (blue squares) scaled. For clarity, only error bars for the consensus results are shown in the $I(q)$ vs q plots (± 1 standard error propagated from errors provided with the original submitted data). Note: for panel **E** the SEC-SANS and consensus result are identical in the Guinier region.

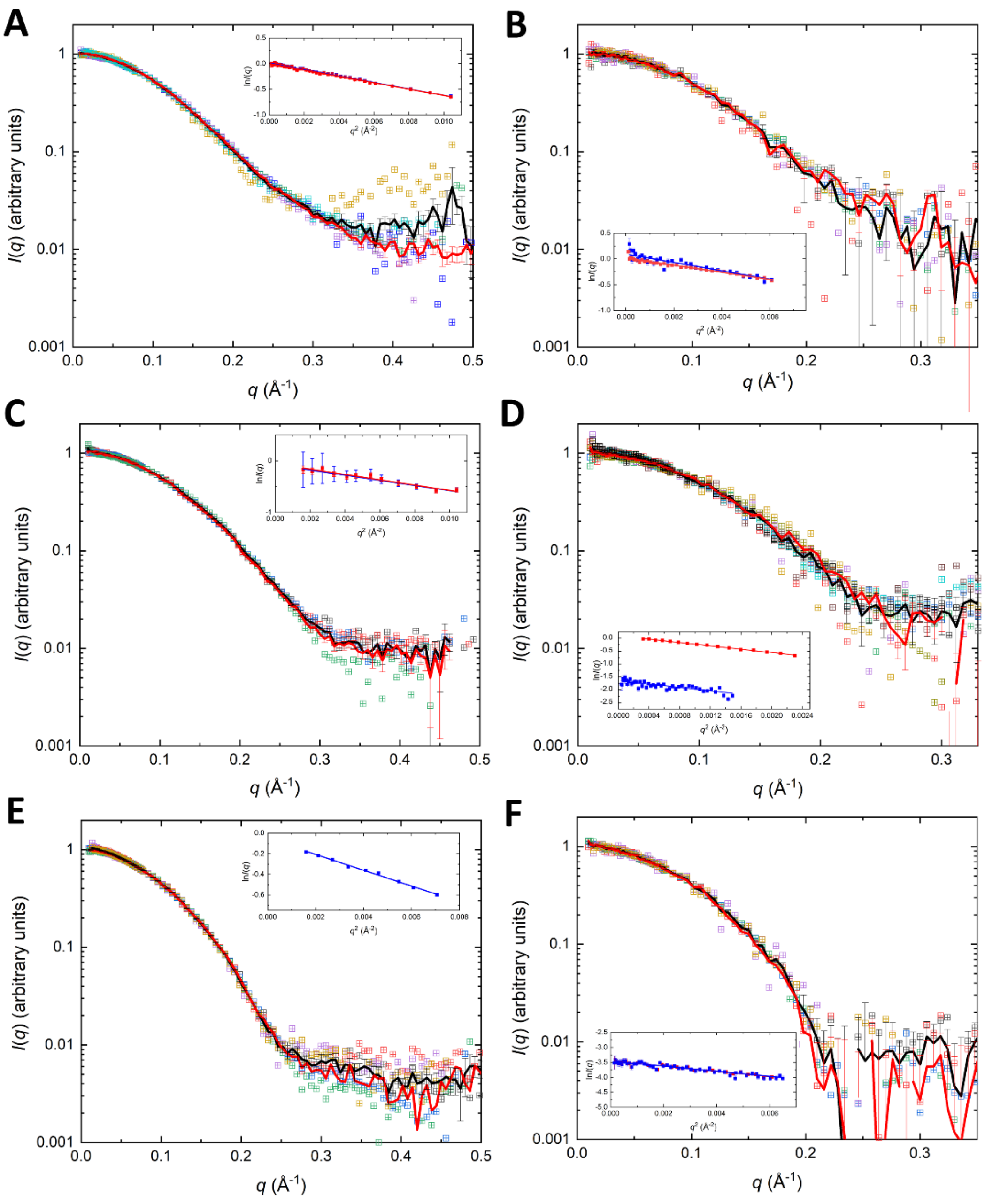

.

Figure S9 SANS data as $I(q)$ vs q profiles (symbols) and the *datcombine* result with no filters (black lines) and outlier- and error-filters applied (red lines) for urate oxidase (**A** and **B**), and xylose isomerase (**C** and **D**) measured in D_2O (left panels) and H_2O (right panels).

Symbols are the individual contributing data after scaling and adjustment in *datcombine* with no filters applied, lines are the consensus result with no filters (black) and with the outlier and error filters applied (red). Guinier plots (with standard errors) are the consensus result (red squares) and the SEC-SANS measurement (blue squares) scaled. For clarity, only error bars for the consensus results are shown in the *I*(*q*) vs *q* plots (± 1 standard error propagated from errors provided with the original submitted data).

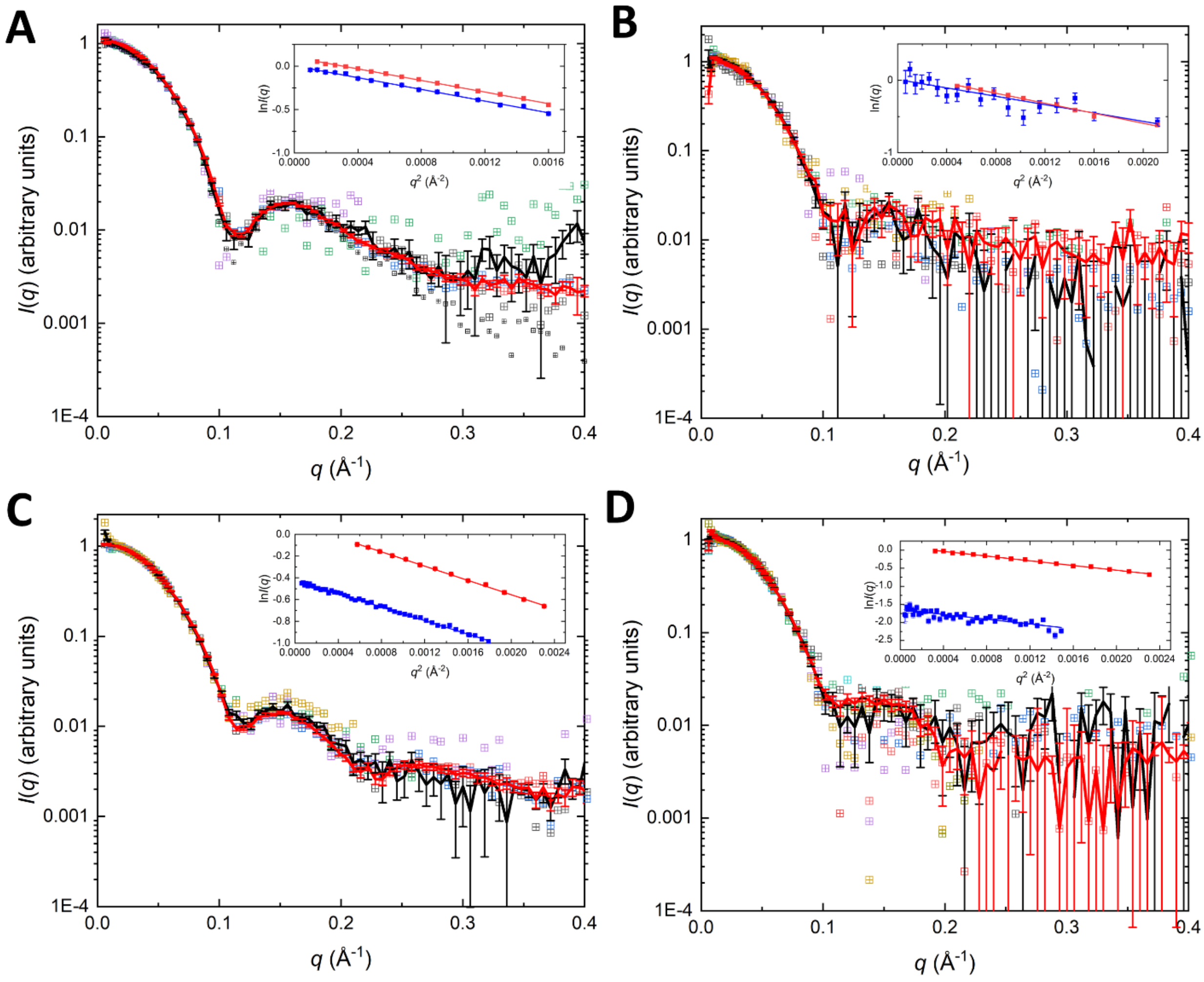

Figure S10 SEC-SANS data (blue filled squares) and the consensus profile as $I(q)$ vs q for RNase A in D_2O (**A**), lysozyme in D_2O (**B**), and xylanase (**C** and **D**) in D_2O and H_2O, respectively.

Error bars (± 1 standard error) for the consensus profiles are propagated from the errors in the original reduced data from contributors. Error bars in the SEC-SANS data are propagated counting statistics as provided by the data contributors.

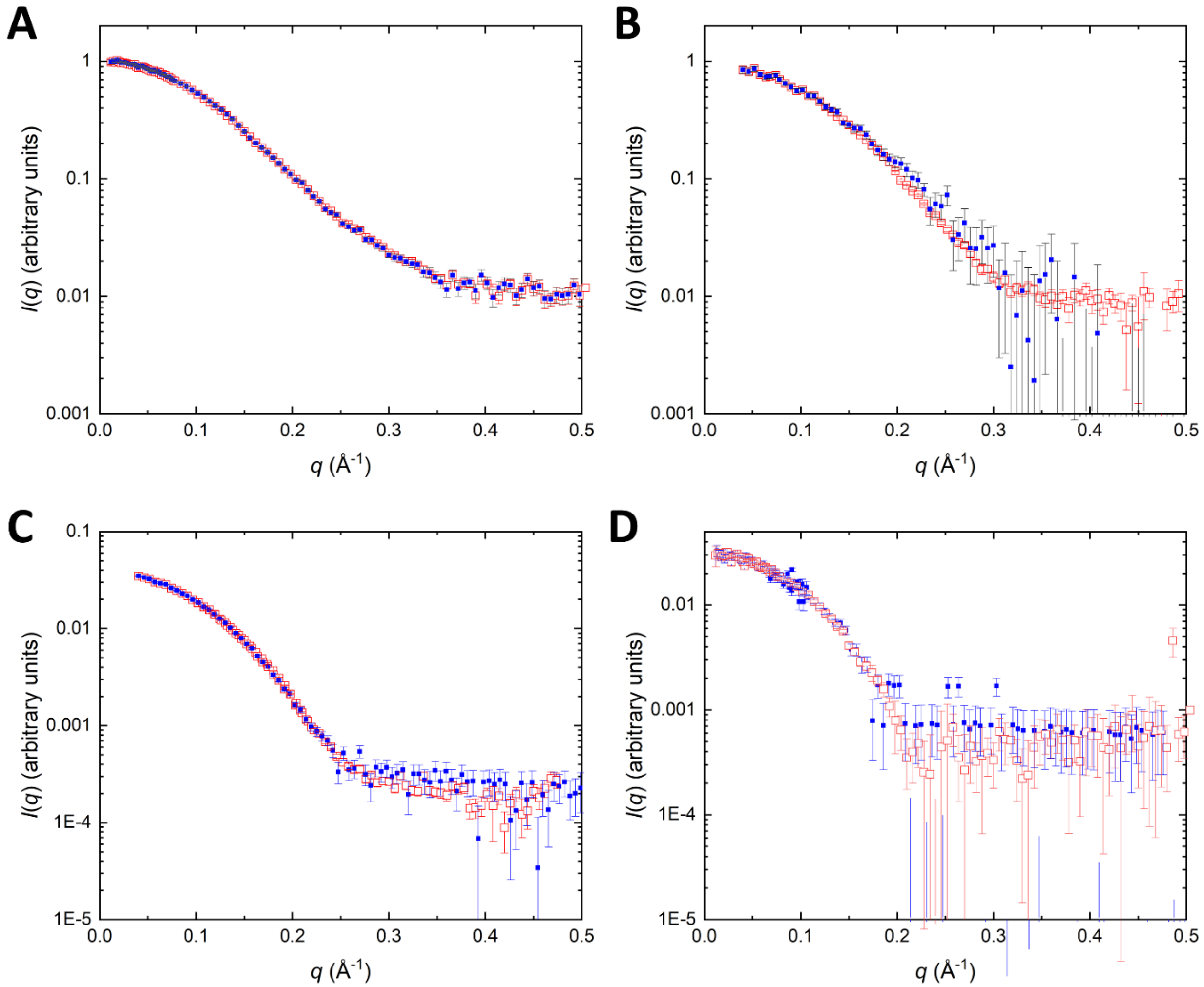

Figure S11 Error-weighted residual difference plots for the modelling calculations described in main text section **4. Comparisons with Prediction** for **A.** SAXS, **B.** SANS in D_2O and **C.** SANS in H_2O data. Colour coding is WAXSiS (black), CRYSOL (red), Pepsi-SAXS/SANS (blue), and FoxS (green). The broad oscillation observed for RNaseA SAXS data is consistent with a difference in the relative positions/orientations of domains for RNaseA potentially with some flexibility in solution compared to the crystal structure. The sharper, higher frequency features in the SAXS and SANS in D_2O residual plots that are most notable for urate oxidase and xylose isomerase are due to small differences in the positions and amplitudes of the minima and maxima arising from the approximately spherical nature of these scatterers.

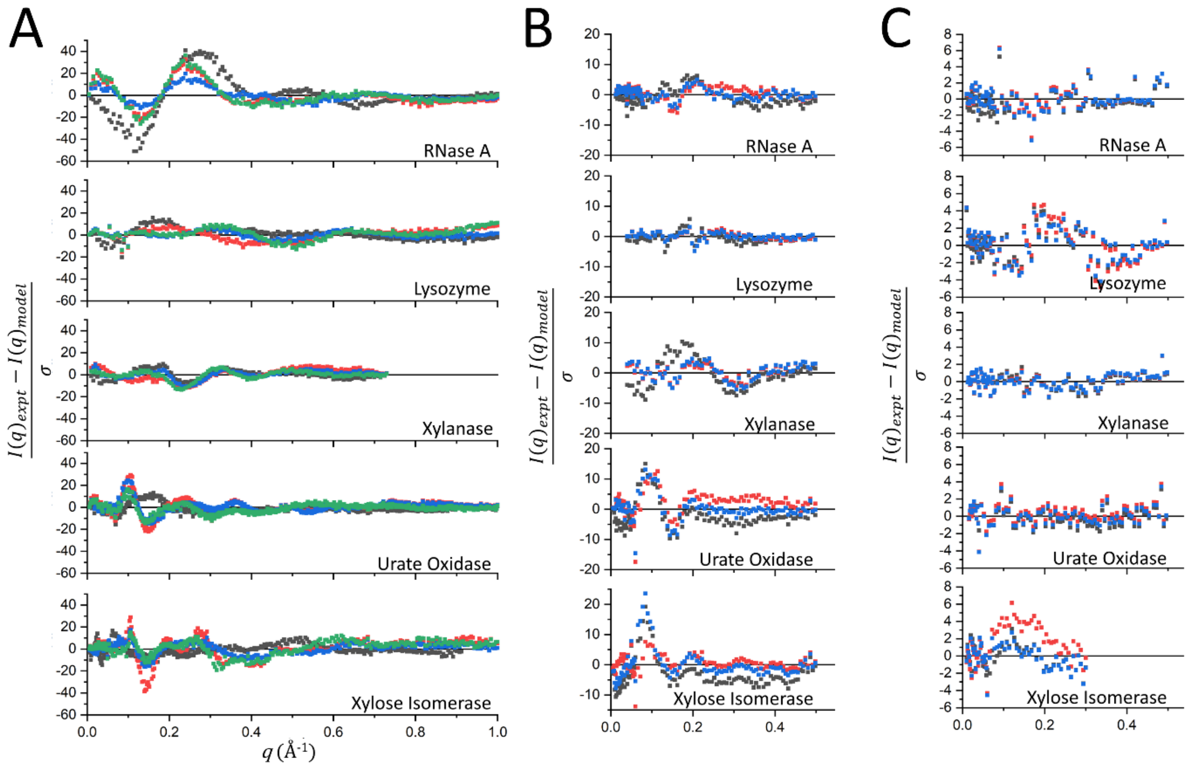

Figure S12 Data for (top to bottom traces) RNase A, lysozyme, xylanase, urate oxidase, and xylose isomerase from SEC-WAXS (black, measured at EMBL-P12 BioSAXS beam line, no lysozyme) and batch-WAXS (red, measured at the APS/12IDB beam line, no urate oxidase) as log-linear and log-log plots.

Error bars (± 1 standard error) are propagated counting statistics for the original reduced data from contributors.

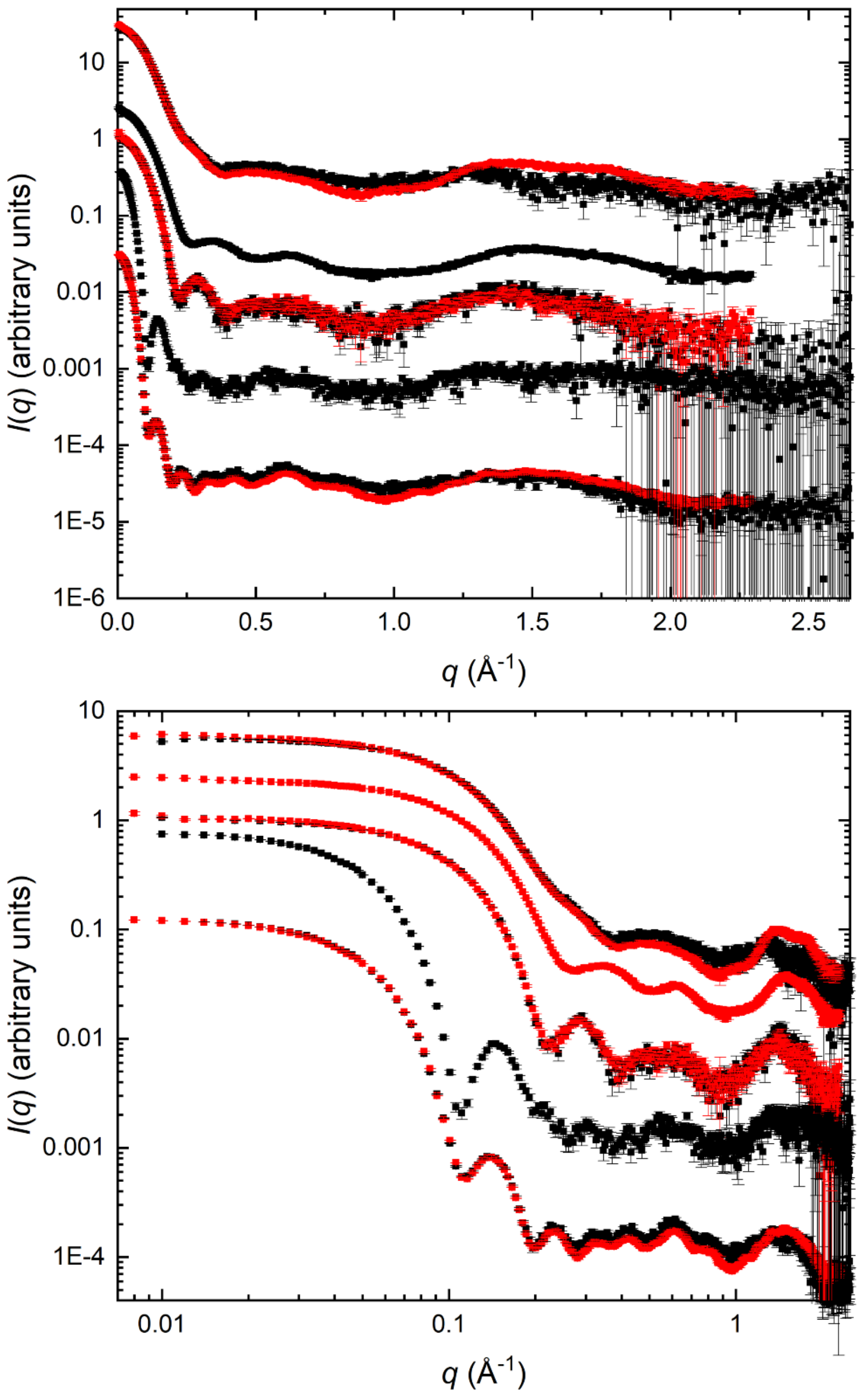

Table S1 Theoretical partial specific volume, $\bar{\vartheta}$, and hydration, α, values, and Porod volume (V_P) to molecular mass (m) ratio calculated using the method described in **S2**

Protein	m* Da	$\bar{\vartheta}$ $cm^3.g^{-1}$ at 20°C	α $g.g^{-1}$	V_P/m
RNaseA	13,690	0.710	0.36	1.48
Lysozyme	14,313	0.716	0.323	1.45
Xylanase	20,844	0.712	0.295	1.43
Urate oxidase	136,303	0.735	0.375	1.53
Xylose isomerase	172,910	0.727	0.385	1.52

*m values based on chemical composition, see main text, **Table 1**

Table S2 UniProt Sequences with modifications and ligands

Protein, Uniprot ID	Red indicates amino acids not included in the construct measured by SAS.
RNase A, P61823	MALKSLVLLSLLVLVLLLVRVQPSLGKETAAAKFERQHMDSSTSAASSSNYCNQMMKS<br>RNLTKDRCKPVNTFVHESLADVQAVCSQKNVACKNGQTNCYQSYSTMSITDCRETGSS<br>KYPNCAYKTTQANKHIIVACEGNPYVPVHFDASV
Lysozyme, P00698	MRSLLILVLCFLPLAALGKVFGRCELAAAMKRHGLDNYRGYSLGNWVCAAKFESNFNT<br>QATNRNTDGSTDYGILQINSRWWCNDGRTPGSRNLCNIPCSALLSSDITASVNCAKKIVS<br>DGNGMNAWVAWRNRCKGTDVQAWIRGCRL
Xylanase, F8W699	ETIQPGTGYNNGYFYSYWNDGHGGVTYTNGPGGQFSVNWSNSGNFVGGKGWQPGTK<br>NKVINFSGSYNPNGNSYLSVYGWSRNPLIEYYIVENFGTYNPSTGATKLGEVTSDGSVY<br>DIYRTQRVNQPSIIGTATFYQYWSVRRNHRSSGSVNTANHFNAWAQQGLTLGTMDYQ<br>IVAVEGYFSSGSASITVS
Urate oxidase, Q00511	MSAVKAARYGKDNVRVYKVHKDEKTGVQTVYEMTVCVLLEGEIETSYTKADNSVIVA<br>TDSIKNTIYITAKQNPVTPPELFGSILGTHFIEKYNHIHAAHVNIVCHRWTRMDIDGKPHP<br>HSFIRDSEEKRNVQVDVVEGKGIDIKSSLSGLTVLKSTNSQFWGFLRDEYTTLKETWDRI<br>LSTDVDATWQWKNFSGLQEVRSHVPKFDATWATAREVTLKTFAEDNSASVQATMYK<br>MAEQILARQQLIETVEYSLPNKHYFEIDLSWHKGLQNTGKNAEVFAPQSDPNGLIKCTV<br>GRSSLKSKL, N-terminal Ser is acetylated, bound ligand 8-azaxanthine: $C_4H_3N_5O_2$
Xylose isomerase, P24300	MNYQPTPEDRFTFGLWTVGWQGRDPFGDATRRALDPVESVRRLAELGAHGVTFHDD<br>DLIPFGSSDSEREEHVKRFRQALDDTGMKVPMATTNLFTHPVFKDGGFTANDRDVRR<br>YALRKTIRNIDLAVELGAETYVAWGGREGAESGGAKDVRDALDRMKEAFDLLGEYVTS<br>QGYDIRFAIEPKPNEPRGDILLPTVGHALAFIERLERPELYGVNPEVGHEQMAGLNFPH<br>GIAQALWAGKLFHIDLNGQNGIKYDQDLRFGAGDLRAAFWLVDLLESAGYSGPRHFDF<br>KPPRTEDFDGVWASAAGCMRNYLILKERAAAFRADPEVQEALRASRLDELARPTAAD<br>GLQALLDDRSAFEEFDVDAAAARGMAFERLDQLAMDHLLGARG, bound Mg2+

Table S3 Data Acquisition and reduction details for each contributing facility

**SAXS Data**						
**Advanced Light Source - SIBYLS**						
Experiment dates: 7 Jan. 2020						
Special sample conditions						
Protein		RNaseA	Lysozyme	Xylanase	Urate oxidase	Xylose isomerase
For SEC-SAXS	Injection volume (µL)	50	50	50	50	50
	Loading concentration (mg/mL)	18	11.2	11.7	4.7	19.8
	Flowrate (mL/min)	0.4	0.4	0.4	0.4	0.4
Batch measurement concentrations (mg/mL)		18.1, 10.2, 6.2, 3.9	11.0, 8.91, 6.85, 4.80	12.13, 7.96, 6.82, 4.17	4.53, 3.96, 2.87, 2.12	19.6, 10.4, 6.1, 4.28
Notes		No azide was added prior to SAXS measurement				
SAS data collection parameters						
Source, instrument and description or reference		SIBYLS beamline, Advanced Light Source, Lawrence Berkeley National Laboratory.<br>Detector: Pilatus3 2M pixel array detector<br>Beamline citations: (Dyer *et al.*, 2014, Classen *et al.*, 2013)				
Wavelength (Å)		1.2155 Å				
Beam geometry (size, sample-to-detector distance)		Beam size: 1 mm horizontal, 0.5 mm vertical at sample.<br>Beam size: 100 x 100 µm at detector.<br>Sample to detector distance: 2.081 m<br>Flux on sample: 10^{12} photons/second				
q-measurement range (Å^{-1} or nm^{-1})		0.009 – 0.37 Å^{-1}				
Absolute scaling method		Lysozyme standard				
Basis for normalization to constant counts		0.02243 detector/diode counts to cm^{-1} scale				
Method for monitoring radiation damage, X-ray dose where relevant		Web tool for frame sliced data<br>sibyls.als.lbl.gov/ran				
Exposure time, number of exposures		High throughput (HT)-SAXS: Total 10 seconds, framing at 0.2second intervals<br>SEC-SAXS: Total 1200 seconds, framing at 2 second intervals				
Sample configuration including path length and flow rate where relevant		For HT and SEC: 1mm sample thickness<br>For SEC: Shodex 802.5 column, flow rate 0.4 mL/min				
Sample temperature (ºC)		20 ºC				
Software employed for SAS data reduction, analysis, and interpretation						
SAS data reduction to sample–solvent scattering		Image processing and signal normalization was done with in-house software.<br>SEC-SAXS data-buffer subtraction and merging were done with the older version of SCATTER (scatter 3)<br>(https://bl1231.als.lbl.gov/scatter/)				
**Advanced Photon Source – 12-ID-B**						
Experiment dates: 13 – 16 Dec. 2019						
Special Sample Conditions						
Protein		RNaseA	Lysozyme	Xylanase	Urate oxidase	Xylose isomerase
Batch measurement concentrations (mg/mL)		1.0, 2.5, 5.0	1.0, 2.5, 5.0	0.8, 1.5	n.a.	1.0, 2,7 5.0 (D_2O), 1.0, 5.0 (H_2O)

Notes	Azide was added to samples prior to SAXS measurement
**SAS data collection parameters**	
Source, instrument and description or reference	APS Undulator 2.7, APS Beamline 12-ID-B; https://12idb.xray.aps.anl.gov/BioSAXSWAXS.html; Detectors: Pilatus 2M (SAXS), Pilatus 300K (WAXS)
Wavelength (Å)	0.9123Å
Beam geometry (size, sample-to-detector distance)	0.10 mm (vertical) x 0.14 mm (horizontal); S-D: 2.0 m for SAXS, 0.45 m for WAXS
q-measurement range ($Å^{-1}$ or nm^{-1})	0.005 $Å^{-1}$ to 0.88 $Å^{-1}$ SAXS; 0.84 $Å^{-1}$ to 2.30 $Å^{-1}$ WAXS
Absolute scaling method	relative to water (1.632 e^{-2} cm^{-2} at 20°C)
Basis for normalization to constant counts	Transmitted intensity measured via a pin diode
Method for monitoring radiation damage, X-ray dose where relevant	Frame to frame consistency
Exposure time, number of exposures	0.5 – 1.0 s taking every 2 seconds, 40 frames
Sample configuration including path length and flow rate where relevant	1.5 mm diameter cylindrical capillary, 0.6 mL/min flow rate
Sample temperature (ºC)	20
**Software employed for SAS data reduction, analysis, and interpretation**	
SAS data reduction to sample–solvent scattering	Beamline developed Matlab package (qCalibration2 & SAXSLee)
**Advanced Photon Source - BioCAT**	
Experiment dates: 2019/07/14 (RNAse A, xylanase), 2019/07/17 (lysozyme, urate oxidase), 2019/08/01 (xylose isomerase)	
**Special Sample Conditions**	

Protein		RNaseA	Lysozyme bio, RR sample	Xylanase	Urate oxidase	Xylose isomerase
SEC-SAXS	Injection volume (µL)	250	250, 250	250	250	100
	Loading concentration (mg/mL)	10	15, 20	10	10	25
	Flowrate (mL/min)	0.8	0.8, 0.7	0.8	0.8	0.7

Notes	2 lysozymes measured: Round robin (RR) supplied lysozyme Locally sourced (bio) lysozyme (Lysozyme, Chicken Egg White, Ultrapure, Fisher Scientific AAJ1864514 (Affymetrix)) No azide was added prior to SEC-SAXS measurement.
**SAS data collection parameters**	
Source, instrument and description or reference	BioCAT facility at the Advanced Photon Source beamline 18ID Detector: Pilatus3 X 1M (Dectris) detector
Wavelength (Å)	1.033
Beam geometry (size, sample-to-detector distance)	Size: 150 (horizontal) x 25 (vertical) μm^2 focused at the detector SDD: 3.686 m
q-measurement range ($Å^{-1}$)	0.0043 – 0.3522
Absolute scaling method	Glassy carbon
Basis for normalization to constant counts	To transmitted intensity by beam-stop counter
Method for monitoring radiation damage, X-ray dose where relevant	Data frame-by-frame comparison using CORMAP algorithm (Franke *et al.*, 2015)
Exposure time, number of exposures	0.5 s exposure time with a 1 s total exposure period (0.5 s on, 0.5 s off) of entire SEC elution.
Sample configuration including path length and flow rate where relevant	SEC-SAXS with a Superdex 200 Increase 10/300 column and sheath-flow cell (Kirby *et al.*, 2016), effective sample path length 0.542 mm
Sample temperature (ºC)	23

Software employed for SAS data reduction, analysis, and interpretation						
SAS data reduction to sample–solvent scattering		Radial averaging; normalization, frame comparison, averaging, subtraction, and baseline correction done using BioXTAS RAW 1.6.0 (Hopkins *et al.*, 2017).				
**Australian Synchrotron**						
Experiment dates: 21 Nov. 2019						
Special Sample conditions						
Protein		RNaseA	Lysozyme	Xylanase	Urate oxidase	Xylose isomerase
SEC-SAXS	Injection volume (µL)	50	50	50	50	50
	Loading concentration (mg/mL)	5	5	5	6	2
	Flowrate (mL/min)	0.4	0.4	0.4	0.4	0.4
Batch measurement concentrations (mg/mL)		2.0, 4.0, 2.0, 1.0	6.0, 3.0, 1.5, 0.75	14.0, 7.0, 3.5, 1.75	n.a.	7.2, 3.6, 1.8, 0.9
Notes		Azide was added to samples prior to SAXS measurement				
SAS data collection parameters						
Source, instrument and description or reference		Australian Synchrotron SAXS/WAXS, 12 keV (Kirby *et al.*, 2013)<br>Detectors: Pilatus3-2M (Dectris)				
Wavelength (Å)		1.036 Å				
Beam geometry (size, sample-to-detector distance)		250 x 500 µm, 2.5 m sample to detector				
q-measurement range (Å^{-1} or nm^{-1})		0.0074 – 0.698 Å^{-1}				
Absolute scaling method		Water				
Basis for normalization to constant counts		Beamstop counter (transmission)				
Method for monitoring radiation damage, X-ray dose where relevant		Guinier analysis, conversion of beamstop count rate to flux.				
Exposure time, number of exposures		1s, batch ~ 40 exposures, SEC-SAXS: ~8 exposures depending on peak)				
Sample configuration including path length and flow rate where relevant		Batch mode – conventional Quartz capillary, in vacuum, nominal 1mm pathlength, flowrate ~4 µL/s;<br>SEC-SAX mode – Coflow, 2:1 flow ratio (sample flow 0.4 mL/min, in cell flow of 0.8 mL/min), 1 mm Quartz capillary				
Sample temperature (ºC)		10				
Software employed for SAS data reduction, analysis, and interpretation						
SAS data reduction to sample–solvent scattering		ScatterBrain v 2.82 (http://archive.synchrotron.org.au/aussyncbeamlines/saxswaxs/software-saxswaxs#:~:text=scatterBrain%20is%20a%20software%20package,at%20ChemMatCARS%20at%20the%20APS.)				
**Cornell High Energy Synchrotron Source (CHESS) – ID7a**						
Experiment dates: 12-19 Aug. 2019						
Special Sample Conditions						
Protein		RNaseA	Lysozyme	Xylanase	Urate oxidase	Xylose isomerase
SEC-SAXS	Injection volume (µL)	n.a	n.a.	n.a.	100	n.a.
	Loading concentration (mg/mL)	n.a	n.a.	n.a.	10	n.a.
	Flowrate (mL/min)	n.a	n.a.	n.a.	0.5	n.a.
For Batch SAXS, concentrations (mg/mL)		RNaseA	Lysozyme	Xylanase	Urate oxidase	Xylose isomerase

	17.13, 5.17	5.22	12.51, 4.17, 2.08	1.45, 2.9	13.5, 0.93, 0.46
Notes	No azide was added to samples prior to SAXS measurement<br>A locally sourced sample (Chicken Egg White L-7651 Lot 072KZ062) was measured as the round robin sample was brown tinge. The round robin sample was also measured an had a concentration of 6.0 mg/mL estimated from *I*(0) comparison with the locally sourced sample.				
SAS data collection parameters					
Source, instrument and description or reference	Cornell High Energy Synchrotron Source, ID7a (https://www.chess.cornell.edu/users/biosaxs-hp-bio-beamline)<br>Detector: Eiger 4M (Dectris)				
Wavelength (Å), bandwidth, flux	1.260 Å (9.835 keV) 1.5% bandwidth, 2.8×10^{12} ph/s				
Beam geometry (size, sample-to-detector distance)	0.25 mm x 0.25 mm, SAXS: 1514 mm, WAXS: 450 mm				
q-measurement range ($Å^{-1}$ or nm^{-1})	SAXS: 0.009-0.275 $Å^{-1}$ WAXS: 0.232-0.745 $Å^{-1}$				
Absolute scaling method	water – empty (BioXTAS RAW)				
Basis for normalization to constant counts	beamstop diode (Si)				
Method for monitoring radiation damage, X-ray dose where relevant	CorMap test, pval threshold 0.01				
Exposure time, number of exposures	0.1 s, 20 exposures				
Sample configuration including path length and flow rate where relevant	1.5 mm ID quartz glass capillary, 10 µm wall thickness, oscillating flow				
Sample temperature (ºC)	21.6				
Software employed for SAS data reduction, analysis, and interpretation					
SAS data reduction to sample–solvent scattering	BioXTAS RAW version 1.6.0				
**Diamond Light Source - B21**					
Experiment dates: 18 July 2019					
Special Sample Conditions					
Protein	RNaseA	Lysozyme	Xylanase	Urate oxidase	Xylose isomerase
For SEC-SAXS: Injection volume (µL)	45	45	45	45	45
For SEC-SAXS: Loading concentration (mg/mL)	~10	~10	~10	~10	~10
For SEC-SAXS: Flowrate (mL/min)	0.16	0.16	0.16	0.16	0.16
Starting batch measurement concentrations (mg/mL), for each protein's 7-serial dilution series	9.2	27.6	31.8	6.8	21.5
Notes	Azide was added to samples prior to SAXS measurement				
SAS data collection parameters					
Source, instrument and description or reference	DLS B21 (Cowieson *et al.*, 2020)<br>Detector: Eiger 4M (Dectris)				
Wavelength (Å)	0.954				
Beam geometry (size, sample-to-detector distance)	2696 mm (at sample beam is 1.2 x 0.9 mm at detector it is a ~60 µm Gaussian spot FWHM)				
q-measurement range ($Å^{-1}$ or nm^{-1})	0.0032 to 0.44 $Å^{-1}$				
Absolute scaling method	Water scatter				
Basis for normalization to constant counts	Integrating beamstop diode				
Method for monitoring radiation damage, X-ray dose where relevant	Multiple short exposures are compared for changes and averaged				
Exposure time, number of exposures	20 x 1 s exposures				

Sample configuration including path length and flow rate where relevant		1.5 mm capillary flowing at 1 uL/s during collection for batch SAXS<br>Shodex KW403 column used for SEC-SAXS (0.16 mL/min)				
Sample temperature (ºC)		20				
Software employed for SAS data reduction, analysis, and interpretation						
SAS data reduction to sample–solvent scattering		Data Analysis WorkbeNch, DAWN (Basham *et al.*, 2015)				
**NIST/IBBR, SAXSLab Ganesha Instrument**						
Experiment dates: 26 Sep. – 15 Oct. 2019						
Special Sample Conditions						
Protein		RNaseA	Lysozyme	Xylanase	Urate oxidase	Xylose isomerase
Batch measurement concentrations		2, 2.5, 5.0, 10.0	2.5, 5.0, 10.0	2.1, 4.2	0.7, 1.4 (in H_2O and D_2O)	0.5, 1.0, 3.0 (in H_2O and D_2O)
Notes		Azide was added to samples prior to SAXS measurement				
SAS data collection parameters						
Source, instrument and description or reference		Rigaku Micromax 007HF rotating anode source, SAXSLab Ganesha, Pilatus 300K detector				
Wavelength (Å)		1.5418				
Beam geometry (size, sample-to-detector distance)		0.4 mm / 1.76 m SAXS; 0.8 mm / 0.36 m WAXS				
q-measurement range (Å^{-1} or nm^{-1})		0.005Å^{-1} to 0.15Å^{-1} SAXS; 0.035Å^{-1} to 0.8Å^{-1} WAXS				
Absolute scaling method		Water $I(0)$ measurement at 20ºC				
Basis for normalization to constant counts		Transmitted intensity measured via a pin diode				
Method for monitoring radiation damage, X-ray dose where relevant		Frame/frame consistency				
Exposure time, number of exposures		900 sec, 16 frames for SAXS, WAXS				
Sample configuration including path length and flow rate where relevant		Cylindrical capillary, static				
Sample temperature (ºC)		25				
Software employed for SAS data reduction, analysis, and interpretation						
SAS data reduction to sample–solvent scattering.		BioXTAS RAW 1.1.0 (Hopkins *et al.*, 2017)				
**Petra III, P12 BioSAXS**						
Experiment dates:26 – 28 Nov. – 1 Dec. 2019						
Special Sample Conditions						
Protein		RNaseA	Lysozyme	Xylanase	Urate oxidase	Xylose isomerase
For SEC-SAXS	Injection volume (µL)	75	n.a.	75	82	75
	Loading concentration (mg/mL)	8	n.a.	11	11	7.6
	Flowrate (mL/min)	0.6	n.a.	0.6	0.6	0.6
For SEC-WAXS	Injection volume (µL)	75.	n.a.	75	100	100
	Loading concentration (mg/mL)	9.7	n.a.	8.6	5.9	10.3
	Flowrate (mL/min)	0.6	n.a.	0.6	0.6	0.7
Batch measurement concentrations		1.8, 3.6, 7.2	n.a.	1.39, 2.78, 5.57	5.91	1.44, 2.89, 5.77
Notes		All suggested buffers supplemented with 1% glycerol, except in the case of SEC-SAXS measurement for RNaseA and xylanase				

	where buffers were substituted with 50 mM HEPES, 150 mM KCl, 3% glycerol to avoid capillary fouling.<br>No azide was added prior to SAXS measurements				
SAS data collection parameters					
Source, instrument and description or reference	U29 PETRAIII undulator @ DESY, Hamburg, Germany;<br>P12 BioSAXS Beamline, on U29 PETRAIII undulator, Pilatus 6M detector (Blanchet *et al.*, 2015)<br>*BECQUEREL* control software (Hajizadeh *et al.*, 2018)				
Wavelength (Å)	SEC-SAXS and Batch SAXS: 1.24 (10 keV)<br>SEC-WAXS: 0.62 (20 keV)				
Beam geometry (size, sample-to-detector distance)	SEC-SAXS and Batch SAXS: (Beam size: 200x300 μm^2, Sample-Detector 3 m)<br>SEC-WAXS: (Beam size: 200x300 μm^2, Sample-Detector 1.5 m)				
q-measurement range ($Å^{-1}$ or nm^{-1})	SAXS: 0.0025 $Å^{-1}$ to 0.7321$Å^{-1}$<br>WAXS: 0.0086 $Å^{-1}$ to 2.6548 $Å^{-1}$				
Absolute scaling method	Water scattering at 20°C				
Basis for normalization to constant counts	Transmitted beam intensity, via PIN diode in beamstop				
Method for monitoring radiation damage, X-ray dose where relevant	Batch SAXS: Comparison of data frames using CorMAP				
Exposure time, number of exposures	SEC-SAXS: 2400 x 1 s throughout SEC elution<br>Batch SAXS: samples 40 x 100 ms frames, buffers 2 blocks of 40 x 100ms<br>SEC-WAXS:2100 x 1 s throughout SEC elution				
Sample configuration including path length and flow rate where relevant	SEC-SAXS:<br>*RNaseA and xylanase*: column S75 Increase 10/300, 0.6 mL/min, measurement cell 1.0 mm capillary.<br>*Xylose isomerase and urate oxidase*: column S200 Increase 10/300, 0.6 mL/min, measurement cell 1.0 mm capillary.<br>Batch SAXS: measurement cell 1.0 mm capillary<br>SEC-WAXS:<br>*RNaseA and xylanase*: column S75 Increase 10/300, 0.6 mL/min, sample cell 1.0 mm capillary.<br>*Xylose isomerase and urate oxidase*: column S200 Increase 10/300, 0.7 mL/min, measurement cell 1.8 mm capillary.				
Sample temperature (ºC)	20				
Software employed for SAS data reduction, analysis, and interpretation					
SAS data reduction to sample–solvent scattering	*SASFLOW* automated 2D-1D data reduction and processing; (Franke *et al.*, 2012). SEC-SANS data were processed using CHROMIXS (Panjkovich & Svergun, 2018) or US-SOMO (Brookes *et al.*, 2016)				
**Shanghai Synchrotron Radiation Facility – BL192U**					
Experiment dates: 23 July 2019 and 17 Dec. 2019					
Special Sample Conditions					
Protein	RNaseA	Lysozyme	Xylanase	Urate oxidase	Xylose isomerase
For SEC-SAXS: Injection volume (µL)	100	100	100	100	100
For SEC-SAXS: Loading concentration (mg/mL)	8.23	13.4	13.	5.5	22.8
For SEC-SAXS: Flowrate (mL/min)	0.5	0.5	0.5	0.5	0.5
Batch measurement concentrations (mg/mL)	2.06, 4.11, 8.23	3.35, 6.70, 13.40	3.35, 6.70, 13.40	1.00, 2.50, 5.50	2.20, 5.69, 11.39

Notes		No azide was added to samples prior to SAXS measurements				
SAS data collection parameters						
Source, instrument and description or reference		BL19U2 BioSAXS Beamline, National Facility for Protein Science Shanghai, with two detectors inline: Pilatus2M (SAXS), Pilatus 300 k-w (WAXS)<br>Refs: (Li *et al.*, 2016, Liu *et al.*, 2018, Wu *et al.*, 2020)				
Wavelength (Å)		1.03 (12 keV)				
Beam geometry (size, sample-to-detector distance)		340 µm x 60 µm (horizontal x vertical), 2.415 m				
q-measurement range (Å^{-1} or nm^{-1})		0.0087 – 0.526 Å^{-1}				
Absolute scaling method		setting absolute scale with water				
Basis for normalization to constant counts		Transmitted intensity measured via a pin diode integrated in beamstop				
Method for monitoring radiation damage, X-ray dose where relevant		SAXS data were collected as continuous serial exposures and scattering profiles for the set of frames were compared using CorMap to monitor the radiation damage				
Exposure time, number of exposures		Batch mode: 1 s exposure, 20 frames;<br>SEC-SAXS mode: 1.5 s exposure, 1500 frames				
Sample configuration including path length and flow rate where relevant		flow cell made of a cylindrical quartz capillary with a diameter of 1.5 mm and a wall of 10 µm. Sample was oscillated up and down during exposures.				
Sample temperature (ºC)		4				
Software employed for SAS data reduction, analysis, and interpretation						
SAS data reduction to sample–solvent scattering, and extrapolation, merging, desmearing *etc*. as relevant		Primary scattering data reduction was done using SAS-cam 1.0.1 (Wu *et al.*, 2020). Further merging and modelling was done with BioXTAS RAW 1.6.0 and ATSAS 2.8.1.				
**Synchrotron SOLEIL - SWING**						
Experiment dates: 9 – 13 July, 2019						
Special Sample Conditions						
Protein		RNaseA	lysozyme	xylanase	Urate oxidase	Xylose isomerase
For SEC-SAXS	Injection volume (µL)	50	50	50	50	50
	Loading concentration, (mg/mL)	21.9	9.0	16.5	5.2	23.0
	flow rate (mL/min)	0.3	0.3	0.3	0.3	0.3
Concentrations for batch mode (mg/mL) for 1 and 2 m sample – detector set ups	1m	5.5, 10.7	4.5, 9.0	2.9, 7.8	1.8, 3.9	5.7, 15.1
	2 m	5.7, 10.3	4.5, 9.0	3.0, 8.1	1.8, 3.9	7.7, 14.4
Notes		No azide was added to samples prior to SAXS measurement				
SAS data collection parameters						
Source, instrument and description or reference		SOLEIL/SWING, U20 in-vacuum undulator, instrument (https://www.synchrotron-soleil.fr/en/beamlines/swing)<br>Reference (A. Thureau *et al.*, 2021)<br>Detectors: SAXS, EigerX4M (Dectris); WAXS, Merlin (Quantum Detector)				
Wavelength (Å)		1.033				
Beam geometry (size, sample-to-detector distance)		400x200 µm^2. Distance 1m (WAXS) and 2m (SAXS)				
q-measurement range (Å^{-1} or nm^{-1})		0.0070 – 1.00 (1 m) and 0.0032-0.52 (2 m)				
Absolute scaling method		Water				
Basis for normalization to constant counts		Active beamstop: diamond-based diode				
Method for monitoring radiation damage		Monitoring successive data frames for any changes				

Exposure time, number of exposures	0.99 s (0.01 s dead time). 40 frames for batch<br>180 frames + 600 frames for HPLC (buffer + sample)
Sample configuration including path length and flow rate where relevant	Flowing capillary – 1.5 mm of Internal Diameter<br>0.075ml/min for batch - 0.3 mL/min for HPLC
Sample temperature (ºC)	25
Software employed for SAS data reduction, analysis, and interpretation	
SAS data reduction to sample–solvent scattering	Foxtrot (in house SWING software developed in collaboration with Xenocs).
**SPring-8 - BL40B2**	
Experiment dates: 23-24 July 2019	
Special sample conditions	

Protein	RNaseA	lysozyme	xylanase	Xylose isomerase
Concentrations for batch mode (mg/mL)	8.31, 4.17	1.52	10.2, 4.95	4.05, 1.93
Notes	No azide was added to samples prior to SAXS measurements			

SAS data collection parameters	
Source, instrument and description or reference	SPring-8 (Hyogo, JAPAN) BL40B2<br>Detector: PILATUS 3S 2M (Dectris)
Wavelength (Å)	1.0
Beam geometry (size, sample-to-detector distance)	Beamsize 0.7 mm (horizontal) x 0.3 mm (vertical)<br>Sample-to-detector distance 1.195 m
q-measurement range (Å^{-1} or nm^{-1})	0.0109 to 0.7825 Å^{-1}
Absolute scaling method	Scaled from 2 mm pure water
Basis for normalization to constant counts	Transmitted intensity by ion-chamber counter
Method for monitoring radiation damage, X-ray dose where relevant	Data frame-by-frame comparison, 150 Gy/sec.
Exposure time, number of exposures	10 s/frame, 3 exposures
Sample configuration including path length and flow rate where relevant	Effective sample path length = 2 mm
Sample temperature (ºC)	25
Software employed for SAS data reduction, analysis, and interpretation	
SAS data reduction to sample–solvent scattering	*I(q)* versus *q* using pyFAI 0.18
**Stanford Synchrotron Radiation Laboratory (SSRL) – Beamline 4-2 BioSAXS**	
Experiment dates: 30 June – 1 July 2019	
Special sample conditions	

Protein		RNaseA	Lysozyme	Xylanase	Urate oxidase	Xylose isomerase
For SEC-SAXS	Loading volume (µl)	n.a.	70 µl	n.a.	50	n.a.
	Loading concentration (mg/mL)	n.a.	10	n.a.	5	
	Flow rate (mL/min)	n.a.	0.05	n.a.	0.05	
Batch measurement concentrations (mg/mL) (estimated from *I*(0) comparisons)		10.0 – 2.5	10.0 – 2.5	10.0 – 2.5	~5 with two serial dilutions	n.a.
Notes		Lysozyme was locally sourced (Chicken egg white Sigma L4919) and measured in 50mM Sodium acetate pH=4.8, 150mM NaCl<br>Azide was added prior to SAXS measurement				
SAS data collection parameters						

<table>
<tr><td>Source, instrument and description or reference</td><td colspan="3">Synchrotron (20-pole, 2.0-Tesla Wiggler), Si(111) monochromator, Beamline 4-2 BioSAXS (https://www-ssrl.slac.stanford.edu/smb-saxs/content/bl4-2)
Detector: Pilatus3 X 1M (Dectris)</td></tr>
<tr><td>Wavelength (Å)</td><td colspan="3">1.12709</td></tr>
<tr><td>Beam geometry (size, sample-to-detector distance)</td><td colspan="3">0.3 mm (horizontal) x 0.3 (vertical) mm, 1.7m</td></tr>
<tr><td>q-measurement range (Å^{-1} or nm^{-1})</td><td colspan="3">0.007 – 0.51</td></tr>
<tr><td>Absolute scaling method</td><td colspan="3">Water scattering</td></tr>
<tr><td>Basis for normalization to constant counts</td><td colspan="3">Transmission intensity measured by photo diode on beamstop.</td></tr>
<tr><td>Method for monitoring radiation damage, X-ray dose where relevant</td><td colspan="3">SASTool; a series of images for buffer and sample (typically 10 - 16) is collected and a variance for each frame calculated for each q-bin as the square difference between the average and the single pixels within that bin. These variances are summed over the whole frame. The variance of the buffer is averaged over the buffer series and the average multiplied by an empirically determined factor (typically 1.3), which is used then as a cut-off value for valid sample frames to include when compared to the first sample frame.</td></tr>
<tr><td>Exposure time, number of exposures</td><td colspan="3">1 sec, 10 exposures</td></tr>
<tr><td>Sample configuration including path length and flow rate where relevant</td><td colspan="3">Sample cell: 1.5mm quartz capillary in diameter
Sample was oscillated at 5 µl/sec during exposures.</td></tr>
<tr><td>Sample temperature (ºC)</td><td colspan="3">23</td></tr>
<tr><td colspan="4">Software employed for SAS data reduction, analysis, and interpretation</td></tr>
<tr><td>SAS data reduction to sample–solvent scattering</td><td colspan="3">Data reduction to background subtraction: SASTool (https://www-ssrl.slac.stanford.edu/smb-saxs/content/documentation/sastool)</td></tr>
<tr><td colspan="4">SANS Data</td></tr>
<tr><td colspan="4">ANSTO Australian Centre for Neutron Scattering, QUOKKA instrument</td></tr>
<tr><td colspan="4">Experiment dates: 19 – 21 July 2019 and 13 Dec. 2021</td></tr>
<tr><td colspan="4">Special sample conditions</td></tr>
<tr><td rowspan="6">Protein concentrations (mg/mL)</td><td>Protein</td><td>H_2O</td><td>D_2O</td></tr>
<tr><td>RNase A</td><td>2.5, 7.7, 3.9</td><td>2.5, 8.1, 4.1</td></tr>
<tr><td>Lysozyme</td><td>2.5, 8.2, 4.1</td><td>2.5, 8.7, 4.6</td></tr>
<tr><td>Xylanase</td><td>10.6, 5.4</td><td>10.3, 5.2</td></tr>
<tr><td>Urate oxidase</td><td>3.4, 1.7</td><td>3.6, 1.8</td></tr>
<tr><td>Xylose isomerase</td><td>1.0, 1.9</td><td>1.0, 2.0</td></tr>
<tr><td>Sample preparation for SANS in H_2O or D_2O</td><td colspan="3">No azide addition required for SANS. All initial sample solutions were filtered through a regenerate cellulose syringe filter with a 0.2 µm pore size, injected onto a Superdex 200 16/600 column and eluted with their respective buffers. Peak fractions were combined and concentrated using a 3500 MWCO Amicon centrifugal at 4000 × g in a fixed angle rotor for 10 mins at a time. The concentrated sample was then dialysed on a 3500 MWCO dialysis cassette against the measurement buffer. Last step dialysates were used for all buffer measurements, and all samples were centrifuged at 12000 × g for 30 mins at room temperature to sediment any aggregate (room temperature centrifugation also assists with degassing). Additional SANS measurements were made on RNaseA and lysozyme (both 2.5 mg/mL) after elution from a SEC S75 10/300 column followed</td></tr>
</table>

<table>
<tr><td></td><td colspan="3">immediately by dialysis and measurement without concentration.</td></tr>
<tr><td colspan="4">SAS data collection parameters</td></tr>
<tr><td>Source, instrument and description or reference</td><td colspan="3">QUOKKA, 40-m SANS instrument.
Detector: 1x1 m^2 ^3He pad detector (Brookhaven),
Further technical specifications at https://www.ansto.gov.au/research/user-office/instruments/neutron-scattering-instruments/quokka/technical-information, reference (Wood et al., 2018)</td></tr>
<tr><td>Wavelength (Å)</td><td colspan="3">6.10 ($\Delta\lambda/\lambda$ = 10% FWHM)</td></tr>
<tr><td>Beam geometry (size, sample-to-detector distances)</td><td colspan="3">Source aperture size 50 mm, sample aperture size 12.5 mm. Source-to-sample and sample-to-detector distances were 5.97 m and 6.033 m, respectively, for q = 0.009 – 0.100 $Å^{-1}$, and 3.969 m and 1.345 m, respectively, for q = 0.05 – 0.45 $Å^{-1}$.</td></tr>
<tr><td>q-measurement range ($Å^{-1}$ or nm^{-1})</td><td colspan="3">Total q-range measured 0.009 – 0.45 $Å^{-1}$.</td></tr>
<tr><td>Absolute scaling method</td><td colspan="3">By normalization to the incident beam flux.</td></tr>
<tr><td>Basis for normalization to constant counts</td><td colspan="3">Raw counts were normalized to monitor counts, transmission scaled and corrected for contributions of the empty cell and blocked beam.</td></tr>
<tr><td>Method for monitoring radiation damage, X-ray dose where relevant</td><td colspan="3">n.a.</td></tr>
<tr><td>Exposure time, number of exposures</td><td colspan="3">Sample in H_2O: For full concentration samples, 1 hour for samples and buffers in low-q setting and 30 mins in high-q setting, twice those times for half concentration samples and buffers.
Samples in D_2O: For full concentration samples, 30 mins in the low-q setting and 15 mins in the high-q setting, twice those times for the half concentration samples.</td></tr>
<tr><td>Sample configuration including path length and flow rate where relevant</td><td colspan="3">Hellma QS-120 cells with a 1 mm path-length for samples in H_2O and a 2 mm path-length for samples in D_2O</td></tr>
<tr><td>Sample temperature (ºC)</td><td colspan="3">15</td></tr>
<tr><td colspan="4">Software employed for SAS data reduction</td></tr>
<tr><td>SAS data reduction to sample–solvent scattering</td><td colspan="3">Igor Pro software (WaveMetrics, Lake Oswego, OR) and the SANS macros developed at the NIST Center for Neutron Research (NCNR) and adapted for QUOKKA were used to reduce raw data to I(q) vs q and merge different detector settings. Solvent scattering was subtracted either using Igor or PRIMUS (ATSAS suite 3.0 (Franke et al., 2017, Manalastas-Cantos et al., 2021)) to yield scattering from the protein alone in each case.</td></tr>
<tr><td colspan="4">Institut Laue-Langevin: D22 – Large Dynamic Ranges Small-Angle Diffractometer</td></tr>
<tr><td colspan="4">Experiment dates: 19 Nov. 2019</td></tr>
<tr><td colspan="4">Special sample conditions</td></tr>
<tr><td rowspan="6">Protein Concentrations for batch-mode measurement (mg/mL)</td><td>Protein</td><td>H_2O</td><td>D_2O</td></tr>
<tr><td>RNase A</td><td>3.6</td><td>3.1</td></tr>
<tr><td>Lysozyme</td><td>10.0, 5.0</td><td>7.7, 5.7</td></tr>
<tr><td>Xylanase</td><td>7.7, 5.3</td><td>6.8, 6.2</td></tr>
<tr><td>Urate oxidase</td><td>1.2</td><td>1.4</td></tr>
<tr><td>Xylose isomerase</td><td>1.0</td><td>2.3</td></tr>
<tr><td rowspan="3">Loading concentration/estimated average measurement concentration for SEC-SANS (mg/mL)</td><td>Protein</td><td>H_2O</td><td>D_2O</td></tr>
<tr><td>RNase A</td><td>16.5/2.8</td><td>16.5/2.4</td></tr>
<tr><td>Lysozyme</td><td>20/1.4</td><td>20/0.6</td></tr>
</table>

	Xylanase	9/1.4	9/1.2
	Urate oxidase	10/0.7	10/0.8
	Xylose isomerase	11/1.2	11/2.0
Sample preparation for SANS in H_2O or D_2O	The standard protocol was used for initial sample preparation but exchange into D_2O was achieved during SEC-SANS and samples were concentrated for batch measurement. SEC flow through was used for solvent measurements.		
SAS data collection parameters			
Source, instrument and description or reference	D22 is a 20-m SANS instrument with SEC-SANS capability (Johansen *et al.*, 2018).<br>Detector: Area multidetector (^{3}He), active area 1 m^2 with a pixel size of 0.8 x 0.8 cm.<br>Detailed specifications https://www.ill.eu/users/instruments/instruments-list/d22/characteristics		
Wavelength (Å)	6 ± 10%		
Beam geometry (size, sample to detector distances)	Rectangular collimation (40 mm x 55 mm), sample aperture: circular 12 mm diameter.		
	Sample-to-detector, collimation distances, batch mode		
	urate oxidase, xylose isomerase	1.5 m S-D, 2.8 m coll	
		11.2 m S-D, 11.2 m coll.	
	RNase A, lysozyme, xylanase	1.5 m S-D, 2.8 m coll.	
		5.6 m S-D, 5.6 m coll.	
	Sample-to-detector, collimation distances, SEC-SANS mode		
	urate oxidase, xylose isomerase	11.2 m S-D, 11.2 m coll. and 1.5 m S-D, 2.8 m coll.	
	RNaseA	1.5 m S-D, 2.8 m coll. and 5.6 m S-D, 5.6 m coll.	
	lysozyme, xylanase	1.5 m S-D, 2.8 m coll.	
q-measurement range (Å^{-1})	Batch mode		
	All proteins	0.01065 – 0.4845	
	SEC-SANS mode		
	RNaseA	0.01179 – 0.536	
	lysozyme and xylanase	0.04013 – 0.536	
	urate oxidase and xylose isomerase	0.00648 – 0.536	
Absolute scaling method	By normalization to the incident beam flux.		
Basis for normalization to constant counts	Raw counts were normalized to monitor counts, transmission scaled and corrected for contributions of the empty cell and blocked beam.		
Exposure time, batch mode	RNase A	3.6 mg/mL H-buffer	30 min
		3.1 mg/mL D-buffer	30 min
	Lysozyme	5 mg/mL H-buffer	18 min
		10.0 mg/mL H-buffer	15 min
		5.7 mg/mL D-buffer	8 min
		7.7 mg/mL D-buffer	7 min
	Xylanase	5.3 mg/mL H-buffer	15 min
		7.7 mg/mL H-buffer	15 min
		6.2 mg/mL D-buffer	8 min
		6.8 mg/mL D-buffer	8 min

<table>
<tr><td rowspan="4"></td><td rowspan="2">Urate oxidase</td><td>1.0 mg/mL D-buffer</td><td>70 min</td></tr>
<tr><td>2.3 mg/mL H-buffer</td><td>25 min</td></tr>
<tr><td rowspan="2">Xylose isomerase</td><td>1.2 mg/mL D-buffer</td><td>100 min</td></tr>
<tr><td>1.4 mg/mL H buffer</td><td>40 min</td></tr>
<tr><td>Sample configuration including path length and flow rate where relevant</td><td colspan="3">1 mm banjo cells, 300 µL volume</td></tr>
<tr><td>SEC-SANS details (type of column, flow rate, etc)</td><td colspan="3">SuperDex 200 increase, 10/300 (24mL), injection 250uL, flow rate 0.15 mL/min during chromatography and 0.015 mL/min during SANS exposure to accumulate sufficient statistics.</td></tr>
<tr><td>Sample temperature (ºC)</td><td colspan="3">8 – 11</td></tr>
<tr><td colspan="4">Software employed for SAS data reduction</td></tr>
<tr><td>Data reduction to I(q) vs q</td><td colspan="3">GRASP (C. Dewhurst), https://www.ill.eu/users/support-labs-infrastructure/software-scientific-tools/grasp/</td></tr>
<tr><td>Solvent subtraction and merging</td><td colspan="3">IGOR data reduction NIST NCNR package (Kline, 2006)
Merging without scaling factor, buffer subtraction without scaling factor, arbitrary constant subtraction, normalisation by concentration (measured by 280nm absorbance)</td></tr>
<tr><td colspan="4">NIST Center for High Resolution Neutron Scattering (CHRNS) NGB 30m SANS Instrument</td></tr>
<tr><td colspan="4">Experiment dates: 10-13 Aug. 2019</td></tr>
<tr><td colspan="4">Special sample conditions</td></tr>
<tr><td rowspan="6">Protein Concentrations (mg/mL)</td><td>Protein</td><td>H_2O</td><td>D_2O</td></tr>
<tr><td>RNase A</td><td></td><td>5.3</td></tr>
<tr><td>Lysozyme</td><td>8.6, 4.1</td><td></td></tr>
<tr><td>Xylanase</td><td>5.0, 3.1</td><td>4.8, 2.9</td></tr>
<tr><td>Urate oxidase</td><td>1.5</td><td>1.6</td></tr>
<tr><td>Xylose isomerase</td><td>2.4, 2.0, 6.8</td><td>2.0, 1.9</td></tr>
<tr><td>Sample preparation for SANS in H_2O or D_2O</td><td colspan="3">Sample preps were the same as for the CHRNS VSANS instrument (see below).</td></tr>
<tr><td colspan="4">SAS data collection parameters</td></tr>
<tr><td>Source, instrument and description or reference</td><td colspan="3">30 meter long Small-Angle Neutron Scattering (SANS) instrument on split neutron guide NGB,
Detector: 640 mm x 640 mm ^{3}He position-sensitive proportional counter with a 5.08 mm x 5.08 mm resolution
https://www.nist.gov/ncnr/ngb-30m-sans-small-angle-neutron-scattering</td></tr>
<tr><td>Wavelength (Å)</td><td colspan="3">6, with a resolution of 12% set by a velocity selector.</td></tr>
<tr><td>Beam geometry (size, sample-to-detector distance)</td><td colspan="3">Beam size was 0.5 inches (1.27 cm) at the sample. Sample-to-detector distances were 1 m, 5 m and 11 m for a q-range of 0.005 Å^{-1} to 0.55 Å^{-1}</td></tr>
<tr><td>q-measurement range (Å^{-1})</td><td colspan="3">q-ranges after buffer subtraction: 0.006 – 0.2 for Xylose isomerase; 0.015 – 0.3 for RNase A, Lysozyme, Xylanase; 0.006 – 0.2 for Urate oxidase.</td></tr>
<tr><td>Absolute scaling method</td><td colspan="3">By normalization to the incident beam flux.</td></tr>
<tr><td>Basis for normalization to constant counts</td><td colspan="3">Raw counts were normalized to monitor counts and corrected for contributions of the empty cell, non-uniform detector response and ambient room background counts</td></tr>
<tr><td>Exposure time, number of exposures</td><td colspan="3">Sample in H_2O: For high concentration samples, 15-20 mins for samples in low-q setting and 0.3 to 1.5 hours in high-q setting, approx. twice those times for half concentration samples and buffers.</td></tr>
</table>

	Samples in D_2O: For high concentration samples, 15-20 mins in the low-*q* setting and 0.3 to 1.5 hours in the high-*q* setting, approx. twice those times for the half concentration samples. Buffers were counted for approx. the same times as the samples
Sample configuration including path length and flow rate where relevant	1 mm pathlength quartz banjo cells. (Volume: : 300 μL)
Sample temperature (ºC)	22
Software employed for SAS data reduction	
Data reduction to sample–solvent scattering and merging	Igor Pro software (WaveMetrics, Lake Oswego, OR) and the SANS macros developed at the NCNR (Kline, 2006)
**NIST Center for High Resolution Neutron Scattering (CHRNS) VSANS Instrument**	
Experiment dates: 9 – 12 August 2019	
Special sample conditions	
Protein Concentrations (mg/mL)	Protein / H_2O / D_2O:<br>RNase A: 5.1 / 5.3<br>Lysozyme: 8.6, 4.1 / —<br>Xylanase: 5.0, 3.1 / 4.8, 2.9<br>Urate oxidase: 1.5 / 1.6<br>Xylose isomerase: 2.4, 2.0, 6.8 / 2.0
Sample preparation for SANS in H_2O or D_2O	No azide was required for SANS. All sample preparations were subjected to SEC protocol following the protocol for the SEC-SANS done at the ILL. (Thus, samples were measured directly after the SEC without performing a dialysis after SEC). Peak fractions from the preparative SEC purifications of Lysozyme, Xylanase, Urate Oxidase, and Glucose Isomerase were further analyzed by analytical HPLC-SEC-MALS to confirm monodispersity and oligomerization state. Separations were performed using a WTC-050N5 column (Wyatt), with in-line DAWN HELEOS-II MALS and Optilab T-rEX Refractive Index detectors. Calculated molar masses from MALS were 11 kDa, 24 kDa, 136 kDa and 168 kDa respectively, consistent with the expected masses for monomeric (Lysozyme and Xylanase) and tetrameric (Urate Oxidase and Glucose Isomerase) species.
SAS data collection parameters	
Source, instrument and description or reference	45 meter long Very Small-Angle Neutron Scattering (VSANS) instrument on neutron guide NG3, https://www.nist.gov/ncnr/chrns-vsans-very-small-angle-neutron-scattering
Wavelength (Å)	6 with a resolution of 12% set by a velocity selector.
Beam geometry (size, sample-to-detector distance)	Beam size was 0.5 inches (1.27 cm) at the sample. Sample-to-detector distances of 2.3 m and 11 m for the two detector carriages, for a *q*-range of 0.005 $Å^{-1}$ to 0.55 $Å^{-1}$
q-measurement range ($Å^{-1}$)	*q*-ranges after buffer subtraction: 0.006 – 0.2 for xylose isomerase; 0.015 – 0.3 for RNase A, lysozyme, xylanase; 0.006 – 0.2 $Å^{-1}$ for urate oxidase.
Absolute scaling method	By normalization to the incident beam flux.
Basis for normalization to constant counts	Raw counts were normalized to monitor counts and corrected for contributions of the empty cell, non-uniform detector response and ambient room background counts
Exposure time, number of exposures	Sample in H_2O: For high concentration samples, 15-20 mins for samples in low-*q* setting and 0.3 to 1.5 hours in high-*q* setting,

	approx. twice those times for half concentration samples and buffers.<br>Samples in D_2O: For high concentration samples, 15-20 mins in the low-*q* setting and 0.3 to 1.5 hours in the high-*q* setting, approx. twice those times for the half concentration samples. Buffers were counted for approx. the same times as the samples.
Sample configuration including path length and flow rate where relevant	1 mm pathlength quartz banjo cells (Volume: 300 μL)
Sample temperature (ºC)	22
Software employed for SAS data reduction	
Data reduction to sample–solvent scattering and merging	Igor Pro software (WaveMetrics, Lake Oswego, OR) and the SANS macros developed at the NCNR (Kline, 2006)

Disclaimer: Certain commercial equipment, materials, software, or suppliers are identified in this table to foster understanding. Such identification does not imply recommendation or endorsement by the National Institute of Standards and Technology, nor does it imply that the materials or equipment identified are necessarily the best available for the purpose.

Table S4 Numbers and types of SAS measurements submitted and used for analysis for each protein

A. SAS measurements submitted for each protein

Protein	SEC-SAXS	Batch SAXS		SEC-SANS		Batch SANS	
		H_2O	D_2O	H_2O	D_2O	H_2O	D_2O
RNase A	8	23	-	1	1	5	6
Lysozyme	9	22	-	1	1	9	5
Xylanase	9	24	-	1	1	8	8
Urate oxidase	10	20	2	1	1	5	5
Xylose isomerase	8	29	7	1	1	9	6

B. SAXS measurements used for analysis provided in main text Table 2 and those combined for final consensus profiles

Protein	SEC-SAXS (Table 2 statistics)	Batch SAXS (Table 2 statistics)	Combined for consensus profile			Total data sets for consensus
			SEC-SAXS/ batch merge	Batch only	SEC-SAXS only	
RNase A	7	9	5	2	2	9
Lysozyme	8	13	1	4	5	10
Xylanase	8	10	2	-	2	4
Urate oxidase*	10	9	6	2	3	11
Xylose isomerase*	8	10	5	6	3	14

*Includes data in H_2O and D_2O

C. SANS measurements used for analysis provided in main text Table 3 and those combined for final consensus profiles

Protein	Data input to *datcombine*		Data merged for a consensus profile (*dc* result = *datcombine* result)	
	H_2O	D_2O	H_2O	D_2O
RNase A	5 batch + 1 SEC-SANS	6 batch	-	SEC-SANS + *dc* result
Lysozyme	9 batch + 1 SEC-SANS	4 batch	-	SEC-SANS + *dc* result
Xylanase	6 batch	6 batch	SEC-SANS + *dc* result	SEC-SANS + *dc* result
Urate oxidase	5 batch + 1 SEC-SANS	5 batch + 1 SEC-SANS	-	-
Xylose isomerase	7 batch (lower conc.)	6 batch	*dc* result + 2 high conc. batch	-

Table S5 Range, spread (Δ), and standard deviation (σ) for R_g values (in Å) from each class of SAXS measurement

Protein	Parameter	Batch-SAXS		SEC-SAXS		Combined-SAXS set	
		R_g range (Δ)	σ	R_g range (Δ)	σ	R_g range (Δ)	σ
RNase A	Guinier R_g	15.25-16.00 (0.75)	0.26	14.94-15.19 (0.25)	0.09	15.00-15.33 (0.33)	0.11
	$P(r)$ R_g	15.01-15.90 (0.89)	0.29	14.99-15.15 (0.16)	0.08	14.95-15.17 (0.22)	0.06
Lysozyme	Guinier R_g	14.46-16.86 (2.40)	0.81	14.08-15.52 (1.44)	0.45	14.08-15.27 (1.19)	0.39
	$P(r)$ R_g	14.36-17.09 (2.73)	0.81	14.16-15.39 (1.23)	0.38	14.21-15.28 (1.07)	0.38
Xylanase	Guinier R_g	16.54-18.15 (1.61)	0.45	15.98-16.65 (0.67)	0.22	15.98-16.21 (0.23)	0.10
	$P(r)$ R_g	16.6-18.43 (1.83)	0.60	15.80-16.91 (1.11)	0.43	15.72-15.93 (0.21)	0.09
Urate oxidase	Guinier R_g	32.77-33.33 (0.56)	0.53	30.84-33.03 (2.19)	0.66	30.95-33.03 (2.08)	0.53
	$P(r)$ R_g	30.77-33.86 (3.09)	0.81	30.11-32.03 (1.92)	0.51	31.51-31.87 (0.36)	0.13
Xylose isomerase	Guinier R_g	32.71-33.74 (1.03)	0.31	32.76-33.46 (0.70)	0.22	32.76-33.77 (1.01)	0.25
	$P(r)$ R_g	32.65-32.82 (0.17)	0.34	32.67-32.93 (0.26)	0.08	32.67-33.08 (0.41)	0.09

Table S6 Comparison of SAXS results for urate oxidase and xylose isomerase in H_2O and D_2O

Units of R_g and d_{max} are Å, V_p is in Å^3. Batch mode measurements were made using a laboratory-based instrument with rotating anode source (NIST/IBBR SAXSLab Ganesha Instrument, 1.4 mg/mL sample) and a synchrotron beam line (Advanced Photon Source – 12-ID-B, 1.0 mg/mL sample). Pairwise CorMAP (Franke *et al.*, 2015) χ^2 and *P* values between H_2O and D_2O measurements are provided after applying scaling and constant adjustment and demonstrate no significant differences over the full extent of the scattering profile. Guinier R_g errors are standard errors from the linear fit.

Protein	Parameter	SAXS in H_2O SAXSLab	SAXS in D_2O SAXSLab	SAXS in H_2O 12-ID-B	SAXS in D_2O 12-12-ID-B
Urate oxidase	R_g Guinier	32.42 ± 0.12	32.49 ± 0.16		
	R_g P(r)	31.77 ± 0.04	31.78 ± 0.04		
	d_{max}	90	91		
	V_P	173703	175538		
	χ^2, *P*-value	0.98, 0.66			
Xylose isomerase	R_g Guinier	33.77 ± 0.16	33.33 ± 0.16	33.09 ± 0.05	33.15 ± 0.06
	R_g P(r)	32.89 ± 0.03	32.92 ± 0.03	32.85 ± 0.02	32.86 ± 0.02
	d_{max}	99	99	99	98
	V_P	236214	235793	229043	227909
	χ^2, *P*-value	0.99, 0.59		1.10, 0.08	

Table S7 Range, spread (Δ), and standard deviations (σ) for R_g values (in Å) for batch SANS in D_2O and H_2O measurements.

Protein	parameter	Batch SANS in D_2O		Batch SANS in H_2O	
		R_g range (Δ)	σ	R_g range (Δ,)	σ
RNase A	Guinier R_g	13.56-14.99 (1.43)	0.52	14.51-15.55 (1.04)	0.39
	$P(r)$ R_g	13.65-14.98 (1.33)	0.45	14.65-15.60 (0.95)	0.40
Lysozyme	Guinier R_g	13.14-13.90 (0.76)	0.33	13.46-15.80 (2.34)	0.68
	$P(r)$ R_g	13.26-13.81 (0.55)	0.25	13.43-15.59 (2.16)	0.69
Xylanase	Guinier R_g	14.70-16.71 (2.01)	0.77	16.39-17.43 (1.04)	0.42
	$P(r)$ R_g	14.44-17.14 (2.70)	1.0	16.39-17.43 (1.04)	0.38
Urate oxidase	Guinier R_g	31.21-35.60 (4.39)	1.9	30.55-32.92 (2.37)	1.0
	$P(r)$ R_g	30.56-30.86 (0.30)	0.42	31.52-34.66 (3.14)	1.34
Xylose isomerase	Guinier R_g	29.58-31.64 (2.06)	0.69	30.88-34.13 (3.25)	0.99
	$P(r)$ R_g	30.37-32.23 (1.86)	0.68	32.08-33.91 (1.83)	0.59

Table S8 Predicted R_g and d_{max} values (in Å) from PDB crystal structure coordinate files described in main text section **3.4** calculated using CRYSOL and CRYSON with no fitting to experiment and R_g values from Guinier fits of the WAXSiS calculated profiles.

	Data	SAXS		SANS			
	Program	CRYSOL	WAXSiS	CRYSON H_2O	CRYSON D_2O	WAXSiS H_2O	WAXSiS D_2O
Protein	Parameter						
RNase A	R_g	15.27	15.09	14.66	13.43	14.50	13.93
	d_{max}	50		50	50		
Lysozyme	R_g	15.14	14.59	14.37	12.24	14.10	12.97
	d_{max}	50		50	50		
Xylanase	R_g	16.44	16.07	15.60	(4.00	15.48	14.89
	d_{max}	47		46	46		
Urate oxidase	R_g	31.72	32.05	31.57	30.84	31.51	31.11
	d_{max}	102		102	102		
Xylose isomerase	R_g	33.09	33.20	32.99	31.65	32.26	31.24
	d_{max}	103		103	103		

CRYSOL/N values are for the atomic structures, including the hydration layer contribution, as reported for R_g from the slope of net intensity with d_{max} corresponding to the envelope diameter. Calculations used default parameters (70 harmonics, order of Fibonacci grid 17).

Table S9 χ^2 values for model fits to data (**Figures 7** and **8**) noting that as a parameter reflective of a global minimum discrepancy, the absolute amplitude of χ^2 is determined by the precision of the data and the propagated statistical errors in the consensus SAXS data are exceptionally small, largest for SANS in H_2O with SANS in D_2O lying in between. Further, χ^2 is not suitable for comparing different methods that refine different types and numbers of parameters to minimize χ^2 against a given data set.

	SAXS				SANS in D_2O			SANS in H_2O		
Protein	WAXSiS	CRYSOL	Pepsi-SAXS	FoXS	WAXSiS	CRYSON	Pepsi-SANS	WAXSiS	CRYSON	Pepsi-SANS
RNase A	65.4	97.0	34.4	121.6	7.4	4.5	3.2	2.0	1.9	2.0
Lysozyme	12.56	25.8	10.6	26.6	2.7	1.8	1.5	2.8	3.9	2.9
Xylanase	8.21	30.5	15.1	17.2	21.3	5.5	7.4	0.8	0.7	0.7
Urate oxidase	11.24	40.8	25.1	19.6	26.1	19.2	15.9	1.0	1.2	1.1
Xylose isomerase	21.8	90.5	26.5	42.1	36.3	7.6	26.8	1.7	6.2	1.9

References

A. Thureau, P. Roblin & Pérez, J. (2021). *J. Appl. Crystallogr.* **54**, 1698-1710.

Abraham, M. J., Murtola, T., Schulz, R., Páll, S., Smith, J. C., Hess, B. & Lindahl, E. (2015). *SoftwareX* **1**, 19-25.

Basham, M., Filik, J., Wharmby, M. T., Chang, P. C., El Kassaby, B., Gerring, M., Aishima, J., Levik, K., Pulford, B. C., Sikharulidze, I., Sneddon, D., Webber, M., Dhesi, S. S., Maccherozzi, F., Svensson, O., Brockhauser, S., Naray, G. & Ashton, A. W. (2015). *J. Synchrotron Radiat.* **22**, 853-858.

Berendsen, H. J. C. P., J. P. M.; DiNola, A.; Haak, J. R. (1984). *J. Chem. Phys.* **81**, 3684-3690.

Blanchet, C. E., Spilotros, A., Schwemmer, F., Graewert, M. A., Kikhney, A., Jeffries, C. M., Franke, D., Mark, D., Zengerle, R., Cipriani, F., Fiedler, S., Roessle, M. & Svergun, D. I. (2015). *J. Appl. Crystallogr.* **48**, 431-443.

Brookes, E. & Rocco, M. (2022). *Sci. Rep.* **12**, 7349.

Brookes, E., Vachette, P., Rocco, M. & Perez, J. (2016). *J. Appl. Crystallogr.* **49**, 1827-1841.

Bussi, G., Donadio, D. & Parrinello, M. (2007). *J. Chem. Phys.* **126**, 014101.

Cantor, C. R. & Schimmel, P. R. (1980). *Techniques for the study of biological structure and function*. San Francisco: W. H. Freeman.

Chatzimagas, L. & Hub, J. S. (2022). *arXiv*, 2204.04961v04961.

Chen, P. C. & Hub, J. S. (2014). *Biophys. J.* **107**, 435-447.

Chen, P. C., Shevchuk, R., Strnad, F. M., Lorenz, C., Karge, L., Gilles, R., Stadler, A. M., Hennig, J. & Hub, J. S. (2019). *J. Chem. Theory Comput.* **15**, 4687-4698.

Classen, S., Hura, G. L., Holton, J. M., Rambo, R. P., Rodic, I., McGuire, P. J., Dyer, K., Hammel, M., Meigs, G., Frankel, K. A. & Tainer, J. A. (2013). *J. Appl. Crystallogr.* **46**, 1-13.

Cohn, E. J. & Edsall, J. T. (1943). *Proteins, amino acids and peptides as ions and dipolar ions*. New York,: Reinhold Publishing Corporation.

Cowieson, N. P., Edwards-Gayle, C. J. C., Inoue, K., Khunti, N. S., Doutch, J., Williams, E., Daniels, S., Preece, G., Krumpa, N. A., Sutter, J. P., Tully, M. D., Terrill, N. J. & Rambo, R. P. (2020). *J. Synchrotron Radiat.* **27**, 1438-1446.

Cromer, D. T. & Mann, J. B. (1968). *Acta Crystallogr.* A**24**, 321-324.

Durchschlag, H. & Zipper, P. (1994). pp. 20-39. Darmstadt: Steinkopff.

Dyer, K. N., Hammel, M., Rambo, R. P., Tsutakawa, S. E., Rodic, I., Classen, S., Tainer, J. A. & Hura, G. L. (2014). *Methods Mol. Biol.* **1091**, 245-258.

Essmann, U., Perera, L., Berkowitz, M. L., Darden, T., Lee, H. & Pedersen, L. G. (1995). *J. Chem. Phys.* **103**, 8577-8592.

Franke, D., Jeffries, C. M. & Svergun, D. I. (2015). *Nat. Methods* **12**, 419-422.

Franke, D., Kikhney, A. G. & Svergun, D. I. (2012). *Nucl. Instrum. Methods Phys. Res. A: Accel. Spectrom. Detect. Assoc. Equip* **689**, 52-59.

Franke, D., Petoukhov, M. V., Konarev, P. V., Panjkovich, A., Tuukkanen, A., Mertens, H. D. T., Kikhney, A. G., Hajizadeh, N. R., Franklin, J. M., Jeffries, C. M. & Svergun, D. I. (2017). *J. Appl. Crystallogr.* **50**, 1212-1225.

Gerstein, M. & Chothia, C. (1996). *Proc. Natl Acad. Sci. U.S.A.* **93**, 10167-10172.

Hajizadeh, N. R., Franke, D. & Svergun, D. I. (2018). *J. Synchrotron Radiat.* **25**, 906-914.

Harding, S. E., Rowe, A. J., Horton, J. C. & Chemistry, R. S. o. (1992). *Analytical Ultracentrifugation in Biochemistry and Polymer Science*. Royal Society of Chemistry.

Hess, B. (2008). *J. Chem. Theory Comput.* **4**, 116-122.

Hopkins, J. B., Gillilan, R. E. & Skou, S. (2017). *J. Appl. Crystallogr.* **50**, 1545-1553.

Hornak, V., Abel, R., Okur, A., Strockbine, B., Roitberg, A. & Simmerling, C. (2006). *Proteins* **65**, 712-725.

Johansen, N. T., Pedersen, M. C., Porcar, L., Martel, A. & Arleth, L. (2018). *Acta Crystallogr.* D**74**, 1178-1191.

Jorgensen, W. L., Chandrasekhar, J., Madura, J. D., Impey, R. W. & Klein, M. L. (1983). **79**, 926-935.

Joung, I. S. & Cheatham, T. E., 3rd (2008). *J. Phys. Chem. B* **112**, 9020-9041.

Kirby, N., Cowieson, N., Hawley, A. M., Mudie, S. T., McGillivray, D. J., Kusel, M., Samardzic-Boban, V. & Ryan, T. M. (2016). *Acta Crystallogr.* D**72**, 1254-1266.

Kirby, N. M., Mudie, S. T., Hawley, A. M., Cookson, D. J., Mertens, H. D. T., Cowieson, N. & Samardzic-Boban, V. (2013). *J. Appl. Crystallogr.* **46**, 1670-1680.

Kline, S. R. (2006). *J. Appl. Crystallogr.* **39**, 895-900.

Knight, C. J. & Hub, J. S. (2015). *Nucleic Acids Res.* **43**, W225-230.

Kuntz, I. D., Jr. & Kauzmann, W. (1974). *Adv. Protein Chem.* **28**, 239-345.

Li, N., Li, X., Wang, Y., Liu, G., Zhou, P., Wu, H., Hong, C., Bian, F. & Zhang, R. (2016). *J. Appl. Crystallogr.* **49**, 1428-1432.

Lindorff-Larsen, K., Piana, S., Palmo, K., Maragakis, P., Klepeis, J. L., Dror, R. O. & Shaw, D. E. (2010). *Proteins* **78**, 1950-1958.

Liu, G., Li, Y., Wu, H., Wu, X., Xu, X., Wang, W., Zhang, R. & Li, N. (2018). *J. Appl. Crystallogr.*

Manalastas-Cantos, K., Konarev, P. V., Hajizadeh, N. R., Kikhney, A. G., Petoukhov, M. V., Molodenskiy, D. S., Panjkovich, A., Mertens, H. D. T., Gruzinov, A., Borges, C., Jeffries, C. M., Svergun, D. I. & Franke, D. (2021). *J. Appl. Crystallogr.* **54**, 343-355.

Miyamoto, S. & Kollman, P. A. (1992). *J. Comput. Chem.* **13**, 952-962.

Panjkovich, A. & Svergun, D. I. (2018). *Bioinformatics* **34**, 1944-1946.

Rocco, M., Brookes, E. & Byron, O. (2020). *Encyclopedia of Biophysics*, edited by G. Roberts & A. Watts, pp. 1-11. Berlin, Heidelberg: Springer Berlin Heidelberg.

Sousa da Silva, A. W. & Vranken, W. F. (2012). *BMC Res. Notes* **5**, 367.

Walker, R. C., Crowley, M. F. & Case, D. A. (2008). *J. Comput. Chem.* **29**, 1019-1031.

Wei, B. Q., Weaver, L. H., Ferrari, A. M., Matthews, B. W. & Shoichet, B. K. (2004). *J. Mol. Biol.* **337**, 1161-1182.
Wood, K., Mata, J. P., Garvey, C. J., Wu, C.-M., Hamilton, W. A., Abbeywick, P., Bartlett, D., Bartsch, F., Baxter, P., Booth, N., Brown, W., Christoforidis, W., Clowes, D., d'Adam, T., Darmann, F., Deura, M., Harrison, S., Hauser, N., Horton, G., Federici, D., Franceschini, F., Hanson, P., Imamovic, E., Imperia, P., Jones, M., Kennedy, S., Kim, S., Lam, T., Lee, W. T., Lesha, M., Mannicke, D., Noakes, T., Olsen, S. R., Osborn, J. C., Penny, D., Perry, M., Pullen, S. A., Robinson, R. A., Schulz, J. C., Xiong, N. & Gilbert, E. P. (2018). *J. Appl. Crystallogr.* **51**, 294-314.
Wu, H., Li, Y., Liu, G., Liu, H. & Li, N. (2020). *J. Appl. Crystallogr.* **53**, 1147-1153.
